# Hydrocarbon-soluble, hexaanionic fulleride complexes of magnesium†
†Electronic supplementary information (ESI) available: Experimental, spectroscopic, crystallographic and computational details (PDF); *xyz* coordinates. CCDC 1914895–1914903. For ESI and crystallographic data in CIF or other electronic format see DOI: 10.1039/c9sc03857d


**DOI:** 10.1039/c9sc03857d

**Published:** 2019-10-09

**Authors:** Samuel R. Lawrence, C. André Ohlin, David B. Cordes, Alexandra M. Z. Slawin, Andreas Stasch

**Affiliations:** a EaStCHEM School of Chemistry , University of St Andrews , North Haugh , St Andrews , KY16 9ST , UK . Email: as411@st-andrews.ac.uk; b Department of Chemistry , Umeå University , Linnaeus väg 10 , Umeå , 907 36 , Sweden

## Abstract

The reaction of the magnesium(i) complexes [{(^Ar^nacnac)Mg}_2_], (^Ar^nacnac = HC(MeCNAr)_2_, Ar = Dip (2,6-iPr_2_C_6_H_3_), Dep (2,6-Et_2_C_6_H_3_), Mes (2,4,6-Me_3_C_6_H_2_), Xyl (2,6-Me_2_C_6_H_3_)) with fullerene C_60_ afforded a series of hydrocarbon-soluble fulleride complexes [{(^Ar^nacnac)Mg}_*n*_C_60_], predominantly with *n* = 6, 4 and 2. ^13^C{^1^H} NMR spectroscopic studies show both similarities (*n* = 6) and differences (*n* = 4, 2) to previously characterised examples of fulleride complexes and materials with electropositive metal ions. The molecular structures of [{(^Ar^nacnac)Mg}_*n*_C_60_] with *n* = 6, 4 and 2 can be described as inverse coordination complexes of *n* [(^Ar^nacnac)Mg]^+^ ions with C_60_^*n*–^ anions showing predominantly ionic metal–ligand interactions, and include the first well-defined and soluble complexes of the C_60_^6–^ ion. Experimental studies show the flexible ionic nature of the {(^Ar^nacnac)Mg}^+^···C_60_^6–^ coordination bonds. DFT calculations on the model complex [{(^Me^nacnac)Mg}_6_C_60_] (^Me^nacnac = HC(MeCNMe)_2_) support the formulation as an ionic complex with a central C_60_^6–^ anion and comparable frontier orbitals to C_60_^6–^ with a small HOMO–LUMO gap. The reduction of C_60_ to its hexaanion gives an indication about the reducing strength of dimagnesium(i) complexes.

## Abstract 2

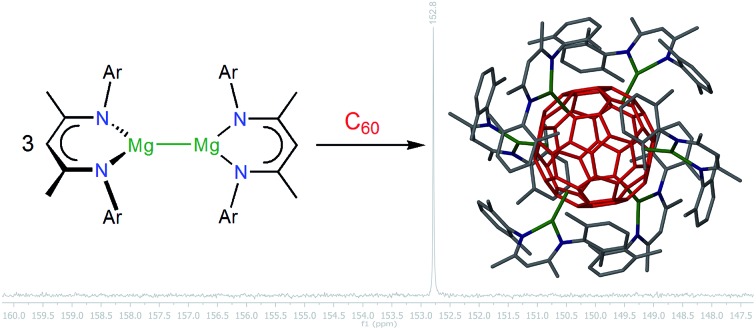
Fullerene C_60_ reacts with dimagnesium(i) compounds LMgMgL, where L is a monoanionic β-diketiminate ligand, to contact ion complexes [(LMg)*_n_*C_60_], where *n* is predominantly 2, 4 or 6.

## Introduction

Dimagnesium(i) compounds LMgMgL, where L is a sterically demanding monoanionic ligand, have been employed as soluble, selective, strong, stoichiometric and safe reducing agents towards a range of organic, organometallic and inorganic substrates.^1^ They have, for example, been employed as reductants to afford compounds with magnesium(i),^2^ zinc(i) and zinc(0),^3^ aluminium(i),^4^ and germanium(0) centres,^5^ as well as reduced anthracene to its dianion, benzophenone to the ketyl radical,^6^ reversibly added to the C=C bonds of selected alkenes,^7^ and after activation reductively coupled CO.^8^ Thus, they can be regarded as strong to very strong reducing agents,^9^ although the reduction potentials of these reagents have not yet been experimentally determined due to decomposition reactions.1*a* Reactions of dimagnesium(i) compounds towards substrates with a known series of reduction potentials could give some indication about the reducing strength of these complexes.

Buckminsterfullerene, C_60_, shows five degenerate orbitals at the HOMO level of *h*_u_ symmetry, and three degenerate orbitals at the LUMO (*t*_1u_) and LUMO+1 (*t*_1g_) level, respectively.^10^–^12^ Fullerenes are generally difficult to oxidise but are readily reduced multiple times.^10^,^13^ In line with having a triply degenerate LUMO level, C_60_ shows six reduction waves from cyclic voltammetry experiments, for example giving *E*_1/2_ of –1.03, –1.44, –1.94, –2.42, –2.91, and –3.28 V relative to Fc^0/+^ (approximately –0.38 V *vs.* SCE or –0.62 V *vs.* SHE)^14^ in toluene/acetonitrile,^15^ with almost equidistant potentials (*ca.* 0.45 V) between reduction steps.^13^ A large number of materials involving fullerene anions (fullerides, fullerenides) have been studied and those with polyanionic fullerides are typically paired with electropositive metals such as alkali metals, alkaline earth metals or selected lanthanoids.^16^,^17^ Alkali metals have especially been used to afford phases such as M_6_C_60_ (M = alkali metal) and even some of composition M_12_C_60_, and others with differing metal-to-fullerene ratios.^13^,^16^,^17^ The C_60_^6–^ anion is a symmetric and diamagnetic species due to the fully filled C_60_ LUMO (*t*_1u_) level. For the rare C_60_^12–^, the former LUMO+1 (*t*_1g_) level is filled,^10^,^13^,^16^,^17^ although complete charge transfer from the metal to the fulleride cannot be certain in some materials. Some reduced fullerene states offer the possibility of different spin states, *e.g.* a singlet *versus* a triplet state for C_60_^2–^.^12^,^13^ A non-symmetric occupation of these orbital levels can effect a Jahn–Teller distortion in the C_60_ framework and this has been observed for all C_60_^*n*–^ ions with 1 ≤ *n* ≤ 5. This is even the case for *n* = 3, which could be expected to prefer a symmetric structure with a degenerate *t*_1u_ level in a quartet state, but instead forms a slightly distorted species with a lower doublet spin state.^13^,^16^ The M_3_C_60_ class materials have received considerable attention because some phases with M = K, Rb, Cs are superconductors.^16^,^17^ Other fulleride materials with s-block metal ions can show structures with C–C bonded polymeric C_60_ units that demonstrate superionic conductivity and are of interest for battery materials.^18^

In addition to insoluble solid state materials, many well-defined exohedral^19^ metal complexes involving metal coordination to neutral fullerenes, fullerides with low charges or fullerene derivatives are known predominantly with transition metals.^20^ Soluble alkali metal complexes with polyanionic fullerides have been prepared and structurally characterised, typically using potassium, rubidium or caesium metal involving solution state chemistry in coordinating solvents and, in some cases, the addition of donor ligands such as crown ethers, cryptands *etc.*^21^–^23^ These experiments formed well-defined isolated complexes with fulleride anions up to C_60_^4–^.^21^,^22^ Furthermore, potassium complexes of C_60_^5–^ have been studied in liquid ammonia^24^ and the reaction of lithium metal with C_60_ in THF afforded solutions showing a sharp ^13^C NMR signal for the C_60_^6–^ anion.^25^ In this work we report on the facile formation of soluble fulleride complexes from reactions of dimeric magnesium(i) compounds LMgMgL with C_60_.

## Results and discussion

### Synthesis

**Fig. 1:**
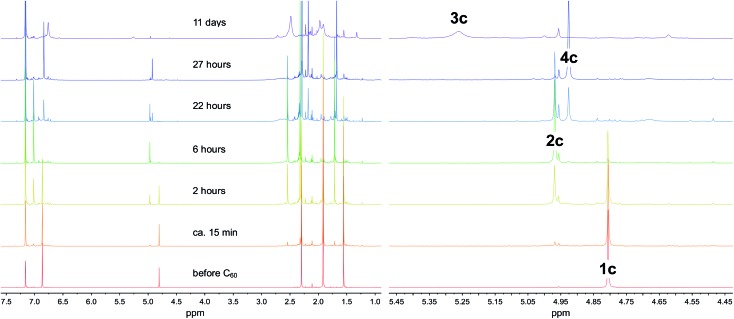
^1^H NMR spectra (500 MHz, 295 K; 1.0–7.5 ppm (left); β-diketiminate *H*C(CMeNMes)_2_ region (right)) of the reaction of 0.5 [{(^Mes^nacnac)Mg}_2_] **1c** with C_60_ in deuterated benzene over time, and the formation of complexes **2c**, **3c** and **4c**.

The most easily accessible dimagnesium(i) compounds are the β-diketiminate complexes [{(^Ar^nacnac)Mg}_2_] **1**, where ^Ar^nacnac = HC(MeCNAr)_2_, and Ar = Dip (2,6-diisopropylphenyl) **1a**,^26^ Dep (2,6-diethylphenyl) **1b**,^27^ Mes (mesityl, 2,4,6-trimethylphenyl) **1c**,^28^ and Xyl (xylyl, 2,6-dimethylphenyl) **1d**.1c,^4^ Treating a partially dissolved mixture of C_60_ in deuterated benzene with [{(^Ar^nacnac)Mg}_2_] **1** at room temperature leads in all cases to a change in colour from the characteristic light purple of the fullerene, to dark brown owing to the formation of [{(^Ar^nacnac)Mg}_*n*_C_60_] species. The reaction mixtures can further change over time as judged by ^1^H NMR and ^13^C{^1^H} NMR spectroscopy, depending on the number of equivalents of **1** used and on the steric demand of the ^Ar^nacnac-ligand in **1**. When an excess of [{(^Mes^nacnac)Mg}_2_] **1c**, *e.g.* six equivalents, is reacted with C_60_, new resonances for a single new product, [{(^Mes^nacnac)Mg}_6_C_60_] **2c**, gradually grow in ^1^H NMR spectra next to those of unreacted **1c**. Reacting only 0.5 equivalents of [{(^Mes^nacnac)Mg}_2_] **1c** with C_60_ in deuterated benzene initially also leads to resonances for the formation of [{(^Mes^nacnac)Mg}_6_C_60_] **2c** (Fig. 1), *e.g.* after six hours, but the composition further changes over time to mixtures of other fulleride complexes [{(^Mes^nacnac)Mg}_*n*_C_60_], 1 ≤ *n* ≤ 5. Of these, [{(^Mes^nacnac)Mg}_2_C_60_] **3c** and [{(^Mes^nacnac)Mg}_4_C_60_] **4c** were found to be dominant species, see Fig. 1, and these were also obtained from reactions with other **1c** : C_60_ ratios. The experiment presented in Fig. 1 also shows that essentially full conversion of **1c** to [{(^Mes^nacnac)Mg}_6_C_60_] **2c** can be achieved (6 hours) before it further reacts with excess C_60_ to afford [{(^Mes^nacnac)Mg}_4_C_60_] **4c** (27 hours), which then slowly further converts to a mixture with a large proportion of [{(^Mes^nacnac)Mg}_2_C_60_] **3c** (11 days). Further conversion was complicated by the poor solubility of the lower substituted fulleride complexes and long reaction times. Generally, the solubility of fulleride complexes within a ligand series increases with a higher number of decorated [(^Ar^nacnac)Mg]^+^ groups. Small quantities of dark precipitates formed in some instances especially when low numbers of equivalents of **1** had been added. This is believed to cause the broadened resonances for [{(^Mes^nacnac)Mg}_2_C_60_] **3c** in Fig. 1 due to the presence of solids in the NMR sample. Further additions of portions of 0.5 equivalents of [{(^Mes^nacnac)Mg}_2_] **1c** to these mixtures followed by equilibration showed the formation of varying quantities of **2c**, **4c**, **3c** and other species over time that ultimately formed [{(^Mes^nacnac)Mg}_6_C_60_] **2c** when three equivalents of **1c** were used (Fig. S47–S49†). Reactions carried out at elevated temperatures of 60–80 °C with an excess of **1c** and C_60_ show that these reactions are faster but do not show easily detectable product formation beyond the composition of [{(^Mes^nacnac)Mg}_6_C_60_] **2c**.

**Scheme 1:**
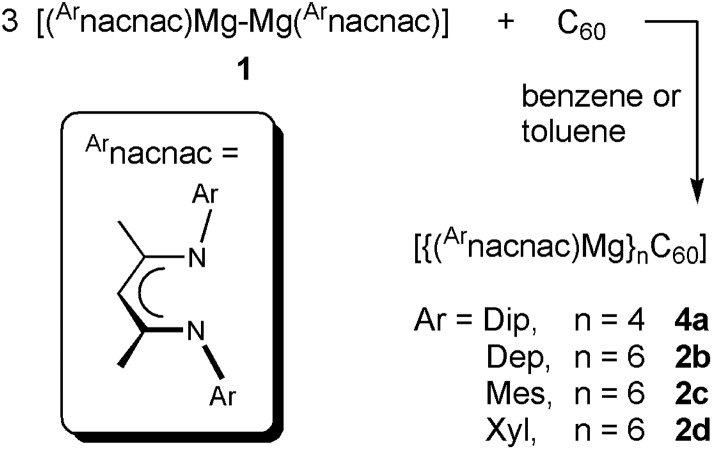
Synthesis of fulleride complexes **2b–d** and **4a**.

Reaction products from comparable experiments for an excess of other [{(^Ar^nacnac)Mg}_2_] **1** with C_60_ were found to be [{(^Dip^nacnac)Mg}_4_C_60_] **4a** for **1a**, [{(^Dep^nacnac)Mg}_6_C_60_] **2b** for **1b**, and [{(^Xyl^nacnac)Mg}_6_C_60_] **2d** for **1d**, see Scheme 1. Titration experiments between C_60_ and [{(^Ar^nacnac)Mg}_2_] **1**, performed by sequentially adding **1** to C_60_ (Fig. S41–S46†), have shown a similar course to those described above for **1c** with comparable dominating species **2** (although **2a** was not observed), **3** and **4** observed in solution. The different composition in **4a** compared to **2b–d** as a final product is likely due to the large steric demand of the Dip group in the series, *vide infra*. A competition experiment of a freshly prepared mixture of three equivalents of [{(^Dip^nacnac)Mg}_2_] **1a** and three equivalents of [{(^Mes^nacnac)Mg}_2_] **1c** with one equivalent of C_60_ only afforded new resonances for [{(^Mes^nacnac)Mg}_6_C_60_] **2c** soon after addition and highlights the importance of steric factors for the activation reaction.

*In situ* preparations of **2–4** often afforded high conversions in solution with a single dominant species only that were characterised by NMR spectroscopy. The formation of these complexes were in some instances accompanied by dark precipitates, especially for products with low solubility. These reactions have been found to be considerably faster in toluene compared with benzene, due to the higher solubility of C_60_ in toluene.^29^ The relatively low solubility of crystalline C_60_, especially in benzene, appears to be one of the main limiting factors for the reaction rates. Finely grinding the fullerene crystals and using sufficiently large solvent volumes accelerate product formation. [{(^Dep^nacnac)Mg}_6_C_60_] **2b** and [{(^Mes^nacnac)Mg}_6_C_60_] **2c** were isolated as crystalline solids in around 50% yield, and [{(^Dep^nacnac)Mg}_2_C_60_] **3b** in 45% yield. In addition, crystalline samples of other compounds were isolated and structurally characterised, *vide infra*.

Assignment of these series of complexes was conducted by recording ^13^C{^1^H} NMR spectra at various points in the study, and through isolated examples characterised by X-ray diffraction, *vide infra*, as complexes of the C_60_^6–^ (**2**), C_60_^2–^ (**3**) and C_60_^4–^ (**4**) anions. Other fulleride species C_60_^*n*–^ as well as dimerised and polymerised fulleride species with C_60_–C_60_ fragments are possibilities for other products or intermediates.^16^,^18^

### Molecular structures from single crystal X-ray diffraction

**Fig. 2:**
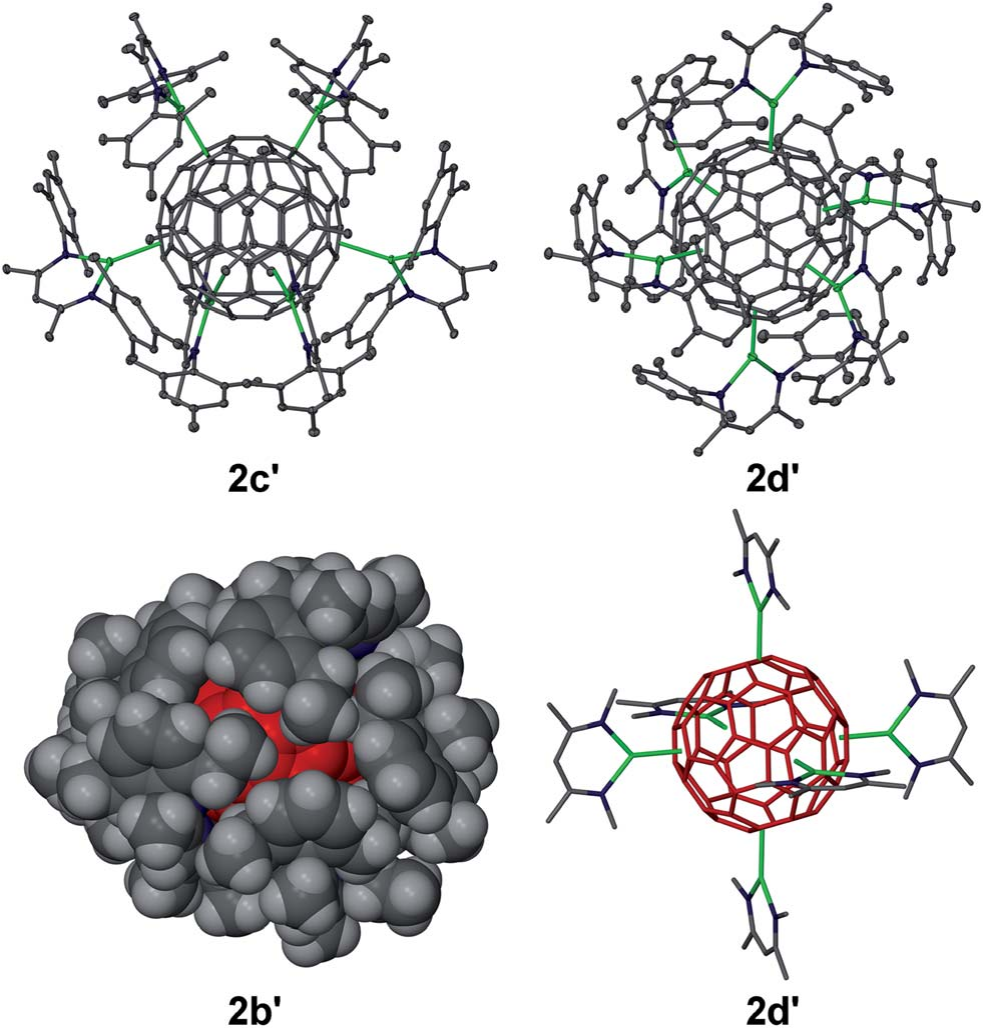
Molecular structures of **2c′** and **2d′** (25% thermal ellipsoids, no H atoms shown), space-filling model of **2b′** (C_60_ unit in red) and core of **2d′** (wire-frame, only aryl *ipso*-carbons shown, no H atoms shown, C_60_ unit in red) showing the near-perfect octahedral coordination. Mg green, N blue, C grey. Solvent molecules omitted for clarity.

**Fig. 3:**
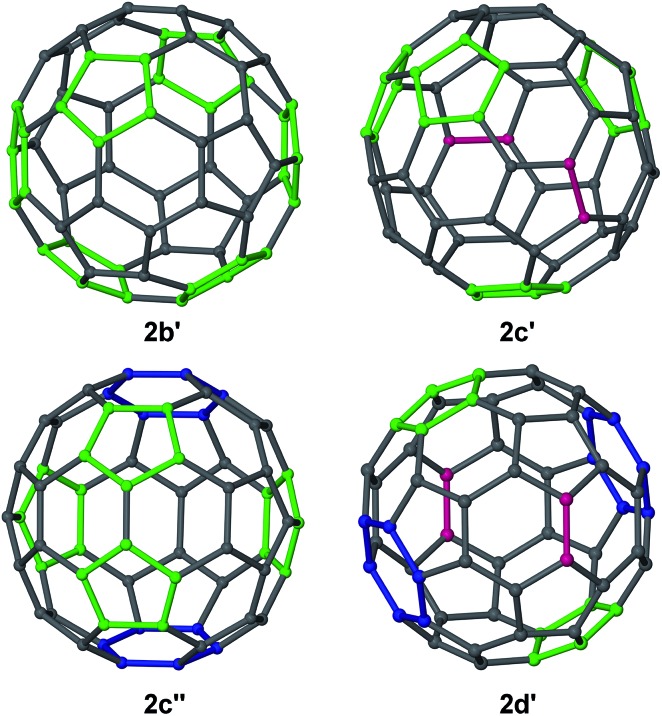
Colour-coded Mg–C_60_ coordination modes in molecular structures of **2b–d**. (^Ar^nacnac)Mg interactions with six-membered (blue) and five-membered (green) rings, and *η*^2^-interactions with [6,6] and [5,6] C–C bonds (pink).

Several complexes [{(^Ar^nacnac)Mg}_6_C_60_] **2** were structurally characterised; namely [{(^Dep^nacnac)Mg}_6_C_60_]·4C_6_H_14_**2b′**, [{(^Mes^nacnac)Mg}_6_C_60_]·7C_6_H_6_**2c′**, [{(^Mes^nacnac)Mg}_6_C_60_]·4C_6_H_14_**2c′′** and [{(^Xyl^nacnac)Mg}_6_C_60_]·7C_6_H_6_**2d′**, see Fig. 2 and the ESI.† These molecular structures show the coordination of six (^Ar^nacnac)Mg fragments around a central C_60_ core with various Mg···C_60_ coordination modes. The (^Ar^nacnac)Mg coordination to C_60_ including their relative positions is presented in colour-coded format in Fig. 3 highlighting *η*^2^, *η*^5^, and *η*^6^ coordination modes. Compounds **2b′** and **2c′** each crystallised with a full molecule in the asymmetric unit. The most sterically crowded derivative **2b′** demonstrates that the six (^Ar^nacnac)Mg units completely wrap-in the C_60_ core, see the space-filling model in Fig. 2 and S60† for that of **2c′**. In **2b′**, all six Mg centres coordinate in a *η*^5^ fashion to five-membered rings of the fullerene. These five-membered rings are all adjacent to each other, *i.e.* only separated by one [6,6] C–C bond each, and form a chiral “ribbon” on the C_60_ surface, see Fig. 3. Solvate **2c′** shows four *η*^5^ Mg···C_60_ interactions to five-membered carbon rings and two *η*^2^ interactions to [5,6] C–C bonds. Two of these Mg centres coordinate to adjacent five-membered rings. Complex **2c′′** crystallised with two full independent molecules in the asymmetric unit showing identical Mg···C_60_ coordination modes. **2c′′** shows four Mg centres coordinate *η*^5^ to five-membered rings, two of these being adjacent to each other, and two coordinate in an *η*^6^ fashion to six-membered carbon rings. The least sterically shielded derivative **2d′** crystallised with half a molecule in the asymmetric unit and shows two *η*^6^, *η*^5^ and *η*^2^ coordination modes each on opposite ends of the C_60_ core (Fig. 3). The structure of **2d′** shows a near-perfect octahedral coordination arrangement of six (^Xyl^nacnac)Mg fragments around the central C_60_ unit, with orthogonal arrangements of (^Xyl^nacnac)Mg ligand planes with respect to neighbouring ligand planes (Fig. 2 and S63†).

The molecular structures **2c′** and **2d′** show the highest bond precisions in the series, well-ordered fulleride units and thus a more detailed analysis is described here. The Mg–N distances (2.006 Å mean in **2c′**, 2.001 Å mean in **2d′**) are short, and for example comparable to those of recently reported cationic [(^Dip^nacnac)Mg·arene]^+^ complexes,^30^ and those in [(^Dip^nacnac)MgPh] with a three-coordinate Mg centre.^31^ The shortest Mg–N distances [*e.g.* Mg(3)–N(47) 1.9805(12) Å and Mg(3)–N(51) 1.9867(12) Å in **2d′**] are those for Mg centres involved in *η*^2^ (^Ar^nacnac)Mg···C_60_ interactions, which supports anticipated ionic Mg···C_60_ bonding interactions, *vide infra*. The Mg···C distances also strongly depend on the coordination mode. For **2d′**, the *η*^6^ [2.074 Å to centroid; Mg–C range: 2.4218(14)–2.6192(14) Å; mean 2.522 Å], *η*^5^ [2.094 Å to centroid; Mg–C range: 2.3656(14)–2.4799(14) Å; mean 2.429 Å], and *η*^2^ [2.140 Å to [6,6] bond midpoint; Mg(3)–C(90) 2.2551(14) Å, Mg(3)–C(97)' 2.2660(14) Å] interactions are relatively short; as expected, shorter than Mg···C distances in the cationic [(^Dip^nacnac)Mg (*η*^6^-mesitylene)]^+^ [2.205 Å to centroid; Mg–C range: 2.5325(17)–2.6988(16) Å; mean 2.612 Å], although longer than Mg–C single bonds, *e.g.* Mg–C: 2.095(3) Å in [(^Dip^nacnac)MgPh].^30^,^31^ The fulleride [5,6] bonds (1.444 Å mean in **2c′**, 1.441 Å mean in **2d′**) are slightly longer than the [6,6] bonds (1.424 Å mean in **2c′**, 1.423 Å mean in **2d′**); the latter are elongated compared to those in free C_60_.^10^,^13^ However, an overlapping range for individual bonds has been found, for example 1.431(2)–1.456(2) Å plus two outliers for [5,6] bonds, and 1.416(2)–1.441(2) Å for [6,6] bonds in **2c′**. The two [5,6] outliers are somewhat longer and are the two C–C bonds that show *η*^2^-coordination to Mg centres [1.469(2) Å to Mg6 and 1.471(2) Å to Mg5]. Similarly, the longest [5,6] bonds in **2d′** [2 × 1.4556(19) Å] are involved in coordinating to Mg centres. The slight lengthening of C–C bonds of fragments that coordinate to Mg centres in comparison to similar uncoordinated fragments is also observed in the mean values of C–C bonds in five-membered carbon rings with (1.448 Å) and without (1.439 Å) *η*^5^ Mg coordination in **2c′**. In **2c′** two Mg centres (Mg1, Mg2) coordinate to two five-membered rings that are only separated by one [6,6] bond and lead to a relatively short Mg···Mg separation of *ca.* 5.69 Å. Similar values are found for related motifs in the other molecular structures of **2**.

**Fig. 4:**
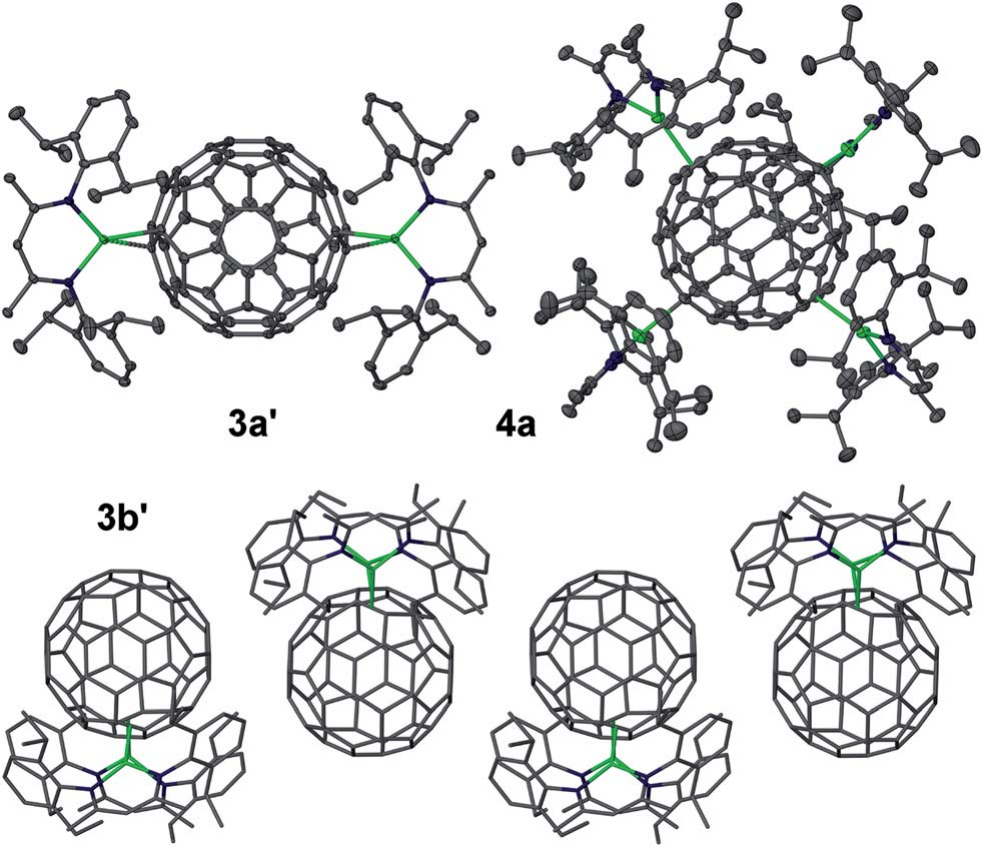
Molecular structures of **3a′** and **4a** (25% thermal ellipsoids, no H atoms shown) and partial packing of **3b′** (wire-frame) showing close C_60_···C_60_ contacts. Mg green, N blue, C grey. Solvent molecules omitted for clarity.

Further molecular structures were determined for [{(^Dip^nacnac)Mg}_2_C_60_]·4C_6_H_6_**3a′**, [{(^Dip^nacnac)Mg}_2_C_60_]·2.5C_6_H_6_**3a′′**, [{(^Dep^nacnac)Mg}_2_C_60_]·1.5C_6_H_6_**3b′**, and [{(^Dip^nacnac)Mg}_4_C_60_] **4a**, see Fig. 4. The molecular structures of **3a′** and **3a′′** are highly similar and only one is presented. The complexes **3a′** and **3a′′** each crystallized with a full molecule in the asymmetric unit and the molecular structures show essentially two terminal (*η*^1^) Mg···C_60_ coordinations with an average Mg–C interaction of 2.212 Å across **3a′** and **3a′′** (*cf.* Mg–C of 2.095(3) Å in [(^Dip^nacnac)MgPh]) and an average sum of the angles around each three-coordinate Mg centre of 360° [359.9(5)°, 360.0(5)°, 359.7(4)°, 359.8(3)°]. The Mg–N bonds (1.975 Å average for **3a′** and **3a′′**) are short. No close C_60_···C_60_ interactions are found in the packing of **3a′**/**3a′′**. In contrast, while the molecular structure of **3b′** shows similar bond lengths to those found for **3a′**/**3a′′**, the two (^Dep^nacnac)Mg units are coordinated to the C_60_ core in a more acute fashion as illustrated by comparison between the Mg···C_60-centre_···Mg angles in **3b′** (*ca.* 79°) and **3a′**/**3a′′** (*ca.* 126°). This allows for some relatively close C_60_···C_60_ interactions from π-stacked one-dimensional fulleride chains along the *b*-axis, arising from short intermolecular C···C interactions (*ca.* 3.4 Å) between slightly offset co-planar five-membered carbon rings from neighbouring C_60_^2–^ moieties. The fulleride C–C bond lengths in **3a′**/**3a′′** do show a wide range of distances, but also a comparatively low bond precision that does not allow a detailed analysis with respect to differences between, for example, [6,6] and [5,6] bonds, coordination between Mg and fulleride unit or Jahn–Teller distortion. Analysis of the distances between fulleride carbon atoms to the fullerene centre in the C_60_^2–^ complex with the highest data quality, **3a′′**, revealed that 58 of them all lie within 0.02–0.03 Å of the mean value (3.54 Å) and no clear and significant pattern for a fulleride distortion emerged. Jahn–Teller distortion in fullerides is relatively small, but can in some cases be observable by structural studies.^13^,^32^ We did, however, observe that the two carbon centres that terminally coordinate the Mg centres are significantly more pyramidalized, slightly protrude from the fulleride “sphere” and show C···C_60-centroid_ distances that are 0.09–0.10 Å longer than the mean from the remaining 58 distances. Also, the fulleride C–C bond lengths in **3b′** cannot be analysed due to poor ordering and low bond precision.

Complex [{(^Dip^nacnac)Mg}_4_C_60_] **4a** crystallised with half a molecule in the asymmetric unit and suffers from fulleride disorder, meaning that the Mg···C_60_ coordination modes cannot be commented on. The four (^Dip^nacnac)Mg units are approximately arranged in a “square planar” fashion around the C_60_ core, albeit with significant tetrahedral distortion. This results in two small (93° mean) and one large (155° mean) Mg···C_60-centre_···Mg angle for each Mg centre (see Fig. S70†).

The combined analysis of the molecular structures of **2–4** shows that many Mg···C_60_ coordination modes (*η*^1^, *η*^2^, *η*^5^, *η*^6^) are possible and that all fulleride fragments (carbon atoms, [6,6] bonds, [5,6] bonds, five-membered rings, six-membered rings) can be involved in bonding to a metal. This, together with bond distance considerations, suggest ionic Mg···C_60_ interactions, *i.e.* that **2–4** are coordination complexes of *n* [(^Ar^nacnac)Mg]^+^ with central C_60_^*n*–^ ions. These can be regarded as “inverse coordination compounds”^33^ where cationic LMg^+^ “ligands” coordinate to a central C_60_^*n*–^ “superatom”.^34^ The molecular structure **2d′** shows a near-perfect octahedral coordination mode of six [(^Xyl^nacnac)Mg]^+^ around the C_60_^6–^ unit with an orthogonal arrangements of ligand planes to those of their neighbours. This arrangement is comparable in overall structure to, for example, those in the transition metal complexes M(NMe_2_)_6_ (M = Mo, W) with inverted charges.^35^ Other coordination geometries can be regarded as distorted octahedral (**2**), distorted square-planar (**4a**) and bent (**3**).

### Spectroscopic and physical properties

^1^H NMR spectra for [{(^Mes^nacnac)Mg}_6_C_60_] **2c** and [{(^Xyl^nacnac)Mg}_6_C_60_] **2d** in deuterated benzene or toluene show sharp resonances for one ligand environment, respectively, *e.g.* see Fig. 1 (6 hours), Fig. S29 and S35.† This is expected for coordination compounds with ionic metal–ligand interactions given the various solid state coordination modes for [(^Ar^nacnac)Mg]^+^ fragments coordinating to C_60_^6–^ ions and the expected low energy processes for interconversions between them. ^1^H NMR spectra obtained from dissolving crystalline samples of [{(^Dep^nacnac)Mg}_6_C_60_] **2b**, however, always afforded resonances for a mixture of species (Fig. S6, S7, S9 and S10†). This included samples that showed a satisfactory microanalysis for **2b**. These spectra mainly show resonances assigned to [{(^Dep^nacnac)Mg}_6_C_60_] **2b**, [{(^Dep^nacnac)Mg}_4_C_60_] **4b**, and the tentatively assigned species [{(^Dep^nacnac)Mg}_5_C_60_] **5b** with significantly broadened resonances (*e.g.* Fig. S24 and S25†). At elevated temperatures in deuterated toluene, some broadened resonances only slightly sharpen and a mixture of species is still observed. Further addition of [{(^Dep^nacnac)Mg}_2_] **1b** to this sample and recording an ^1^H NMR spectrum at 100 °C showed that both compounds **2b** and **1b** together with other fulleride complexes (such as **5b**) are present demonstrating the difficulty to fully reduce C_60_ to C_60_^6–^ using [{(^Dep^nacnac)Mg}_2_] **1b**. This is in line with the highly crowded ligand sphere in the molecular structure of **2b**. Recording spectra of **2b** immediately after dissolution showed the highest concentration of **2b**, followed by growing resonances for **5b**, then **4b** (Fig. S6–S11†). Trace quantities of **1b** were also found in these spectra though these do not account for all the (^Dep^nacnac)Mg fragments removed from **2b**. For comparison, [{(^Ar^nacnac)Mg}_2_] **1** forms equilibria with selected alkenes and alkynes such as 1,1-diphenylethene to novel 1,2-dimagnesioethane species,^7^ and a related equilibrium process may be in operation for **2b**, plus follow-on reactivity forming **5b**, **4b**, **1b** and other species. ^1^H NMR spectra for [{(^Ar^nacnac)Mg}_2_C_60_] **3** (Fig. S2, S16, S18, S31 and S37†) and [{(^Ar^nacnac)Mg}_4_C_60_] **4** (Fig. S4, S22, S33 and S39†) complexes also show resonances for one ligand environment. Poorly soluble complexes **3c**,**d** show somewhat broadened resonances. The addition of donor solvents such as THF to solutions of [{(^Ar^nacnac)Mg}_6_C_60_] **2** typically leads to the precipitation of insoluble materials from solution, likely ionic complexes.

^13^C{^1^H} NMR spectra of the fulleride complexes in deuterated benzene or toluene each show resonances for one ligand environment and one resonance for the fulleride ion as expected for complexes with ionic bonding interactions. The fulleride resonances of the complexes (Table 1) are all downfield shifted compared to that of neutral C_60_ (143 ppm).^13^ The C_60_^6–^ complexes **2b–d** show a sharp resonance at around 153 ppm that is close to that observed for C_60_^2–^ species **3a–d** (*ca.* 156 ppm). For comparison, previously a solution of Li_6_C_60_ could be generated in deuterated THF that showed a sharp resonance at 156.7 ppm.25*a* A broad resonance for C_60_^4–^ species **4a–d** (*ca.* 166 ppm) appears significantly further downfield, and a very broad resonance at *ca.* 174 ppm was tentatively assigned to the putative C_60_^5–^ species **5b**.

**Table 1:** Solution fulleride ^13^C{^1^H} NMR resonances (in ppm) of compounds **2–5**

[{(^Ar^nacnac)Mg}_*n*_C_60_]	Ar = Dip (**a**)	Ar = Dep (**b**)	Ar = Mes (**c**)	Ar = Xyl (**d**)
*n* = 6 (sharp) **2**	—	153.2	152.9	152.8
*n* = 5 (very broad) **5** (?)	—	174 (?)	—	—
*n* = 4 (broad) **4**	166.2	167.3	166.2	166.4
*n* = 2 (sharp to broad) **3**	155.4	156.5	156.3	156.2

All observed fulleride complexes **2–5** show ^1^H and ^13^C{^1^H} NMR resonances for highly symmetric species in solution at room temperature. In addition, NMR spectra for the most soluble series (Ar = Dep), [{(^Dep^nacnac)Mg}_6_C_60_] **2b**, [{(^Dep^nacnac)Mg}_2_C_60_] **3b**, and [{(^Dep^nacnac)Mg}_4_C_60_] **4b**, were recorded at –80 °C in deuterated toluene (Fig. S12–S15, S20 and S21†) and all support symmetric solution behaviour at this temperature. ^1^H NMR data show as expected one ligand backbone CH resonance for each complex, though broader resonances indicate a more hindered rotation of the ethyl groups in the ligand sphere and/or a reduced solubility. The low temperature ^13^C{^1^H} NMR spectra for **2b**, **3b** and **4b** all show a fulleride resonance that is essentially unchanged with respect to its room temperature chemical shift. Also, the sharp appearance of the ^13^C{^1^H} NMR fulleride singlet of **3b** and the broad signal shape for **4b** are virtually unchanged when compared to their room temperature spectra and support highly fluxional behaviour at this temperature. The ^13^C{^1^H} NMR fulleride resonance of **2b**, however, is broadened or starts to split into individual resonances (Fig. S13†). This could have several reasons and could signify splitting of fulleride resonances between different coordination modes and/or coordinated *versus* uncoordinated carbon centres amplified by the extreme ligand crowding in the complex, or simply result from the low solubility under these experimental conditions.

The ^13^C NMR fulleride data found for complexes **2** is very close to those reported for M_6_C_60_ (M = alkali metal cations) solid state materials.^13^,^16^,^36^ The data deviates significantly for complexes **3** (by *ca.* 25–30 ppm) and **4** (by *ca.* 15–20 ppm) compared to those reported for related solid state materials and soluble ionic fulleride complexes in coordinating solvents. These generally show ^13^C NMR chemical shifts for C_60_^2–^ and C_60_^4–^ species of approximately 180–185 ppm; species with C_60_^–^ and C_60_^3–^ ions can show even further downfield shifts.^13^,24b In general it has been suggested that a larger downfield shift can be associated with a higher paramagnetism in fullerides,^13^ and that species with the same number of unpaired electrons may show ^13^C NMR chemical shift in a similar region.^13^,^16^ Comparisons with ^13^C NMR chemical shifts available for solid state materials may be complicated due to contributions from the conduction electrons (Knight shifts) and magnetic coupling between fulleride anions with close contacts in the solid state.^13^ Comparisons to data from solid state materials or from species in coordinating solvents may in some cases be difficult due to possible signal averaging from exchange reactions between fullerides of different charge states.^10^,^13^ The fulleride complexes reported herein, at least those with higher charges such as **2** and **4**, likely suppress direct C_60_^*n*–^···C_60_^*n*–^ interactions; preventing close approach by the large organic ligand sphere as an outer separating layer (Fig. 2). This, together with the use of non-coordinating hydrocarbon solvents, will also likely suppress electron exchange reactions such as disproportionations. Thus, our reported ^13^C{^1^H} NMR resonances are likely not averages from different species and show unchanged chemical shifts for individual species in titration experiments. Comparisons of these with ^13^C NMR data from solid state materials or for alkali metal species in coordinating solvents may allow further conclusions to be drawn with respect to Knight shifts, electronic states or other effects in the solid or solution state.

The [{(^Ar^nacnac)Mg}_6_C_60_] complexes **2** are diamagnetic species with sharp C_60_^6–^ resonances. [{(^Ar^nacnac)Mg}_*n*_C_60_] complexes with uneven numbers of *n* are inevitably paramagnetic species where issues such as significant signal broadening and shifting of resonances in NMR spectra can be expected. For species with 2 ≤ *n* ≤ 4 several spin states are possible such as high and low spin configurations.^10^,^12^,^13^ We used Evan's method^37^ to shed further light on the number of unpaired electrons and the dominating spin state for these species in solution (Fig. S43–S49†). Although we could not yet study solutions of [{(^Ar^nacnac)Mg}_2_C_60_] **3** or [{(^Ar^nacnac)Mg}_4_C_60_] **4** that were completely free of impurities, and these fullerides may be in equilibrium with others of different charges as is known for fullerides in coordinating solvents, these experiments indicated a negligible to very low magnetic moment in solution (*e.g.* Fig. S47 and S48†). This suggests that these species are dominated by a diamagnetic ground state and that all electrons are paired and/or antiferromagnetically coupled in an open shell singlet state. Previous studies on C_60_^2–^ species suggest close singlet and triplet states that are partially populated; data for C_60_^3–^ is consistent with a doublet state (typically described as a low spin species), and a triplet state is suggested for C_60_^4–^.^13^,^22^,^38^ A multiconfigurational description for some species may be warranted and results in a complicated picture. The possibility that C_60_^2–^ complexes **3** and C_60_^4–^ complexes **4** are essentially diamagnetic suggests that these complexes could be in different electronic states to those of known ionic fulleride species, and requires further investigation. In contrast and for comparison, mixtures showing resonances for the tentatively assigned species [{(^Dep^nacnac)Mg}_5_C_60_] **5b**, which has to be a paramagnetic species, show a large relative paramagnetism from similar experiments in solution (Fig. S46a†). A diamagnetic state for **3** and **4** could furthermore explain the significant difference in their ^13^C NMR fulleride chemical shifts to those of related reported materials with significant upfield shift; especially the resonances for **3** which are shifted by 25–30 ppm compared with known materials and are very close to those of diamagnetic C_60_^6–^ in **2**. The further downfield shift and fulleride signal broadening of C_60_^4–^ species **4** could hint at a slightly higher average paramagnetic character despite only very small solution magnetic moments or hint at an open shell singlet state. In this context it is worth noting that the fulleride ^13^C chemical shifts and line shapes for **3b** and **4b** were essentially unchanged when data from room temperature and –80 °C experiments were compared.

All fulleride complexes observed herein are different shades of deep brown in solution. Complexes **2** show an orange-brown colour in solution, complexes **3** show a more green-brown colour. UV/Vis spectra of **2c** (Fig. S1†) recorded in hydrocarbon solution (*n*-hexane or toluene) show some absorption across the visible spectrum with an absorption peak at 428 nm (*ε* ≈ 12 800 mol^–1^ dm^3^ cm^–1^) and a rise in absorption towards the NIR region between 700 and 800 nm; the latter being the limit of the experiment. The absorption maximum is in line with an orange tint and the rise in absorption at longer wavelengths contributes to the overall brown colour.

### Computational studies

Density functional theory (DFT) studies at the pbe0/def2-svp level of theory on the model complex [{(^Me^nacnac)Mg}_6_C_60_] **2Me** (^Me^nacnac = HC(MeCNMe)_2_), C_60_, C_60_^6–^ and [(^Me^nacnac)Mg]^+^, were carried out to shed further light on the bonding situation of complexes **2b–d**. Four isomers were optimised from core coordinates of X-ray solid state structures (Fig. S71–S74†); **2Me-1** (using **2c′** as a starting geometry), **2Me-2** (from **2b′**), **2Me-3** (from **2c′′**) and **2Me-4** (from **2d′**). In each case, the [(^Me^nacnac)Mg]^+^ groups underwent some repositioning in part due to the smaller steric demand of the ligand sphere in isomers of **2Me**. Isomers **2Me-1** to **2Me-3** lie within 3 kcal mol^–1^ of one another and show a combination of *η*^5^ and *η*^2^ Mg coordination modes (Table S2†), whereas isomer **2Me-4** is *ca.* +5 kcal mol^–1^ above the lowest isomer **2Me-3** and shows one terminal (*η*^1^) Mg coordination mode not found in the solid state structures of **2**. *η*^6^ Mg coordination modes, as observed in structures **2c′′** and **2d′**, were not found in isomers of **2Me**. The fulleride C–C bond lengths in isomers of **2Me** show variations within the [5,6] and [6,6] bonds and are affected by Mg coordination. Bond lengths for *η*^2^ Mg coordinated C–C bonds are *ca.* 0.02 Å longer for [5,6] bonds in **2Me-1**, and *ca.* 0.04 Å longer for [6,6] bonds in **2Me-4**, compared to uncoordinated examples in the same respective optimised structure, and supports the trend found in the solid state structures of **2**. Isomer **2Me-2** contains six five-membered carbon rings coordinated to Mg centres and six not coordinated to Mg centres. The former C–C bond lengths (mean 1.445 Å) are on average slightly longer than the latter ones (mean 1.438 Å) and these values are highly comparable to those obtained from the mean measured bond lengths in **2c′**, *cf.* 1.448 Å and 1.439 Å, respectively. The sum of the three C–C–C angles around the carbon atom with *η*^1^ Mg coordination (*ca.* 341°) in **2Me-4** is *ca.* 6° smaller than the mean from comparable uncoordinated carbon atoms in this isomer showing the trend towards pyramidalization and echoes the findings for the carbon atoms with *η*^1^ Mg coordination in the solid state structure of the C_60_^2–^ species **3a′′**.

**Fig. 5:**
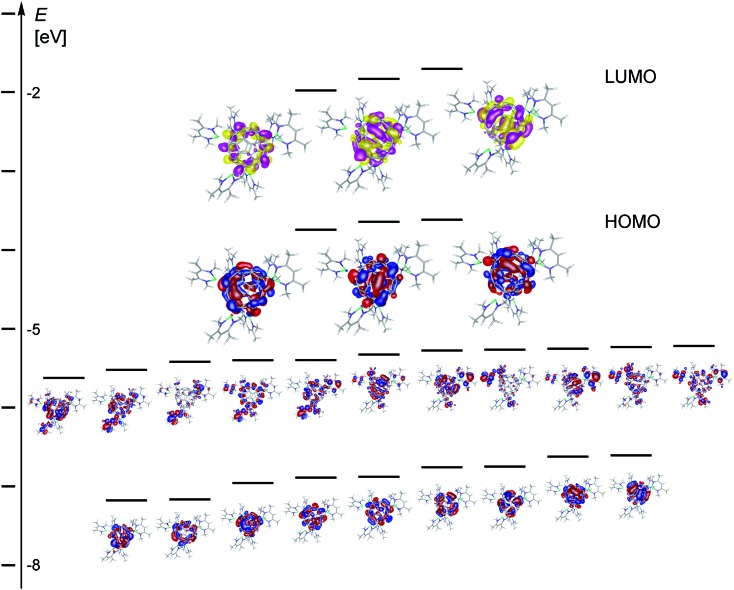
Partial molecular orbital diagram for **2Me-1** (pbe0/def2-svp) showing selected energy levels and molecular orbitals. Orbitals for filled levels are shown in blue and red, and in pink and yellow for unoccupied orbitals (isovalue 0.02 e a.u.^–3^).

A partial molecular orbital diagram of **2Me-1** (Fig. 5) supports the formulation of a complex with a central C_60_^6–^ ion. The frontier orbital HOMO and LUMO levels of **2Me-1** are entirely C_60_ based and largely resemble those of C_60_^6–^ with three occupied (*t*_1u_ in Hückel theory) and three unoccupied (*t*_1g_) molecular orbitals.^10^–^12^ The HOMO–LUMO gap in **2Me-1** is small (1.64 eV, 158 kJ mol^–1^, 37.8 kcal mol^–1^) and similar to that calculated for C_60_^6–^ (1.78 eV) at the same DFT level (Fig. S77†). Below the HOMO level in **2Me-1** is a band of eleven orbitals that appear to originate from mixing of five C_60_*h*_u_ orbitals (the former C_60_ HOMO level or the C_60_^6–^ HOMO-1 level) and six [(^Me^nacnac)Mg]^+^ HOMO orbitals (Fig. S78†). The band of nine orbitals below are C_60_ based and largely represent the four *g*_g_ and five *h*_g_ orbitals that occur at almost identical energy in C_60_ or C_60_^6–^, respectively (*e.g.* Fig. S76 and S77†). Approximately 1 eV above the LUMO level of **2Me-1** starts a band of 14 orbitals that appear to originate from mixing eight C_60_ based orbitals (5 *h*_g_ and 3 *t*_2u_) with six [(^Me^nacnac)Mg]^+^ LUMO+1 orbitals (Fig. S75†). The gap between HOMO and LUMO+1 level approximately corresponds in energy to the UV absorption at 428 nm and the rise in absorption around 700–800 nm to the HOMO–LUMO gap. In relation to the relatively small HOMO–LUMO gap determined for **2Me-1** it is worth mentioning that the C_60_^6–^ anion shows a significantly higher aromatic character than C_60_ with strong diatropic ring currents and a huge endohedral shielding as has been determined for ^3^He@C_60_^6–^ using ^3^He NMR spectroscopy.^10^,^11^,^25^

**Fig. 6:**
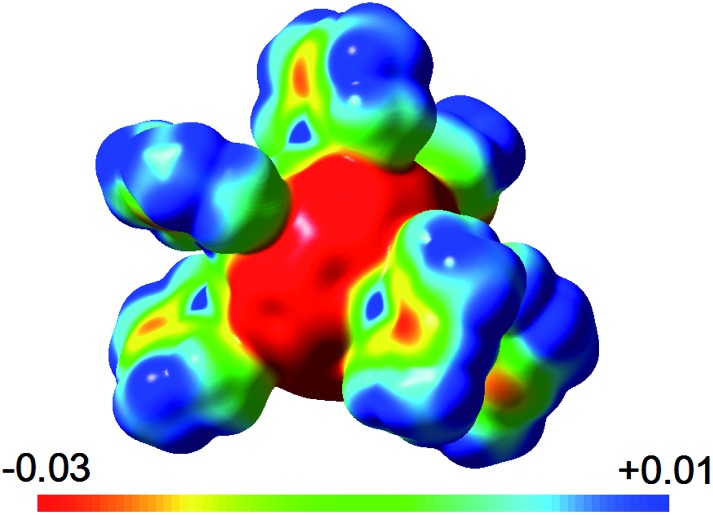
Electrostatic potential (in a.u.) for **2Me-2**.

The calculated Natural Bond Orbital charges for the isomers of **2Me** show some variation of charges on C_60_ carbon atoms that depend on nearby Mg coordination. In general, low hapticity Mg coordination leads to more localised anionic charge. For example, the terminal (*η*^1^) carbon coordinated to Mg in **2Me-4** carries a charge of –0.41; its three direct neighbours only show an average of –0.06. In **2Me-3**, the average charge of an *η*^2^ coordinated carbon atom is *ca.* –0.28 and its four neighbours have a significantly lower charge of *ca.* –0.02. Less polarisation of the central C_60_ unit is found around *η*^5^ coordination modes. This charge accumulation in Mg-coordinated fragments is accompanied by the previously discussed C–C bond lengthening and pyramidalization of carbon centres. For comparison, the expected average charges of 0.00 for C_60_ and –0.10 for C_60_^6–^ with very little to no variation are found. The Mg ions in **2Me** show a charge of approximately +1.77, similar to that in [(^Me^nacnac)Mg]^+^. The sum of charges on C_60_ of –5.8 in **2Me-3** is close to the ideal value of –6 for a wholly ionic system, and supports the view as a contact ion complex with a central C_60_^6–^ ion coordinating to six [(^Me^nacnac)Mg]^+^ ions. Similarly, the electrostatic potential visualises this situation (Fig. 6), and highlights the difference in potential between the highly anionic fulleride unit (red) and positive [(^Me^nacnac)Mg]^+^ fragments (blue sections), especially the strongly positive Mg^2+^ centres, that help balance and dissipate the accumulated charges by coordination.

In order to further probe the nature of the interactions in complexes **2**, we reacted [{(^Mes^nacnac)Mg}_6_C_60_] **2c** with six equivalents of [(^Xyl^nacnac)Li] and observed the very rapid formation of the magnesium(ii) complex [(^Mes^nacnac)Mg(^Xyl^nacnac)] **6**, as suggested by ^1^H, ^7^Li and ^13^C{^1^H} NMR spectroscopy (Fig. S52–S56†), and the formation of an insoluble dark orange-brown precipitate, likely Li_6_C_60_. The rapid and facile breaking of the {(^Mes^nacnac)Mg}^+^···C_60_^6–^ coordination bonds in this reaction is further support for the flexible, ionic nature of these interactions and suggests that complexes **2** can serve as soluble sources of C_60_^6–^.

## Conclusion

Dimagnesium(i) complexes LMgMgL, L = ^Ar^nacnac, react with C_60_ to form a range of hydrocarbon-soluble fulleride complexes of the general formula [(LMg)_*n*_C_60_] and can reduce C_60_ up to its hexaanion if sterics permit. The combined experimental and computational studies for *n* = 6 support the formulation as an inverse coordination complex with flexible ionic LMg^+^ to C_60_^6–^ interactions, and a small HOMO–LUMO gap. The Mg···C_60_ interactions can show a wide range of coordination modes that are easily distorted and represent relatively weak electrostatic interactions. The anions C_60_^2–^, C_60_^4–^ and C_60_^6–^ were found to be dominant species and were characterised in the solution and solid state. C_60_^6–^ complexes **2** are as expected diamagnetic and indication for C_60_^2–^ in complexes **3** and C_60_^4–^ in complexes **4** suggest a dominating diamagnetic ground state. The ease of reducing C_60_ to its hexaanion suggests that the reducing capabilities of LMgMgL compounds towards substrates are at least in the order of *E*^0^ ≤ –2.9 V (*vs.* SCE) or ≤–2.65 V (*vs.* SHE) and thus can be regarded as very strong reducing agents.^9^ This can approximately be compared to the reported reduction potentials of Mg^0/I^ (Mg^+^ + e^–^ → Mg: *E*^0^ = –2.70 V *vs.* SHE; *cf.* Mg^2+^ + 2 e^–^ → Mg: *E*^0^ = –2.372 V *vs.* SHE) and Na metal (Na^+^ + e^–^ → Na: *E*^0^ = –2.71 V *vs.* SHE);^39^ the latter can be used as a reducing agent in preparing LMgMgL complexes. In addition to thermodynamic considerations such as reduction potentials, kinetic factors, for example an appropriate set of sterics and a suitable mechanism, are important in the chemistry of LMgMgL complexes.

## Conflicts of interest

The authors declare no conflict of interest.

## Supplementary Material

Supplementary information

Supplementary information

Crystal structure data
