# Supplementary material for: Hydrocarbon-soluble, hexaanionic fulleride complexes of magnesium
Source: Chem Sci. 2019 Oct 9;10(46):10755–64. doi: 10.1039/c9sc03857d (PMC6993810; doi:10.1039/c9sc03857d)
Supplement: Supplementary file 2 [file SC-010-C9SC03857D-s002.pdf]

Electronic Supplementary Material (ESI) for Chemical Science.
This journal is © The Royal Society of Chemistry 2019

Electronic supplementary information

Hydrocarbon-soluble, hexaanionic fulleride complexes of magnesium

Samuel R. Lawrence, C. André Ohlin, David B. Cordes, Alexandra M. Z. Slawin, Andreas Stasch*

Table of Contents

1	Experimental Section	2
2	NMR spectroscopy	16
3	X-ray crystallography	57
4	Computational studies	78
5	References	89

1 Experimental Section

1.1 General considerations

All manipulations were carried out using standard Schlenk and glove box techniques under an atmosphere of high purity argon or dinitrogen. Benzene, toluene, *n*-hexane and *n*-pentane were either dried and distilled under inert gas over $LiAlH_4$, sodium or potassium, or taken from an MBraun solvent purification system and degassed prior to use. ^{1}H and $^{13}C\{^{1}H\}$ NMR spectra were recorded on a Bruker AV 300, Bruker AVII 400 or Bruker AV III 500 spectrometer in deuterated benzene or toluene and were referenced to the residual ^{1}H or $^{13}C\{^{1}H\}$ resonances of the solvent used. ^{7}Li NMR spectra were recorded on a Bruker AVII 400 spectrometer in deuterated benzene and are referenced against an external aqueous LiCl solution. Yields or conversions in solution were determined by integration of ^{1}H NMR spectra against an internal standard (such as residual C_6D_5H from deuterated benzene, added hexamethylbenzene etc). Note that several *in situ* reactions afforded dark (typically black-brown) reaction mixtures containing (final or intermediate) $[\{(^{Ar}nacnac)Mg\}_nC_{60}]$ complexes of typically low charge fullerides that are poorly soluble and formed a dark precipitate during reactions. Thus not all $(^{Ar}nacnac)Mg$ units contributed to the given solution yields or conversions even though they represented the dominating species in solution. For comparison, starting materials $[\{(^{Ar}nacnac)Mg\}_2]$ **1** and complexes $[\{(^{Ar}nacnac)Mg\}_6C_{60}]$ **2b-d** showed good solubility in these experiments. Selected NMR spectra are collected in section 2. IR spectra were recorded on neat solids using a Perkin Elmer Spectrum GX IR (ATR) spectrometer. UV/Vis spectra were recorded using a Shimadzu UV-1800 double beam spectrophotometer in sealable quartz cells. Melting points were determined in sealed glass capillaries under argon and are uncorrected. Elemental analyses were performed by the Elemental Analysis Service at London Metropolitan University. C_{60} was purchased from Alfa Aesar (99 %) and stored in a glove box. The syntheses of $[\{(^{Ar}nacnac)Mg\}_2]$ **1** (**1a** Ar = Dip,[1] **1b** Ar = Dep,[2] **1c** Ar = Mes,[3] **1d** Ar = Xyl[4])[5] were performed according to literature procedures. Abbreviations: s = singlet, d = doublet, t = triplet, q = quartet, sept = septet, br = broad, vbr = very broad, m = multiplet.

1.2 Reactions of [{(^{Ar}nacnac)Mg}$_2$] 1 with C_{60}: preparations and formations of complexes 2-5

1.2.1 Reactions of [(^{Dip}nacnac)Mg}$_2$] 1a with C_{60}

N.B: formation of [{(^{Dip}nacnac)Mg}$_6C_{60}$] **2a** has so far never been observed, likely for steric reasons. Attempts at bulk isolation of [{(^{Dip}nacnac)Mg}$_2C_{60}$] **3a** and [{(^{Dip}nacnac)Mg}$_4C_{60}$] **4a** gave black-brown solutions characteristic of [{(^{Ar}nacnac)Mg}$_nC_{60}$] products, but only black-brown powders of questionable purity, as judged by ^{1}H NMR spectroscopy from redissolving samples, could be obtained from the mixtures.

[{(^{Dip}nacnac)Mg}$_2C_{60}$] 3a

C_{60} (8.50 mg, 11.8 µmol) and [{(^{Dip}nacnac)Mg}$_2$] **1a** (10.4 mg, 11.8 µmol, 1.00 equiv.) were added to an NMR tube with J. Young valve and benzene-*d$_6$* (0.5 mL) was added, giving a black-brown reaction mixture. The mixture was left standing at room temperature and the reaction was followed by ^{1}H NMR spectroscopy at regular intervals. Full consumption of [{(^{Dip}nacnac)Mg}$_2$] **1a** was observed after one day, giving [{(^{Dip}nacnac)Mg}$_4C_{60}$] **4a**. After further two days, **4a** was fully consumed and good conversion to [{(^{Dip}nacnac)Mg}$_2C_{60}$] **3a** was observed. Yield (solution): 81%. Crystals of solvates of **3a** suitable for X-ray crystallographic analysis were grown from concentrated solutions in benzene-*d$_6$*. ^{1}H NMR (400.1 Hz, benzene-*d$_6$*, 298 K) δ = 1.31 (d, J_{HH} = 6.8 Hz, 24H; CH(C*H*$_3$)$_2$), 1.66 (d, J_{HH} = 6.8 Hz, 24H; CH(C*H*$_3$)$_2$), 1.91 (s, 12H; NCC*H*$_3$), 3.53 (sept, J_{HH} = 6.8 Hz, 8H; C*H*(CH$_3$)$_2$), 5.21 (s, 2H; γ-C*H*), 6.91-7.18 (m, 12H; Ar-*H*); ^{13}C{^{1}H} NMR (125.7 MHz, benzene-*d$_6$*, 293 K) δ = 24.0 (CH(*C*H$_3$)$_2$), 24.5 (CH(*C*H$_3$)$_2$), 30.2 (*C*H(CH$_3$)$_2$), 96.1 (*C*H), 125.2 (Ar-*C*), 126.9 (Ar-*C*), 141.6 (Ar-*C*), 143.4 (Ar-*C*), 155.4 (*C*$_{60}$), 171.8 (N*C*CH$_3$).

[{(^{Dip}nacnac)Mg}$_4C_{60}$] 4a

C_{60} (10.5 mg, 14.6 µmol) and [{(^{Dip}nacnac)Mg}$_2$] **1a** (25.0 mg, 28.3 µmol, 1.94 equiv.) were added to an NMR tube with J. Young valve, benzene-*d$_6$* (0.5 mL) was added and the black-brown mixture was left standing at room temperature for five days and followed by ^{1}H NMR spectroscopy at regular intervals. Yield (solution): 42 % (Note: All **1a** was consumed, **4a** was the main species in solution and a dark precipitate was present.). After five days, single crystals of **4a** suitable for X-ray crystallographic analysis were grown by layering a concentrated benzene-*d$_6$* solution with *n*-hexane. ^{1}H NMR (400.1 MHz, benzene-*d$_6$*, 295 K) δ = 1.25 (d, J_{HH} = 6.7 Hz, 48H, CH(C*H*$_3$)$_2$), 1.52 (d, J_{HH} = 6.6 Hz, 48H, CH(C*H*$_3$)$_2$), 1.73 (s, 24H, NCC*H*$_3$), 3.26 (sept, J_{HH} = 6.9 Hz, 16H, C*H*(CH$_3$)$_2$), 4.95 (s, 4H, γ-C*H*), 6.90-7.29 (m, 24H, Ar-*H*). ^{13}C{^{1}H} NMR (100.6 MHz, benzene-*d$_6$*, 296 K) δ = 24.4 (CH(*C*H$_3$)$_2$), 24.6 (br., CH(*C*H$_3$)$_2$), 30.1 (*C*H(CH$_3$)$_2$), 95.6 (*C*H), 124.6 (Ar-*C*), 126.6 (Ar-*C*), 141.6 (Ar-*C*), 143.8 (Ar-*C*), 166.2 (br., *C*$_{60}$), 171.3 (N*C*CH$_3$).

1.2.2 Reactions of [(^{Dep}nacnac)Mg}$_2$] 1b with C_{60}

[{(^{Dep}nacnac)Mg}$_6C_{60}$] 2b

Toluene (30 mL) was added to a mixture of [{(^{Dep}nacnac)Mg}$_2$] **1b** (215.0 mg, 0.279 mmol, 3.24 equiv.) and C_{60} (62.0 mg, 0.086 mmol) at room temperature and the black-yellow solution was stirred at room temperature for 2 h. All volatiles were removed, and the black-brown oily residue was dissolved in *n*-hexane (20 mL) given a black-brown solution. Storing at 3°C for two days afforded black-brown crystals of [{(^{Dep}nacnac)Mg}$_6C_{60}$] **2b** suitable for X-ray crystallography. The filtrate was concentrated to *ca.* 10 mL and stored at -20°C to give a second crop of black-brown crystals. (N.B.: After drying under vacuum, these crops afforded a microanalysis that supports the composition of **2b**. It can not be ruled out that these crystalline crops are mixtures of [{(^{Dep}nacnac)Mg}$_6C_{60}$] **2b** and [{(^{Dep}nacnac)Mg}$_5C_{60}$] **5b**, see the NMR spectroscopy section. A percentage of **5b** would however lead to a worse fit for the microanalytical results.) After dissolution in benzene-d_6, ^{1}H NMR spectroscopy of both crops showed that a mixture of compounds had formed, including [{(^{Dep}nacnac)Mg}$_4C_{60}$] **4b** and a second product showing broad resonances for the β-diketiminate ligand tentatively assigned as [{(^{Dep}nacnac)Mg}$_5C_{60}$] **5b** (and further supported by other NMR spectroscopic data), though only trace quantities of [{(^{Dep}nacnac)Mg}$_2$] **1b** were present. Adding [{(^{Dep}nacnac)Mg}$_2$] **1b** to a sample of the isolated crystalline material in benzene-d_6 or toluene-d_8 afforded increasing resonances assigned to [{(^{Dep}nacnac)Mg}$_6C_{60}$] **2b** after several days; this process was faster at elevated temperatures. The formation of **2b** was supported by ^{13}C{^{1}H} NMR spectroscopy including the appropriate C_{60}^{6-} resonance. Obtained crystals were dried under vacuum. Yield = 133.0 mg (51%); Mp.: retains black-brown crystallinity up to 280°C (limit of melting point apparatus); ^{1}H NMR (400.1 MHz, benzene-d_6, 295 K) δ = 1.48 (t, J_{HH} = 7.6 Hz, 72H; CH_2CH_3), 1.72 (s, 36H; $NCCH_3$), 2.75 (dq, J_{HH} = 15.2, 7.6 Hz, 24H; CH_2CH_3), 2.80 (dq, J_{HH} = 15.2, 7.6 Hz, 24H; CH_2CH_3), 4.95 (s, 6H; γ-C*H*), 7.23-7.39 (m, 36H; Ar-*H*). ^{13}C{^{1}H} NMR (100.62 MHz, benzene-d_6, 298 K) δ = 15.0 ($NCCH_3$), 24.1 (CH_2CH_3), 25.5 (CH_2CH_3), 95.9 (*C*H), 125.6 (Ar-*C*), 126.7 (Ar-*C*), 137.7 (Ar-*C*), 146.0 (Ar-*C*), 153.2 (C_{60}), 170.0 ($NCCH_3$); ^{1}H NMR (400.1 MHz, toluene-d_8, 295 K): δ = 1.49 (t, J_{HH} = 7.5 Hz, 72H; CH_2CH_3), 1.74 (s, 36H; $NCCH_3$), 2.74 (dq, J_{HH} = 14.8, 7.4 Hz, 24H; CH_2CH_3), 2.80 (dq, J_{HH} = 14.8, 7.4 Hz, 24H; CH_2CH_3), 4.96 (s, 6H; γ-C*H*), 6.94-7.38 (m, 36H; Ar-*H*); ^{13}C{^{1}H} NMR (100.6 MHz, toluene-d_8, 295 K) δ = 15.3 ($NCCH_3$), 24.4 (CH_2CH_3), 25.9 (CH_2CH_3), 96.2 (*C*H), 126.0 (Ar-*C*), 127.1 (Ar-*C*), 137.6 (Ar-*C*), 146.3 (br.; Ar-*C*), 153.5 (br.; C_{60}), 170.3 (br.; $NCCH_3$); ^{1}H NMR (499.9 MHz, toluene-d_8, 193 K) δ = 1.49 (vbr. t, 72H; CH_2CH_3), 1.69 (br. s, 36H; $NCCH_3$), 2.76 (vbr. dq, 24H; CH_2CH_3), 2.85 (vbr. dq, 24H; CH_2CH_3), 4.88 (br. s, 6H; γ-C*H*), 7.11-7.50 (m, 36H; Ar-*H*); ^{13}C{^{1}H} NMR (125.7 MHz, toluene-d_8, 192 K) δ = 15.3 ($NCCH_3$), 24.9

(CH_2CH_3), 25.6 (CH_2CH_3), 96.5 (*C*H), 127.1 (Ar-*C*), 137.1 (Ar-*C*), 146.6 (br.; Ar-*C*), 153.0 (br.; C_{60}), 169.9 (br.; N*C*CH_3); IR (nujol), ν~/cm^{-1}: 2963 (s), 2930 (m), 2872 (m), 1661 (m), 1620 (s), 1591 (w), 1549 (s), 1489 (w), 1443 (m), 1362 (s), 1339 (w), 1265 (m), 1177 (s), 1105 (m), 1059 (w), 1022 (m), 932 (w), 854 (m), 795 (m), 760 (s), 700 (w), 570 (s), 523 (m); elemental analysis: calculated for $C_{210}H_{198}Mg_6N_{12}$: C, 83.09; H, 6.57; N, 5.54%; found: C, 82.83; H, 6.69; N 5.38%.

[{(^{Dep}nacnac)Mg}$_2$C$_{60}$] 3b

Toluene (30 mL) was added to a mixture of [{(^{Dep}nacnac)Mg}$_2$] **1b** (59.0 mg, 0.077 mmol, 1.10 equiv.) and C_{60} (50.0 mg, 0.069 mmol) at room temperature and the black-brown solution was stirred at room temperature for 2 h. The solution was concentrated to 7 mL, layered with *n*-hexane (20 mL) and was allowed to stand for two days forming a brown polycrystalline precipitate. Black-brown crystals of [{(^{Dep}nacnac)Mg}$_6$C$_{60}$] **3b** suitable for X-ray crystallography were grown by slowly cooling a concentrated benzene-d_6 solution from 50°C to room temperature. Yield (polycrystalline precipitate) = 47.0 mg (45%). ^{1}H NMR (499.9 MHz, benzene-d_6, 298 K) δ = 1.45 (t, J_{HH} = 7.5 Hz, 24H; CH_2CH_3), 1.88 (s, 12H; NCC*H*$_3$), 2.87 (dq, J_{HH} = 15.0, 7.5 Hz, 8H; C*H*$_2$CH$_3$), 3.10 (dq, J_{HH} = 15.0, 7.5 Hz, 8H; C*H*$_2$CH$_3$), 5.22 (s, 2H; γ-C*H*), 6.67-7.13 (m, 12H; Ar-*H*); ^{13}C{^{1}H} NMR (125.7 MHz, benzene-d_6, 295 K) δ = 14.8 (NC*C*H$_3$), 23.4 (CH$_2$*C*H$_3$), 26.0 (*C*H$_2$CH$_3$), 96.4 (*C*H), 126.6 (Ar-*C*), 127.5 (Ar-*C*), 129.3 (Ar-*C*), 137.3 (Ar-*C*), 144.8 (Ar-*C*), 156.5 (C_{60}), 171.5 (N*C*CH$_3$); ^{1}H NMR (499.9 MHz, toluene-d$_8$, 295 K) δ = 1.45 (t, J_{HH} = 7.7 Hz, 24H; CH$_2$C*H*$_3$), 1.89 (s, 12H; NCC*H*$_3$), 2.86 (dq, J_{HH} = 15.0, 7.6 Hz, 8H; C*H*$_2$CH$_3$), 3.08 (dq, J_{HH} = 15.0, 7.6 Hz, 8H; C*H*$_2$CH$_3$), 5.22 (s, 2H; γ-C*H*), 6.88-6.96 (m, 4H; Ar-*H*), 6.99 (s, 4H; Ar-*H*), 7.10 (m, 4H; Ar-*H*); ^{13}C{^{1}H} NMR (125.7 MHz, toluene-d_8, 295 K) δ = 15.1 (NC*C*H$_3$), 23.8 (CH$_2$*C*H$_3$), 26.4 (*C*H$_2$CH$_3$), 96.8 (γ-*C*H), 127.0 (Ar-*C*), 127.8 (Ar-*C*), 129.6 (Ar-*C*), 137.7 (Ar-*C*), 145.2 (Ar-*C*), 156.6 (br.; C_{60}), 171.9 (N*C*CH$_3$); ^{1}H NMR (499.9 MHz, toluene-d$_8$, 193 K) δ = 1.39 (br. t, J_{HH} = 7.3 Hz, 24H; CH$_2$C*H*$_3$), 1.78 (br. s, 12H; NCC*H*$_3$), 2.64 (vbr. dq, J_{HH} = 15.7, 8.0 Hz, 8H; C*H*$_2$CH$_3$), 2.96 (vbr. dq, J_{HH} = 14.8, 7.5 Hz, 8H; C*H*$_2$CH$_3$), 5.17 (br. s, 2H; γ-C*H*), 6.94 (s, 6H; Ar-*H*), 7.17 (s, 6H; Ar-*H*); ^{13}C{^{1}H} NMR (125.7 MHz, toluene-d_8, 193 K) δ = 14.9 (NC*C*H$_3$), 24.1 (CH$_2$*C*H$_3$), 25.6 (*C*H$_2$CH$_3$), 96.9 (br.; *C*H), 126.4 (Ar-*C*), 127.1 (Ar-*C*), 129.6 (Ar-*C*), 137.0 (Ar-*C*), 145.2 (Ar-*C*), 156.1 (C_{60}), 171.3 (N*C*CH$_3$); Note; one Ar-*C* resonance is believed hidden under the strong solvent resonnces; IR (nujol), ν~/cm^{-1}: 3657 (br. w), 3055 (m), 2870 (w), 2839 (w), 1659 (m), 1628 (m), 1551 (m), 1512 (m), 1443 (s), 1396 (m), 1366 (s), 1273 (m), 1204 (w), 1173 (s), 1103 (w), 1018 (br. m), 964 (w), 864 (m), 795 (m), 764 (s), 725 (m), 694 (m), 571 (s), 525 (s); Elemental analysis afforded acceptable values for H and N, but values 5% too low for C. This carbon deficiency was attributed to either incomplete combustion or impurities in the sample.

[{(^{Dep}nacnac)Mg}$_4$C$_{60}$] 4b

C_{60} (8.3 mg, 12.9 µmol) and [{(^{Dep}nacnac)Mg}$_2$] **1b** (10.0 mg, 13.0 µmol, 1.00 equiv.) were added to an NMR tube with J. Young valve, and benzene-d_6 (0.5 mL) was added resulting in a black-brown reaction mixture. The mixture was left standing at room temperature and followed by ^{1}H NMR spectroscopy at regular intervals. Complete consumption of **1b** and good conversion to [{(^{Dep}nacnac)Mg}$_4$C$_{60}$] **4b** was observed after 52 h, plus the formation of a small quantity of a black-brown precipitate. Attempts at bulk isolation of [{(^{Dep}nacnac)Mg}$_4$C$_{60}$] **4b** gave black-brown solutions characteristic of [{(^{Ar}nacnac)Mg}$_n$C$_{60}$] products, but only black-brown powder of unclear purity, as judged by ^{1}H NMR spectroscopy upon redissolving, could be obtained from the mixtures. Yield (solution): 71%. The assignment of [{(^{Dep}nacnac)Mg}$_4$C$_{60}$] **4b** was aided by the broad fulleride resonance in the ^{13}C{^{1}H} NMR spectrum. Quantities of complex [{(^{Dep}nacnac)Mg}$_4$C$_{60}$] **4b** were also generated when solutions containing [{(^{Dep}nacnac)Mg}$_6$C$_{60}$] **2b** (containing increasing quantities of **4b** and **5b**) were briefly exposed to air. ^{1}H NMR (499.9 MHz, benzene-d_6, 295 K) δ = 1.37 (t, J_{HH} = 7.5 Hz, 48H; CH$_2$C*H*$_3$), 1.67 (s, 24H; NCC*H*$_3$), 2.64 (dq, J_{HH} = 14.9, 7.4 Hz, 16H; C*H*$_2$CH$_3$), 2.85 (dq, J_{HH} = 14.9, 7.4 Hz, 16H; C*H*$_2$CH$_3$), 4.92 (s, 4H; γ-C*H*), 7.01-7.14 (m, 24H; Ar-*H*); ^{13}C{^{1}H} NMR (125.7 MHz, benzene-d_6, 295 K) δ = 14.7 (NC*C*H$_3$), 23.6 (CH$_2$*C*H$_3$), 25.7 (*C*H$_2$CH$_3$), 95.5 (*C*H), 126.1 (Ar-*C*), 126.9 (Ar-*C*), 137.2 (Ar-*C*), 144.9 (Ar-*C*), 167.4 (br.; *C*$_{60}$), 170.7 (N*C*CH$_3$); ^{1}H NMR (400.1 MHz, toluene-d$_8$, 295 K) δ = 1.36 (t, J_{HH} = 7.4 Hz, 48H; CH$_2$C*H*$_3$), 1.67 (s, 24H; NCC*H*$_3$), 2.62 (dq, J_{HH} = 15.0, 7.6 Hz, 16H; C*H*$_2$CH$_3$), 2.83 (dq, J_{HH} = 15.0, 7.6 Hz, 16H; C*H*$_2$CH$_3$), 4.91 (s, 4H; γ-C*H*), 7.00-7.08 (m, 24H; Ar-*H*); ^{1}H NMR (499.9 MHz, toluene-d$_8$, 193 K) δ = 1.46 (br. t, J_{HH} = 7.6 Hz, 48H; CH$_2$C*H*$_3$), 1.64 (br. s, 24H; NCC*H*$_3$), 2.59 (vbr. dq, J_{HH} = 15.0, 8.0 Hz, 16H; C*H*$_2$CH$_3$), 2.91 (vbr. dq, J_{HH} = 15.0, 8.0 Hz, 16H; C*H*$_2$CH$_3$), 4.86 (br. s, 4H; γ-C*H*), 7.11-7.21 (m, 24H; Ar-*H*); ^{13}C{^{1}H} NMR (125.7 MHz, toluene-d_8, 193 K) δ = 14.9 (NC*C*H$_3$), 24.1 (CH$_2$*C*H$_3$), 25.9 (*C*H$_2$CH$_3$), 95.7 (*C*H), 125.2 (Ar-*C*), 126.2 (Ar-*C*), 127.0 (Ar-*C*), 137.1 (Ar-*C*), 145.1 (Ar-*C*), 167.3 (br.; *C*$_{60}$), 170.6 (N*C*CH$_3$).

[{(^{Dep}nacnac)Mg}$_5$C$_{60}$] 5b

At high concentrations of [{(^{Dep}nacnac)Mg}$_2$] relative to C$_{60}$ (approximately Mg 5:1 C$_{60}$), [{(^{Dep}nacnac)Mg}$_5$C$_{60}$] **5b** was observable by ^{1}H NMR spectroscopy, and shows broad resonances. Attempts to isolate pure crystalline material were unsuccessful so far, and a mixture of products including some **2b** and **4b** was typically observed by NMR spectroscopy that changed in composition over time. Various ^{1}H NMR and ^{13}C{^{1}H} NMR spectra of different reaction mixtures, including the Evan's method, and variable temperature ^{1}H NMR spectroscopy in benzene-d_6 or toluene-d_8 aided in the assignment of resonances to one compound with the tentative formulation as [{(^{Dep}nacnac)Mg}$_5$C$_{60}$] **5b**. ^{1}H NMR (499.9 MHz, benzene-d_6, 343 K) δ = 1.61 (vbr., 60H;

CH_2CH_3), 1.79 (s, 30H; NCCH_3), 2.98 (vbr., 40H; CH_2CH$_3$), 4.82 (br. s, 5H; γ-C*H*), 6.90-7.15 (br m, 30H; Ar-*H*); ^{1}H NMR (499.9 MHz, toluene-d_8, 373 K): δ = 1.55 (vbr., 60H; CH$_2$CH_3), 1.76 (s, 30H; NCCH_3), 2.92 (vbr., 40H; CH_2CH$_3$), 4.80 (br. s, 5H; γ-C*H*), 6.84-7.18 (br m, 30H; Ar-*H*); ^{13}C{^{1}H} NMR (125.7 MHz, benzene-d_6, 295 K) tentative assignment on broad resonances only, δ approximately: 21 (vbr, NC*C*H$_3$), 26 (CH$_2$*C*H$_3$), 32 (*C*H$_2$CH$_3$), 97 (*C*H), 126 (Ar-*C*), 129 (Ar-*C*), 140 (Ar-*C*), 145 (Ar-*C*), 170 (vbr., N*C*CH$_3$), 174 (br, C_{60}). Several spectra were recorded to support tentative assignment, including those with very short relaxation delay (0.1 s). A broad resonance at around 170 ppm was assigned to the CN environment, and the broad resonance at δ 174 ppm was assigned to the fulleride (C_{60}^{5-}) fragment, although this cannot be certain.

1.2.3 Reactions of [(^{Mes}nacnac)Mg}$_2$] 1c with C$_{60}$

[{(^{Mes}nacnac)Mg}$_6$C$_{60}$] 2c

Small scale: A mixture of C_{60} (10.2 mg, 14.0 µmol) and [{(^{Mes}nacnac)Mg}$_2$] **1c** (30.0 mg, 42.0 µmol, 3.00 equiv.) were added to an NMR tube with J. Young valve and dissolved in benzene-d_6 (0.6 mL), giving a black-brown mixture. The ^{1}H NMR spectrum shows conversion to a solution of [{(^{Mes}nacnac)Mg}$_6$C$_{60}$] **2c** after two days at room temperature (57 % yield dissolved in solution). Black-brown crystals of solvates of **2c** suitable for X-ray crystallographic analysis were grown from a concentrated benzene-d_6 solution. Reaction times could be decreased significantly (to 3 h) when the reagents were finely ground together to a homogeneous mixture prior to solvent addition. N.B.: We found no evidence that simply grinding the reagents in the solid state (in a glove box at room temperature) forms a significant amount of magnesium fulleride complexes as judged from ^{1}H NMR experiments acquired immediately after mixing, although and as expected, significant conversion was observed in the solution state. Instead, we believe that the dominating effect is that poorly soluble C_{60} is more readily dissolved in finer form and can react faster.

Preparative scale: A solution of [{(^{Mes}nacnac)Mg}$_2$] **1c** (215.0 mg, 0.300 mmol, 3.23 equiv.) in toluene (15 mL) was added in three aliquots (3 × 5 mL) to a purple solution of C_{60} (67.0 mg, 0.093 mmol) in toluene (10 mL). Between each addition, the black-brown mixture was left to stir at room temperature for 1 h, and then stirred for a further 3 h. All volatiles were removed under reduced pressure and *n*-hexane (10 mL) was added. Storing the solution at -40°C afforded a crop of black-brown crystals suitable for X-ray crystallography, which were subsequently isolated and dried under vacuum. Yield = 131.0 mg (49%); ^{1}H NMR (400.1 MHz, benzene-d_6, 294 K) δ = 1.71 (s, 36H; NCCH_3), 2.32 (s, 72H; *o*-CH_3), 2.54 (s, 36H; *p*-CH_3), 4.97 (s, 6H; γ-C*H*), 7.01 (s, 24H; Ar-*H*). ^{13}C{^{1}H} NMR (125.7 MHz, benzene-d_6, 293 K) δ = 19.7 (NC*C*H$_3$), 21.7 (*p*-*C*H$_3$), 23.6 (*o*-*C*H$_3$), 95.6 (*C*H), 130.0 (Ar-*C*), 131.7 (Ar-*C*), 133.9 (Ar-*C*), 144.3 (Ar-*C*), 152.9 (C_{60}), 169.8 (N*C*CH$_3$);

IR (nujol), v~/cm^{-1}: 2913 (br. w), 2853 (w), 1541 (br. m), 1518 (s), 1476 (w), 1450 (m), 1396 (s), 1375 (br. s), 1258 (s), 1196 (s), 1146 (s), 1013 (br. m), 957 (w), 924 (w), 854 (s), 743 (w), 652 (w), 567 (s), 507 (s); M.p.: no visible decomposition up to 280°C (limit), compound remains black-brown crystalline throughout heating; UV/Vis (toluene) λ_{max} [nm] (ε in mol^{-1}dm^{3}cm^{-1}): 428 (~12,800); elemental analysis: calculated for $C_{198}H_{174}Mg_6N_{12}$: C, 82.94; H, 6.12; N, 5.86%; found: C, 82.77; H, 6.29; N 5.98%.

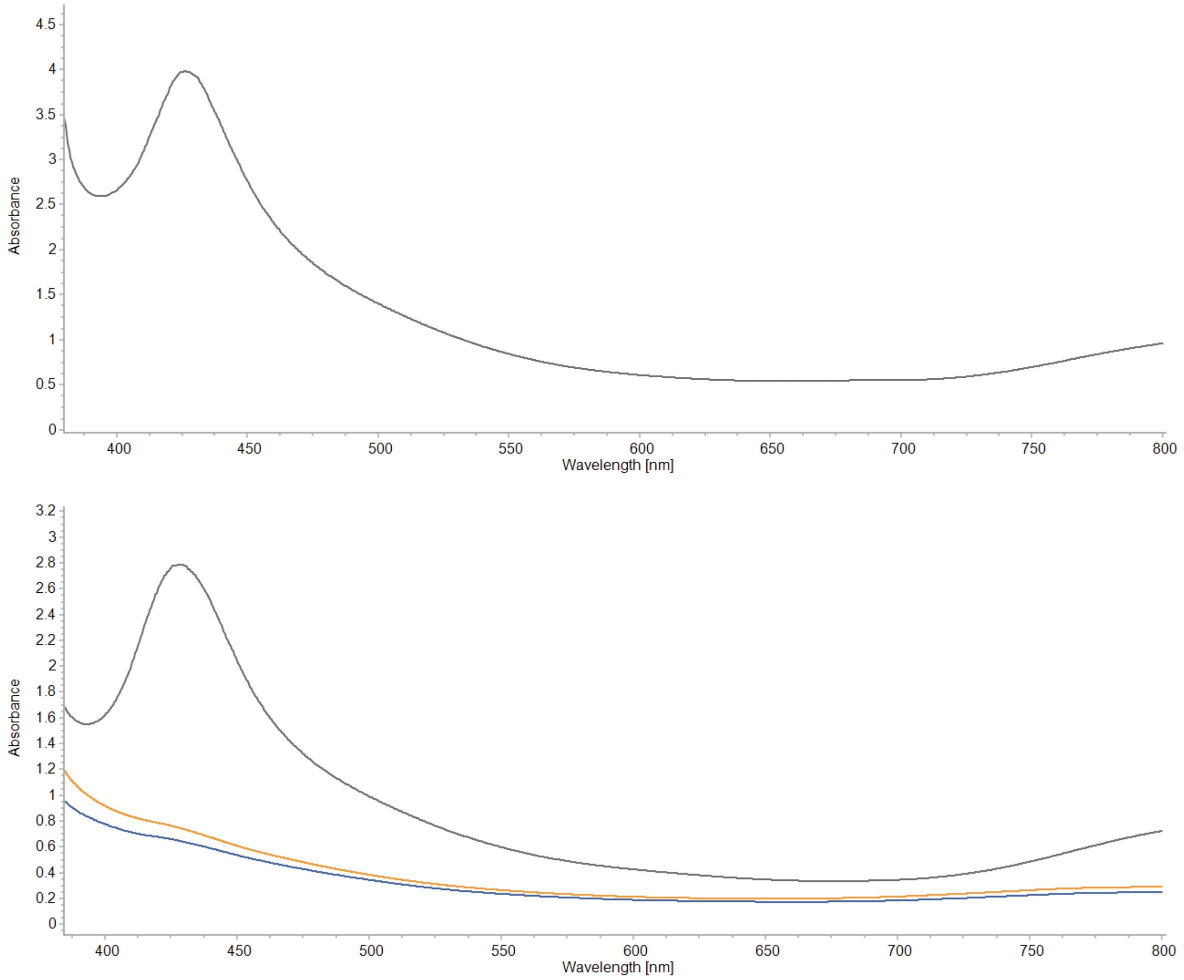

Figure S1: UV spectra of [{(^{Mes}Nacanc)Mg}$_6$C$_{60}$] **2c** at various concentrations in toluene (top) and *n*-hexane (bottom). The absorbance maximum is at *ca.* 428 nm in both solvents. 800 nm was the limit of the experiment. For comparison, neutral C_{60} shows a maximum at 328 nm under similar conditions.[6]

[{(^{Mes}nacnac)Mg}$_2$] and C_{60}

C_{60} (8.0 mg, 11.0 µmol) and [{(^{Mes}nacnac)Mg}$_2$] **1c** (8.0 mg, 11.2 µmol, 1.02 equiv.) were added to an NMR tube with J. Young valve and benzene-*d$_6$* (0.5 mL) was added giving a black-brown reaction mixture that was followed by NMR spectroscopy at regular intervals. After three days at room temperature the complete consumption of **1c** and good conversion to [{(^{Mes}nacnac)Mg}$_6$C$_{60}$] **2c** was observed, followed by further slow conversion to [{(^{Mes}nacnac)Mg}$_4$C$_{60}$] **4c** and subsequently, after twelve days, to [{(^{Mes}nacnac)Mg}$_2$C$_{60}$] **3c**. Attempts to isolate pure crystalline **3c** and **4c** or large crystals were unsuccessful so far and the compounds were assigned using NMR spectroscopy.

NMR spectroscopic data for [{(^{Mes}nacnac)Mg}$_2$C$_{60}$] 3c

Attempts at bulk isolation gave black-brown solutions characteristic of [{(^{Ar}nacnac)Mg}$_n$C$_{60}$] products, but only impure black-brown powder could be obtained from the mixtures. Yield (solution): 18 % (plus precipitated material, which is not considered). ^{1}H NMR (499.9 MHz, benzene-*d*$_6$, 295 K) δ = 1.90 (s, 12H; NCC*H*$_3$), 1.97 (s, 12H; *p*-C*H*$_3$), 2.47 (s, 24H; *o*-C*H*$_3$), 5.25 (s, br, 2H; γ-C*H*), 6.75 (s, 8H; Ar-*H*); ^{13}C{^{1}H} NMR (125.7 MHz, benzene-*d*$_6$, 295 K) δ = 19.2 (NC*C*H$_3$), 20.9 (*p*-*C*H$_3$), 23.2 (*o*-*C*H$_3$), 96.5 (C*H*), 130.4 (Ar-*C*), 131.6 (Ar-*C*), 135.5 (Ar-*C*), 143.4 (Ar-*C*), 156.3 (br., *C*$_{60}$), 171.7 (N*C*CH$_3$).

NMR spectroscopic data for [{(^{Mes}nacnac)Mg}$_4$C$_{60}$] 4c

Attempts at bulk isolation gave black-brown solutions characteristic of [{(^{Ar}nacnac)Mg}$_n$C$_{60}$] products, but only impure black-brown powder could be obtained from the mixtures. Yield (solution): 49 % (plus precipitated material, which is not considered). ^{1}H NMR (499.9 MHz, benzene-*d*$_6$, 295 K) δ = 1.68 (s, 24H; NCC*H*$_3$), 2.18 (s, 24H; *p*-C*H*$_3$), 2.29 (s, 48H; *o*-C*H*$_3$), 4.93 (s, 4H; γ-C*H*), 6.84 (s, 16H; Ar-*H*); ^{13}C{^{1}H} NMR (125.7 MHz, benzene-*d*$_6$, 295 K) δ = 19.1 (NC*C*H$_3$), 21.2 (*p*-*C*H$_3$), 23.2 (*o*-*C*H$_3$), 95.5 (C*H*), 130.1 (Ar-*C*), 131.6 (Ar-*C*), 134.7 (Ar-*C*), 143.4 (Ar-*C*), 166.2 (br., *C*$_{60}$), 170.7 (N*C*CH$_3$).

1.2.4 Reactions of [(^{Xyl}nacnac)Mg}$_2$] 1d with C_{60}

[{(^{Xyl}nacnac)Mg}$_6$C$_{60}$] 2d

A mixture of C_{60} (8.0 mg, 11.1 µmol) and [{(^{Xyl}nacnac)Mg}$_2$] **1d** (21.9 mg, 33.2 µmol, 2.99 equiv.) were added to an NMR tube with J. Young valve, benzene-*d$_6$* (0.6 mL) was added resulting in a black-brown mixture. The ^{1}H NMR spectrum shows good conversion to [{(^{Xyl}nacnac)Mg}$_6$C$_{60}$] **2d** after one day at room temperature. Black-brown crystals of **2d** suitable for X-ray crystallographic

analysis were grown from a concentrated benzene-d_6 solution. Reaction times were decreased significantly when grinding the reagents to a homogeneous mixture prior to reaction. Yield (solution): 65 % (plus precipitated material, which is not considered). ^{1}H NMR (499.9 MHz, benzene-d_6, 295 K) δ = 1.69 (s, 36H; NCCH_3), 2.31 (s, 72H; *o*-CH_3), 4.95 (s, 6H; γ-C*H*), 7.19 (s, 36H; Ar-*H*); ^{13}C{^{1}H} NMR (125.7 MHz, benzene-d_6, 295 K) δ = 19.9 (NCCH$_3$), 23.6 (*o*-CH$_3$), 95.7 (*C*H), 125.3 (Ar-*C*), 129.2 (Ar-*C*), 132.3 (Ar-*C*), 146.8 (Ar-*C*), 152.8 (C_{60}), 169.6 (N*C*CH$_3$).

[{(^{Xyl}nacnac)Mg}$_2$] and C_{60}

C_{60} (8.7 mg, 12.1 µmol) and [{(^{Xyl}nacnac)Mg}$_2$] **1d** (8.0 mg, 12.1 µmol, 1.01 equiv.) were added to an NMR tube with J. Young valve, and benzene-d_6 (0.6 mL) was added resulting in a black-brown reaction mixture. Consumption of starting material **1d** was observed alongside the formation of [{(^{Xyl}nacnac)Mg}$_6$C$_{60}$] **2d**, with full consumption of **1d** observed after one day at room temperature. The steady formation of [{(^{Xyl}nacnac)Mg}$_4$C$_{60}$] **4d** was observed over time and after five days it was the only observable product by ^{1}H NMR spectroscopy. A black-brown solid precipitated, considerably hindering conversion to [{(^{Xyl}nacnac)Mg}$_2$C$_{60}$] **3d** which was complete after approximately eighteen days. Attempts were made to isolate pure crystalline **3d** and **4d** but were unsuccessful so far. Instead the compounds were characterised by NMR spectroscopy.

[{(^{Xyl}nacnac)Mg}$_2$C$_{60}$] 3d

Attempts at bulk isolation gave black-brown solutions characteristic of [{(^{Ar}nacnac)Mg}$_n$C$_{60}$] products, but only impure black-brown powder could be obtained from the mixtures. Yield (solution): 76 % (plus precipitated material, which is not considered) ^{1}H NMR (499.9 MHz, benzene-d_6, 295 K) δ = 1.80 (s, 12H; NCCH_3), 2.45 (s, 24H; *o*-CH_3), 5.18 (br. s, 2H; γ-C*H*), 6.80-6.83 (m, 4H; *p*-Ar-*H*), 6.93-6.94 (m, 8H; *m*-Ar-*H*); ^{13}C{^{1}H} NMR (125.7 MHz, benzene-d_6, 295 K) δ = 19.3 (br.; NCCH$_3$), 23.3 (*o*-CH$_3$), 96.5 (*C*H), 126.2 (Ar-*C*), 129.7 (Ar-*C*), 132.0 (Ar-*C*), 146.1 (Ar-*C*), 156.2 (br.; C_{60}), 171.6 (N*C*CH$_3$).

[{(^{Xyl}nacnac)Mg}$_4$C$_{60}$] 4d

Attempts at bulk isolation gave black-brown solutions characteristic of [{(^{Ar}nacnac)Mg}$_n$C$_{60}$] products, but only impure black-brown powder could be obtained from the mixtures. Yield (solution): 76 % (plus precipitated material, which is not considered). ^{1}H NMR (499.9 MHz, benzene-d_6, 295 K) δ = 1.61 (s, 24H; NCCH_3), 2.28 (s, 48H; *o*-CH_3), 4.90 (s, 4H; γ-C*H*), 6.87-7.00 (m, 24H; Ar-*H*); ^{13}C{^{1}H} NMR (125.7 MHz, benzene-d_6, 295 K) δ = 19.1 (NCCH$_3$), 23.3 (*o*-CH$_3$), 95.6 (*C*H), 125.7 (Ar-*C*), 129.3 (Ar-*C*), 132.0 (Ar-*C*), 146.0 (Ar-*C*), 166.3 (br.; C_{60}), 170.6 (N*C*CH$_3$).

1.3 Titration experiments of [{(^{Ar}nacnac)Mg}$_2$] 1 and C_{60}

1.3.1 General Procedure

[{(^{Ar}Nacnac)Mg}$_2$] **1** (0.5 equiv.) was added to an NMR tube with J. Young valve, dissolved in C_6D_6 (0.6 mL) and left standing at room temperature. C_{60} (1 equiv.) was added and the reaction was monitored by ^{1}H NMR spectroscopy over time; where possible until a near-equilibrium of resonances was reached. Further additions of 0.5 equivalents of [{(^{Ar}Nacnac)Mg}$_2$] **1** were repeated and monitored until a noticeable excess of **1** was present in solution (occuring after 3.5-4.0 equivalents had been administered to the NMR tube). Selected spectra for these experiments are collected in section 2.3.

1.3.2 Titration experiment of [{(^{Dip}nacnac)Mg}$_2$] 1a and C_{60}

Following the general procedure portions of [{(^{Dip}Nacnac)Mg}$_2$] **1a** (5.2 mg, 5.9 μmol, 0.5 equiv.) were reacted with C_{60} (8.5 mg, 11.8 μmol) and monitored over time using ^{1}H NMR spectroscopy.

First addition: Rapid formation of [{(^{Dip}nacnac)Mg}$_4C_{60}$] **4a** was observed, followed by gradual consumption (over two days) to give [{(^{Dip}nacnac)Mg}$_2C_{60}$] **3a** as main product in solution. Large amount of brown-black precipitate present.

Second addition: A mixture of unreacted [{(^{Dip}Nacnac)Mg}$_2$] **1a**, [{(^{Dip}nacnac)Mg}$_4C_{60}$] **4a** and [{(^{Dip}nacnac)Mg}$_2C_{60}$] **3a** was observed initially. **1a** was consumed after 18 hours to give **4a** as the main product, which slowly reacted with C_{60} to give **3a** after eight days.

Third addition: A mixture of unreacted **1a**, **4a** and **3a** seen initially. **1a** was consumed to give **4a** in considerable concentration. After eight days an equilibrium was reached whereby there was an approximate 1:1 ratio of **4a** and **3a** in solution.

Fourth addition: A mixture of unreacted **1a**, **4a** and **3a** was seen initially. Over time, **1a** and **3a** were consumed until only **4a** was present.

Fifth addition: No reactivity of **1a** that was added, with no change in concentration of **4a**, as observed over time.

Sixth and seventh additions: Similar to the fifth addition, any further addition of [{(^{Dip}Nacnac)Mg}$_2$] **1a** remained largely unreacted.

1.3.3 Titration experiment of [{(^{Dep}nacnac)Mg}$_2$] 1b and C_{60}

Following the general procedure portions of [{(^{Dep}Nacnac)Mg}$_2$] **1b** (8.0 mg, 10.4 μmol, 0.5 equiv.) were reacted with C_{60} (15.0 mg, 20.8 μmol) and monitored over time using ^{1}H NMR spectroscopy. Some tetramethylsilane (TMS) and a TMS/C_6D_6 glass insert were added; an ^{1}H NMR spectrum was taken as an initial reference (Evan's method to determine a relative shift as an indication of solution

magnetic susceptibility for the mixture). The observed relative shifts (red Hertz values) are given as a guide only and do not reflect the accuracy of the method.

First addition: Complete consumption of **1b** is observed after 19 h; [{(^{Dep}nacnac)Mg}$_4$C$_{60}$] **4b** was one of the main products plus a small amount of decomposition to the homoleptic complex [(^{Dep}Nacnac)$_2$Mg]. Further reaction of **4b** with C$_{60}$, over the course of two days, led to the formation of [{(^{Dep}nacnac)Mg}$_2$C$_{60}$] **3b**. Following this, multiple other β-diketiminate species started to form in low concentrations, which were difficult to characterise.

Second addition: After addition, rapid formation of mainly **4b** was observed. After 50 h at room temperature **3b** was present as the major product, with no significant further change in concentration over time (nine days).

Third addition: Immediately a complex mixture of resonances is observed. After three days, **3b** was observed as the major product, with no significant change in composition over time (five days).

Fourth addition: [{(^{Dep}nacnac)Mg}$_4$C$_{60}$] **4b** was the dominant species as part of a mixture of products, with resonances for **3b** growing very slowly in concentration throughout and [{(^{Dep}nacnac)Mg}$_6$C$_{60}$] **2b** also present in very low concentrations.

Fifth addition: [{(^{Dep}nacnac)Mg}$_4$C$_{60}$] **4b** was the major product throughout, with only small quantities of other compounds present in solution.

Sixth addition: [{(^{Dep}nacnac)Mg}$_4$C$_{60}$] **4b** remained as the main species in solution throughout with some [{(^{Dep}nacnac)Mg}$_2$] **1b** converting over time to small quantities of [{(^{Dep}nacnac)Mg}$_5$C$_{60}$] **5b** and [{(^{Dep}nacnac)Mg}$_6$C$_{60}$] **2b**.

Seventh addition: Initially, [{(^{Dep}nacnac)Mg}$_4$C$_{60}$] **4b** and [{(^{Dep}nacnac)Mg}$_2$] **1b** were present in approximate similar concentrations and decreased over time forming more [{(^{Dep}nacnac)Mg}$_6$C$_{60}$] **2b** and [{(^{Dep}nacnac)Mg}$_5$C$_{60}$] **5b**.

Eight addition: Further formation of [{(^{Dep}nacnac)Mg}$_6$C$_{60}$] **2b** was observed over time at the expense of **1b** and **5b**.

The approximate chemical shift (red Hertz values) as a relative indication for the magnetic susceptibility from paramagnetic compounds in solution (Evan's method) largely corresponds to concentrations of putative [{(^{Dep}nacnac)Mg}$_5$C$_{60}$] **5b**, which would have to be a paramagnetic species, and shows broad resonances. Values for the solution shift (related to magnetic susceptibility) later diminish again when more **5b** was consumed and converted to more diamagnetic [{(^{Dep}nacnac)Mg}$_6$C$_{60}$] **2b**.

1.3.4 Titration experiment of [{(^{Mes}nacnac)Mg}$_2$] 1c and C_{60}

Following the general procedure portions of [{(^{Mes}Nacnac)Mg}$_2$] **1c** (7.0 mg, 9.8 µmol, 0.5 equiv.) were reacted with C_{60} (14.1 mg, 19.6 µmol) and monitored over time using ^{1}H NMR spectroscopy. Some tetramethylsilane (TMS) and a TMS/C_6D_6 glass insert were added; an ^{1}H NMR spectrum was taken as an initial reference (Evan's method to determine a relative shift as an indication of solution magnetic susceptibility for the mixture). The observed relative shifts (red Hertz values) are given as a guide only and do not reflect the accuracy of the method.

First addition: formation of [{(^{Mes}nacnac)Mg}$_6$C$_{60}$] **2c** was observed instantly. [{(^{Mes}nacnac)Mg}$_4$C$_{60}$] **4c** was the major product after one day and gradual consumption occurred over time to give [{(^{Mes}nacnac)Mg}$_2$C$_{60}$] **3c** (eleven days).
Second addition: Immediately after addition, [{(^{Mes}nacnac)Mg}$_6$C$_{60}$] **2c** and [{(^{Mes}nacnac)Mg}$_4$C$_{60}$] **4c** were present in an approximate 2:1 ratio, respectively. After longer reaction times, only **4c** was present, alongside a black precipitate in the NMR tube.
Third, fourth and fifth addition: [{(^{Mes}nacnac)Mg}$_6$C$_{60}$] **2c** and [{(^{Mes}nacnac)Mg}$_4$C$_{60}$] **4c** dominate and their concentrations change over time.
Sixth addition: Largely [{(^{Mes}nacnac)Mg}$_6$C$_{60}$] **2c** was present.
Seventh addition: Largely [{(^{Mes}nacnac)Mg}$_6$C$_{60}$] **2c** present and unreacted [{(^{Mes}Nacnac)Mg}$_2$] **1c**.

The chemical shift (red Hertz values, Evan's method) is consistently low when low numbers of equivalents of **1c** were added and various concentrations **2c**, **3c** and **4c** were present. The value somewhat rises when larger quantities of **1c** were added though no resonances for an obvious paramagnetic species have been identified so far.

1.4 Reaction of [{(^{Mes}nacnac)Mg}$_6$C$_{60}$] 2c with [(^{Xyl}nacnac)Li]

1.4.1 Preparation of [(^{Xyl}nacnac)Li]

A hexane solution of *n*-butyllithium (1.6 M, 6.73 mL, 10.77 mmol, 1.10 equiv.) was slowly added to a cooled (0°C) solution of ^{Xyl}nacnacH (3.00 g, 9.79 mmol) in *n*-hexane (15 mL). A white precipitate started to form during the addition. The mixture was stirred for one hour, the product [(^{Xyl}nacnac)Li] was allowed to settle, filtered and dried under vacuum. Concentrating the filtrate to *ca.* 4 mL and storing at -40°C for one day afforded a second crop of white crystalline [(^{Xyl}nacnac)Li]. Yield: 2.90 g (95 %); ^{1}H NMR (499.9 MHz, benzene-d_6, 295 K) δ = 1.65 (s, 6H; NCCH_3), 2.03 (s, 12H; *o*-CH_3), 4.77 (s, 1H; CH), 6.97 (t, J = 7.4 Hz, 2H; *p*-Ar-H), 7.10 (d, J = 7.4 Hz, 4H; *m*-Ar-H); ^{7}Li NMR (155.5 MHz, benzene-d_6, 295 K) δ = -1.13 (s); ^{13}C{^{1}H} NMR (125.7 MHz, benzene-d_6, 295 K) δ = 18.5 (NCCH$_3$), 22.5 (*o*-CH$_3$), 121.9 (Ar-C), 130.4 (Ar-C), 152.1 (Ar-

C), 162.7 (N*C*CH$_3$); IR (nujol), ṽ/cm^{-1}: 3017 (w), 2943 (w), 2913 (w), 1622 (s), 1549 (s), 1516 (m), 1435 (m), 1396 (br. s), 1373 (s), 1275 (s), 1223 (w), 1180 (s), 1086 (m), 1022 (s), 976 (m), 928 (m), 827 (m), 787 (m), 754 (s), 733 (m); M.p.: orange colouration at *ca.* 75°C, melts at 120°C.

1.4.2 Formation of [(^{Mes}nacnac)Mg(^{Xyl}nacnac)] 6

[{(^{Mes}nacnac)Mg}$_6$C$_{60}$] **2c** (6.0 mg, 2.1 µmol) and [(^{Xyl}nacnac)Li] (4.5 mg, 14.4 µmol, 6.8 equiv.) were added to an NMR tube with J. Young valve and benzene-d_6 (0.6 mL) was added at room temperature. A reaction occurred instantly, forming a colourless solution of [(^{Mes}nacnac)Mg(^{Xyl}nacnac)] **6** and a dark orange-brown precipitate, likely Li_6C_{60}. (NB: an attempt to partially dissolve this material in warm deuterated THF after sonication failed; recording a ^{13}C{^{1}H} NMR spectrum at 60°C of this mixture showed no significant resonances likely due to the high lattice energy of this material.) Monitoring this reaction by ^{7}Li NMR spectroscopy revealed the consumption of [(^{Xyl}nacnac)Li] and the formation of an intermediate with broad ^{7}Li NMR resonance (0.8 ppm) during the formation of the precipitate, see Figure S 56. Data for **6**: ^{1}H NMR (499.9 MHz, benzene-d_6) δ = 1.50 (s, 6H; ^{Xyl}NCC*H*$_3$), 1.53 (s, 6H; ^{Mes}NCC*H*$_3$), 1.90 (br. s, 12H; ^{Mes}*o*-C*H*$_3$), 1.93 (br. s, 12H; ^{Xyl}*o*-C*H*$_3$), 2.21 (s, 6H; ^{Mes}*p*-C*H*$_3$), 4.93 (two s, 1H; γ-C*H*), 6.75 (s, 4H; ^{Mes}Ar-*H*), 6.95 (s, 6H; ^{Xyl}Ar-*H*); ^{13}C{^{1}H} NMR (125.7 MHz, benzene-d_6) δ = 18.6 (^{Mes}NC*C*H$_3$), 18.7 (^{Xyl}NC*C*H$_3$), 20.9 (^{Mes}*p*-*C*H$_3$), 23.75 (^{Xyl}*o*-*C*H$_3$), 23.81 (^{Mes}*o*-*C*H$_3$), 96.5 (*C*H), 124.2 (^{Xyl}Ar-*C*), 128.7 (^{Xyl}Ar-*C*), 129.5 (^{Mes}Ar-*C*), 132.7 (Ar-*C*), 132.9 (Ar-*C*), 133.2 (Ar-*C*), 146.4 (^{Mes}Ar-*C*), 149.0 (^{Xyl}Ar-*C*), 168.8 (^{Xyl}N*C*CH$_3$), 168.9 (^{Mes}N*C*CH$_3$). Note that the latter spectrum involves some coincidental overlapping of resonances.

2 NMR spectroscopy

2.1 General considerations

Unless stated otherwise, NMR spectra were recorded in deuterated benzene and were acquired as *in-situ* products of reactions between C_{60} and $[\{(^{Ar}Nacnac)Mg\}_2]$ **1**. Chemical shifts are given in ppm. Resonance labels in blue correspond to the compound in question, whilst grey labels represent resonances belonging to by-products, impurities or a standard in solution. HMB = hexamethylbenzene (used as an internal standard for some reactions herein). Note that in some reactions not all species may be soluble at all times, *i.e.* they may not be fully dissolved (*e.g.* **1c**, **2d**, **3**) or a poorly soluble preciptate may have formed (*e.g.* black-brown fulleride species). Further information has been provided in the Figure captions.

2.2 NMR spectra of [{(^{Ar}nacnac)Mg}$_n$C$_{60}$]

2.2.1 NMR spectra of [{(^{Dip}nacnac)Mg}$_n$C$_{60}$]

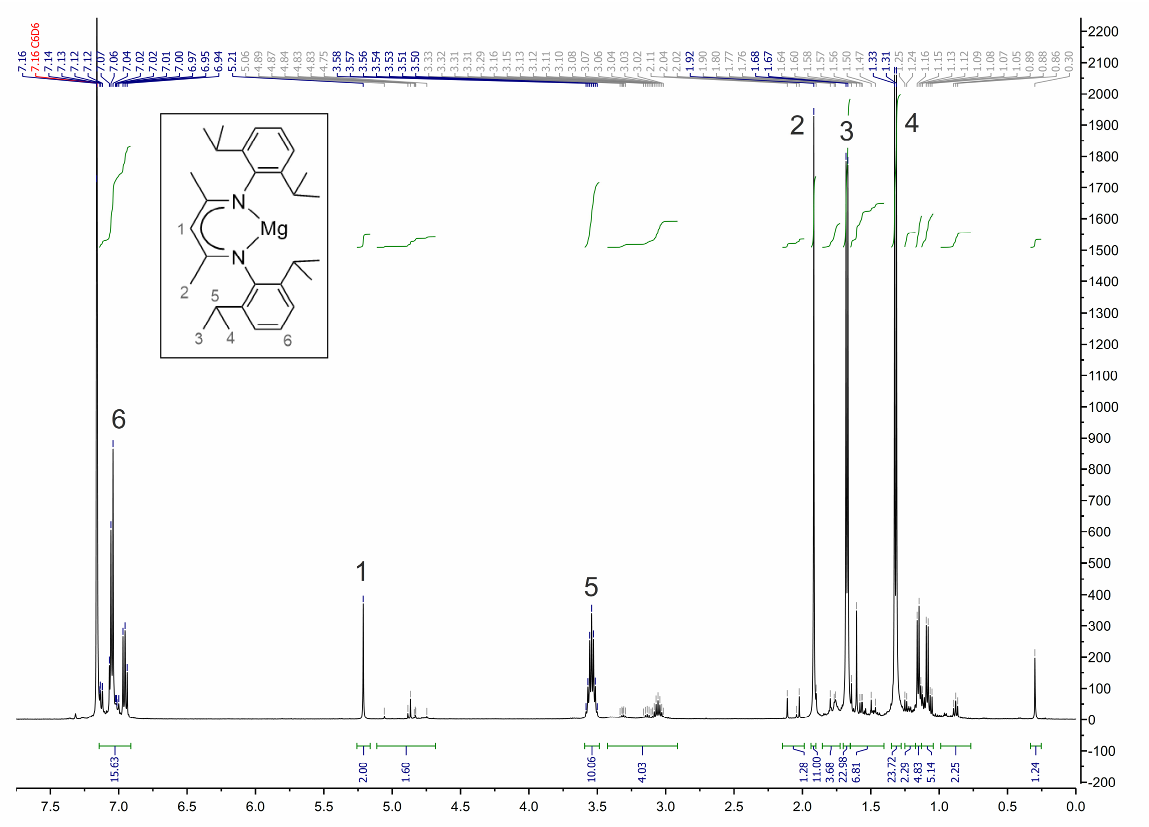

Figure S2. ^{1}H NMR spectrum (499.9 MHz, 295 K) of [{(^{Dip}nacnac)Mg}$_2$C$_{60}$] **3a**.

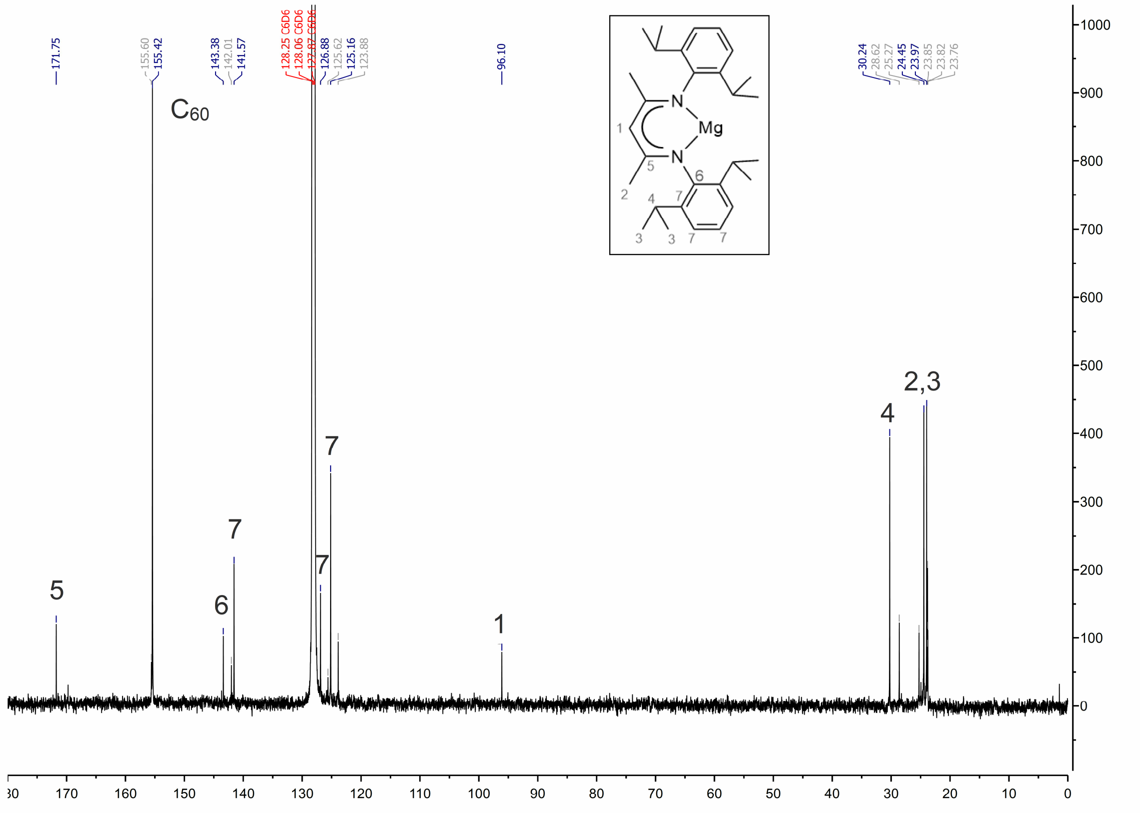

Figure S3. ^{13}C{^{1}H} NMR spectrum (125.7 MHz, 293 K) of [{(^{Dip}nacnac)Mg}$_2$C$_{60}$] **3a**.

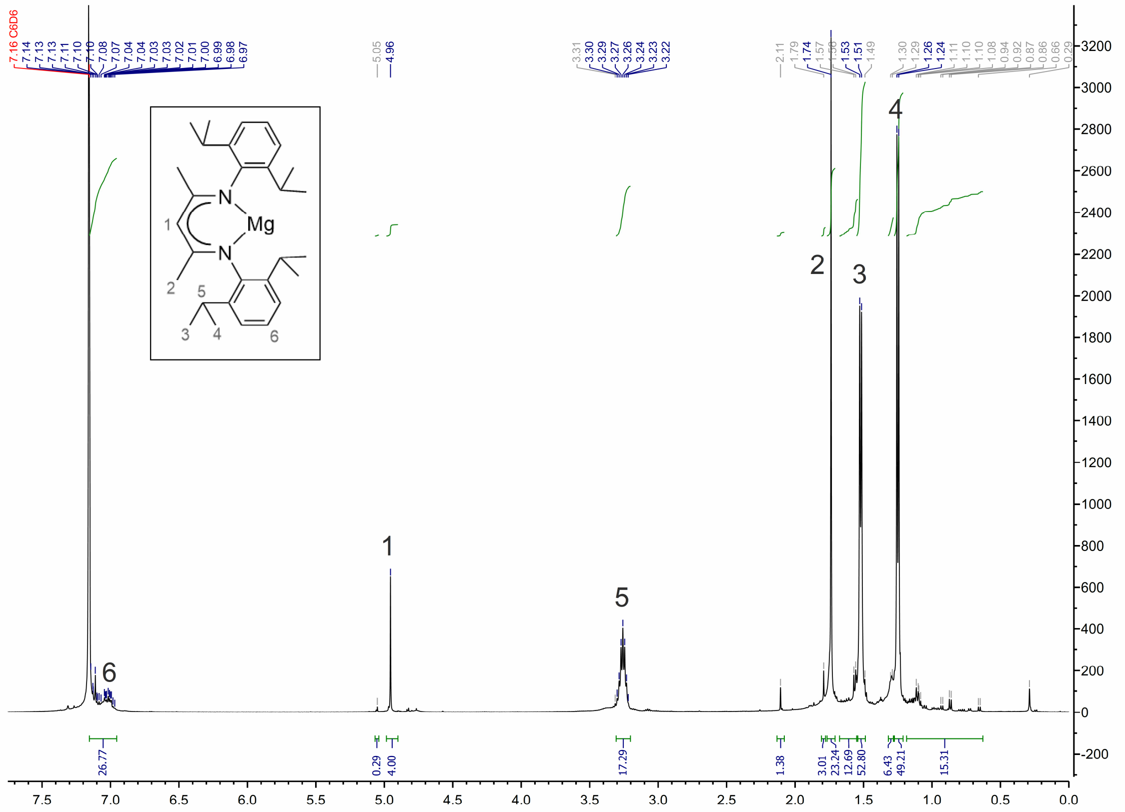

Figure S4. ^{1}H NMR spectrum (499.9 MHz, 295 K) of [{(^{Dip}nacnac)Mg}$_4$C$_{60}$] **4a**.

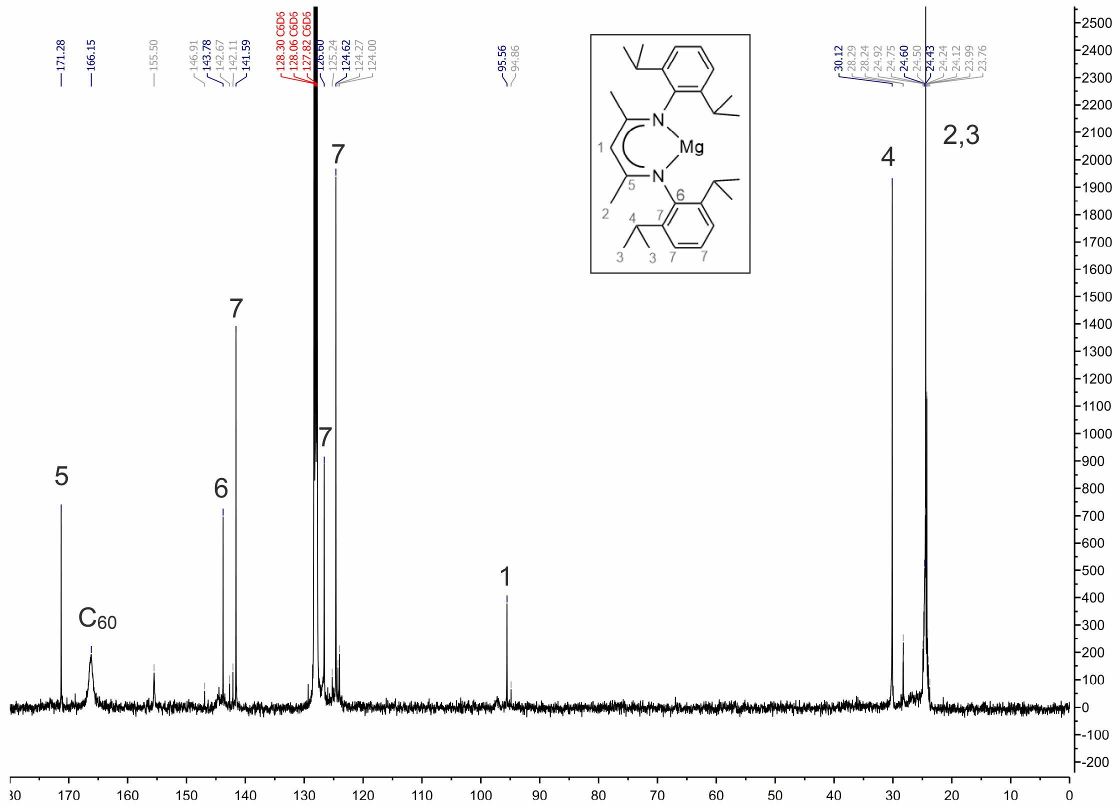

Figure S5. ^{13}C{^{1}H} NMR spectrum (125.7 MHz, 295 K) of [{(^{Dip}nacnac)Mg}$_4$C$_{60}$] **4a**.

2.2.2 NMR spectra of [{(^{Dep}nacnac)Mg}$_n$C$_{60}$]

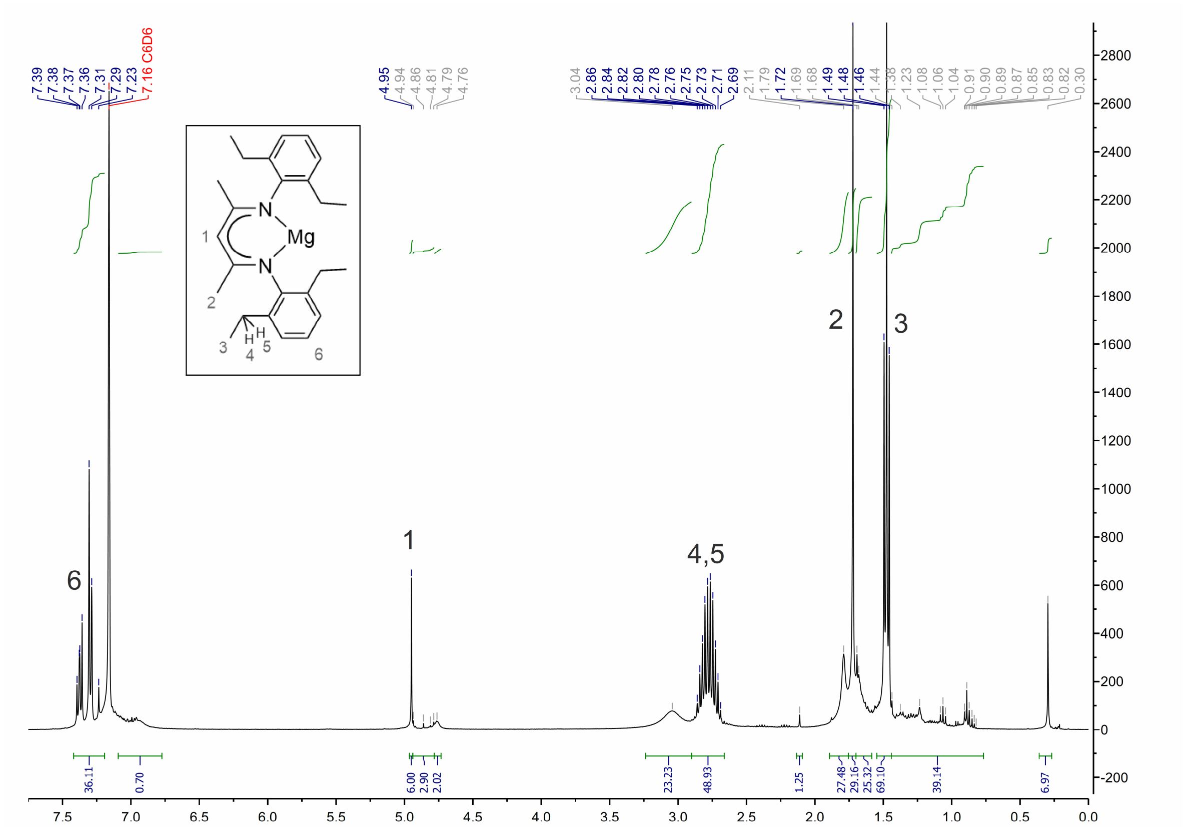

Figure S6. ^{1}H NMR spectrum (400.1 MHz, 295 K) of isolated [{(^{Dep}nacnac)Mg}$_6$C$_{60}$] **2b** in benzene-d_6 recorded soon after dissolution. Observing **2b** as the only species in solution was not possible and resonances corresponding to other [{(^{Dep}nacnac)Mg}$_n$C$_{60}$] species were always observed, for example the broadened resonances for tentatively assigned [{(^{Dep}nacnac)Mg}$_5$C$_{60}$] **5b**.

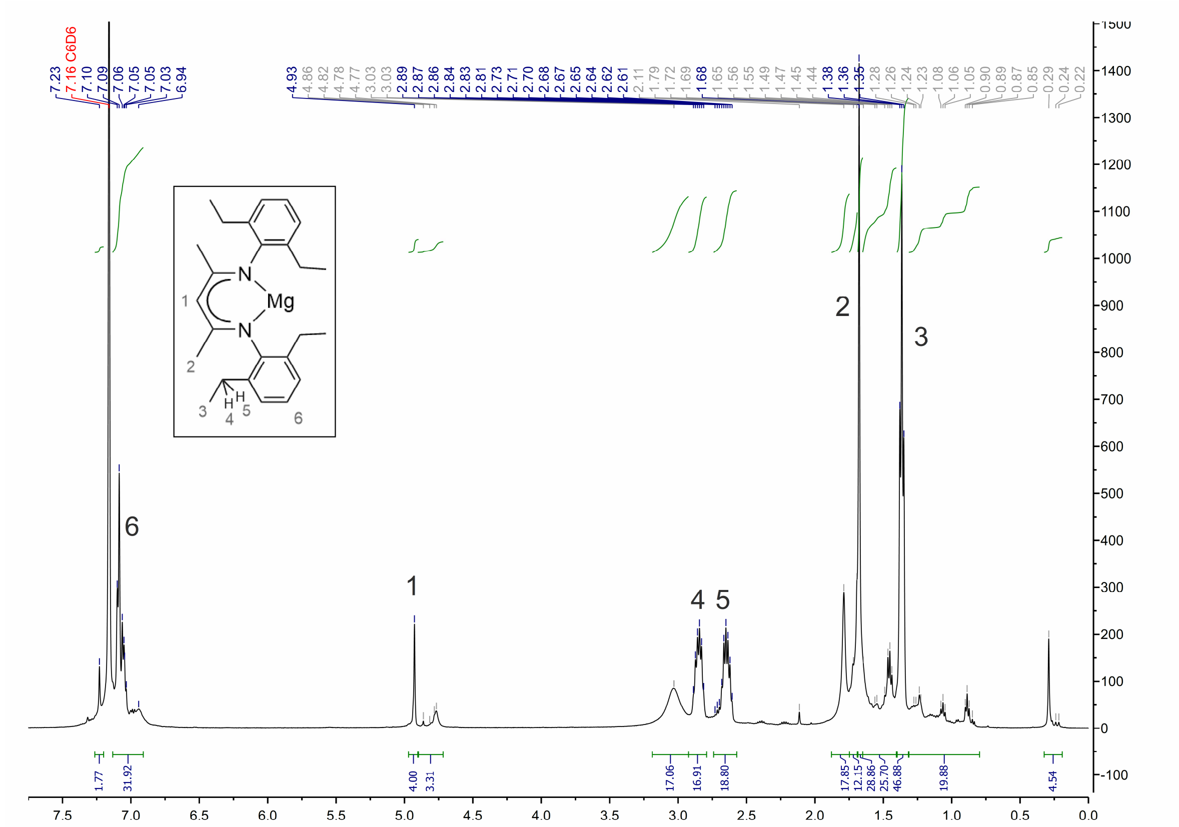

Figure S7. ^{1}H NMR spectrum (400.1 MHz, 295 K) of isolated [{(^{Dep}nacnac)Mg}$_6$C$_{60}$] **2b** taken 60 h after dissolution (re Figure S6). The composition in solution changed over this period of time largely to a mixture of products [{(^{Dep}nacnac)Mg}$_4$C$_{60}$] **4b** and [{(^{Dep}nacnac)Mg}$_5$C$_{60}$] **5b**, with no original **2b** remaining. Only trace amounts of [{(^{Dep}nacnac)Mg}$_2$] **1b** are visible in the spectrum.

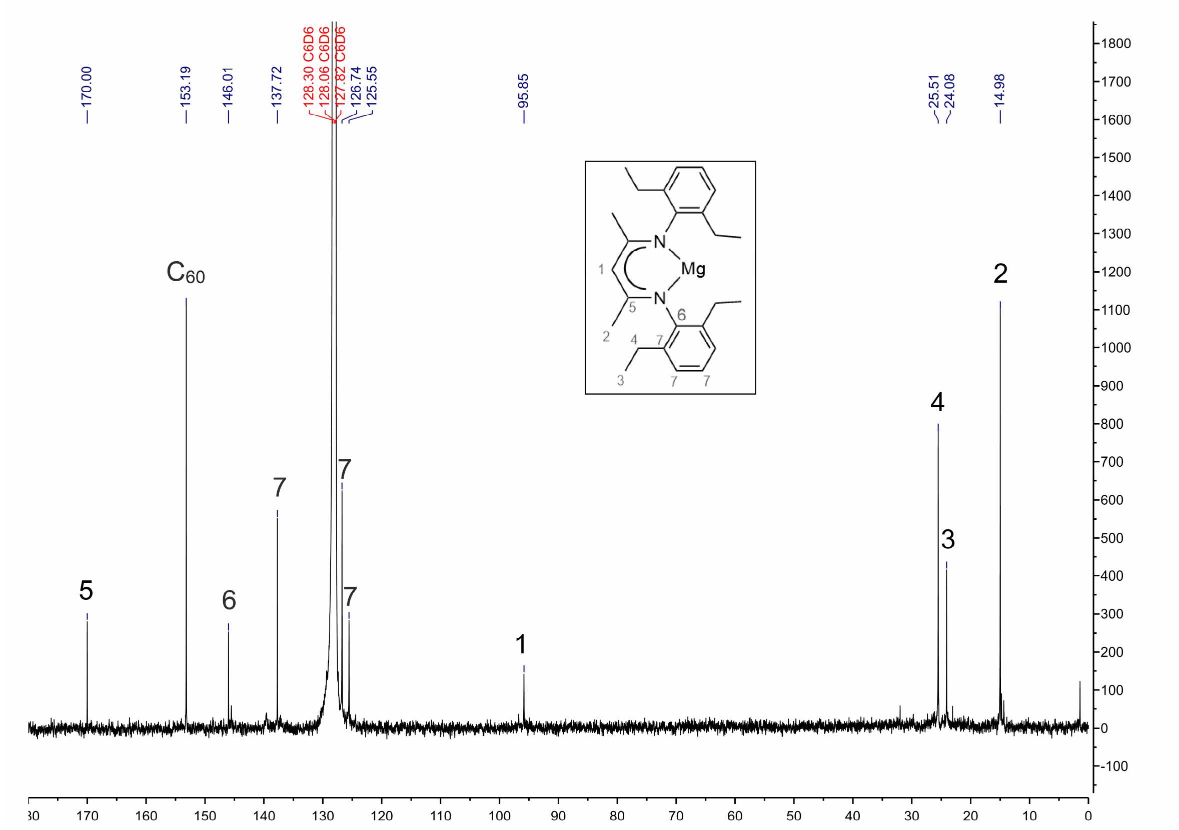

Figure S8. $^{13}C\{^1H\}$ NMR spectrum (100.6 MHz, 295 K) of isolated $[\{(^{Dep}nacnac)Mg\}_6C_{60}]$ **2b** soon after dissolution (re Figure S6).

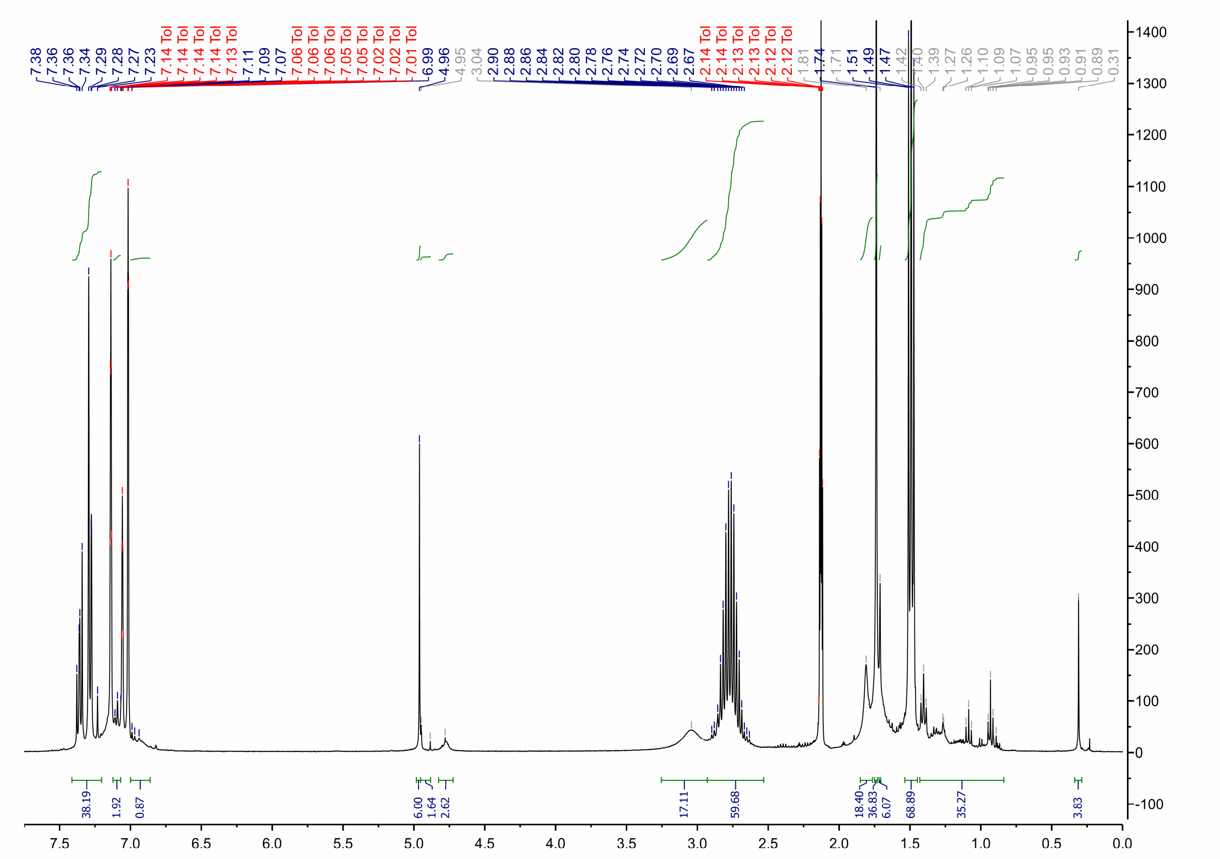

Figure S9. ^{1}H NMR spectrum (400.1 MHz, 295 K) of isolated [{(^{Dep}nacnac)Mg}$_6$C$_{60}$] **2b** in toluene-d_8 (*c.f.* Figure S6) with some quantity of formed tentatively assigned [{(^{Dep}nacnac)Mg}$_5$C$_{60}$] **5b**.

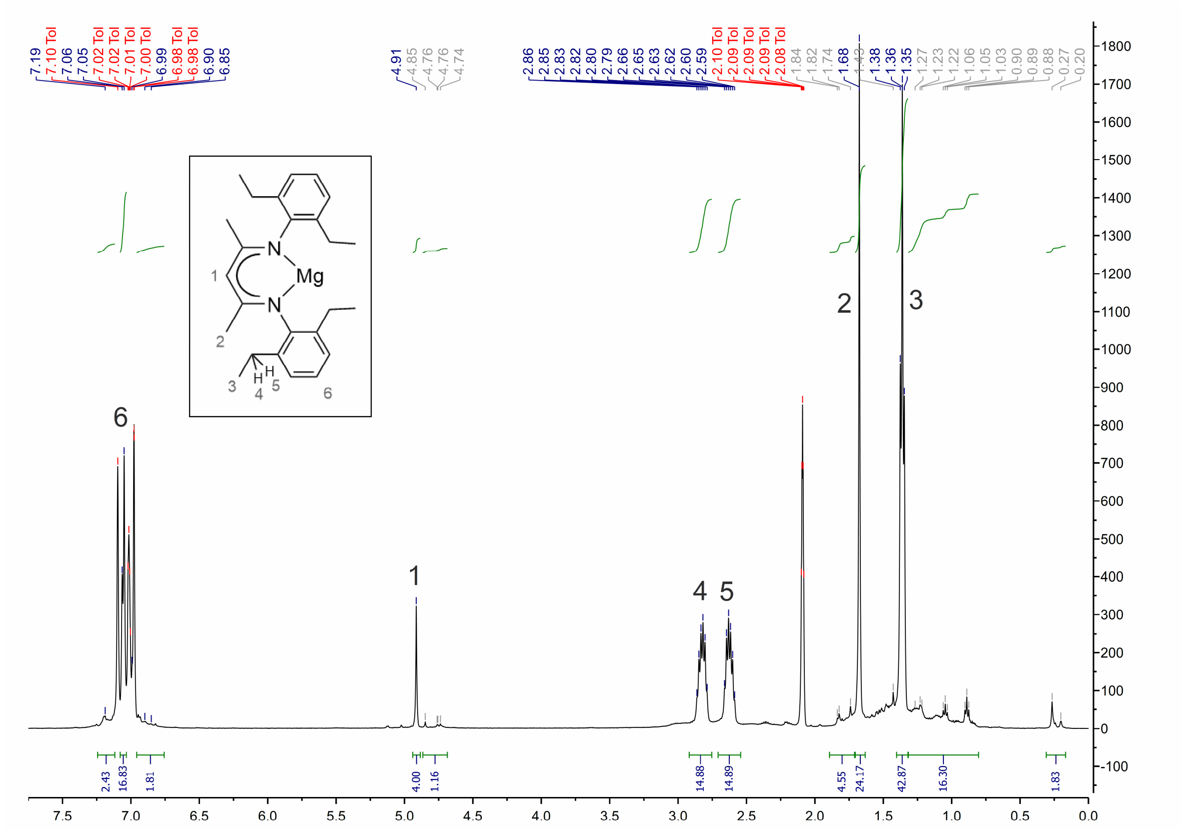

Figure S10. ^{1}H NMR spectrum (400.1 MHz, 295 K) of isolated [{(^{Dep}nacnac)Mg}$_6$C$_{60}$] **2b** in toluene-d_8, taken 48 hours after dissolution. The composition in solution changed over this short period of time to give [{(^{Dep}nacnac)Mg}$_4$C$_{60}$] **4b** as the main species in solution.

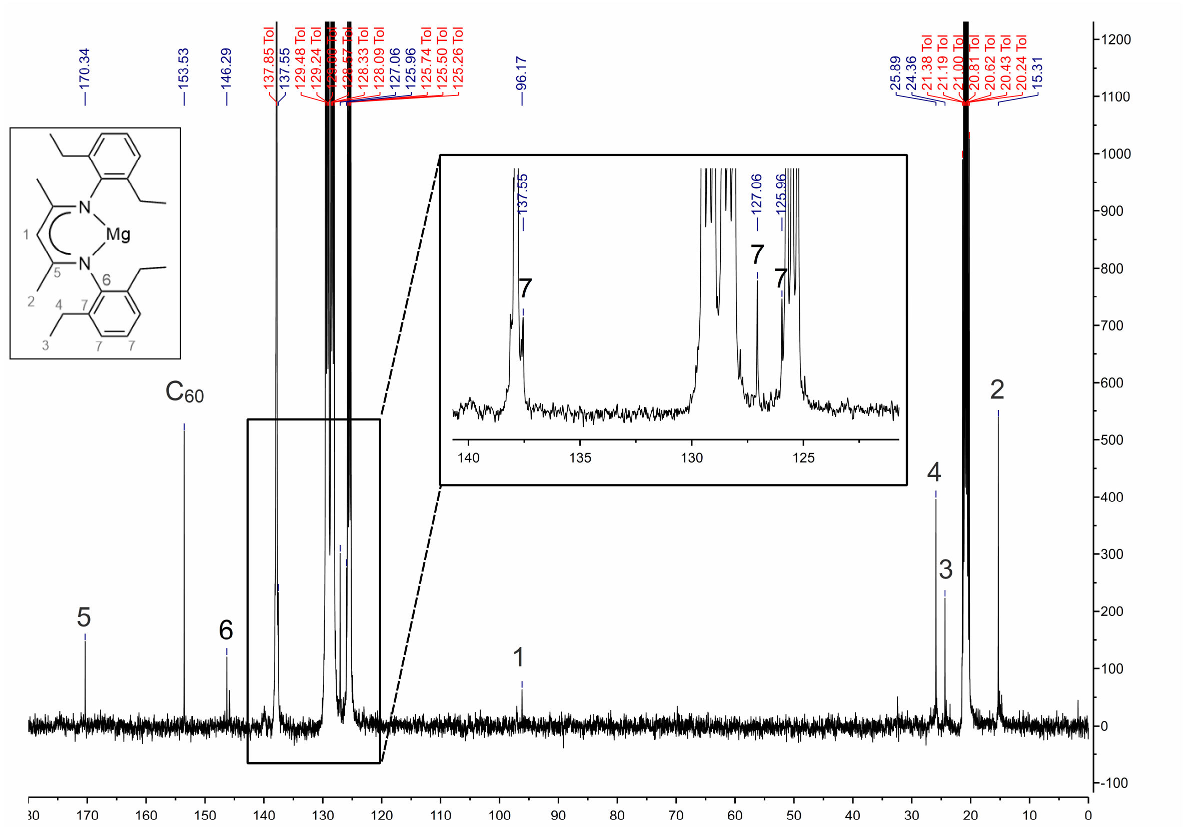

Figure S11. $^{13}C\{^1H\}$ NMR spectrum (100.6 MHz, 295 K) of isolated $[\{(^{Dep}nacnac)Mg\}_6C_{60}]$ **2b** in toluene-d_8.

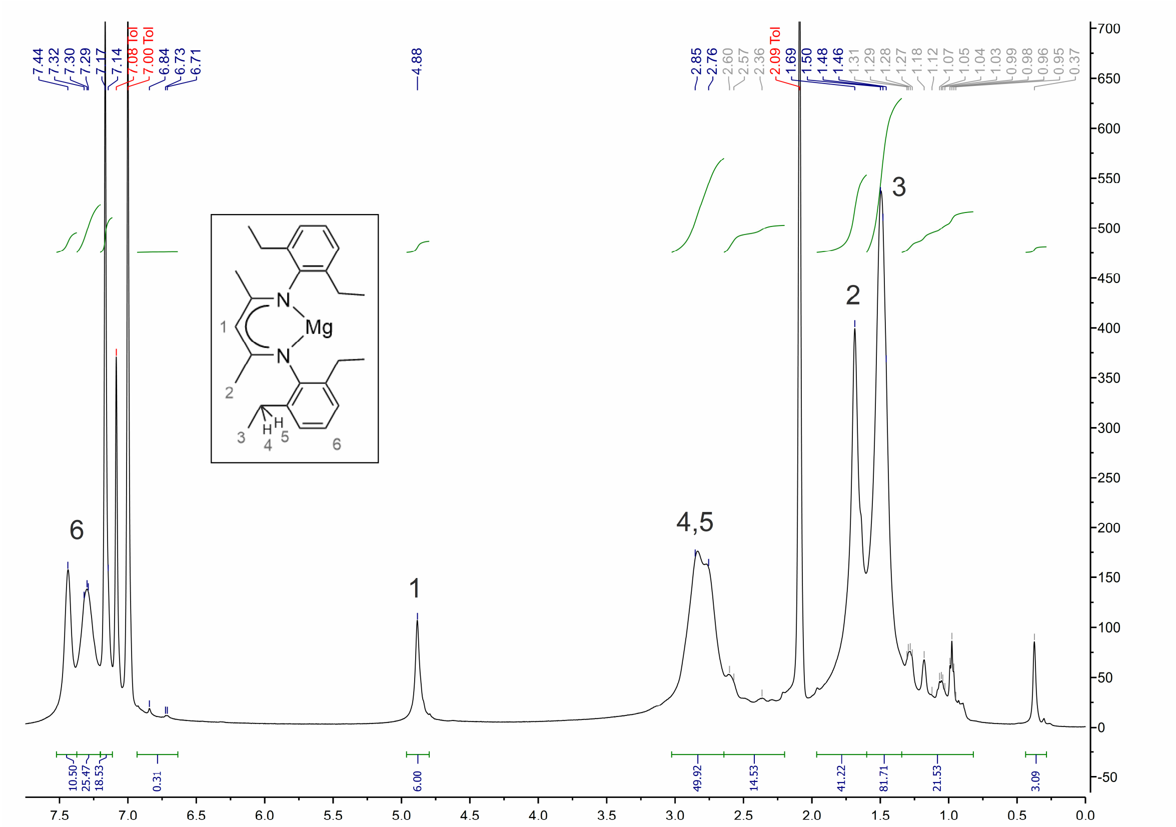

Figure S12. Low temperature ^{1}H NMR spectrum (499.9 MHz, 193 K) of isolated $[\{(^{Dep}nacnac)Mg\}_6C_{60}]$ **2b** in toluene-d_8. Broadening of resonances may arise from the poor solubility of **2b** under these conditions.

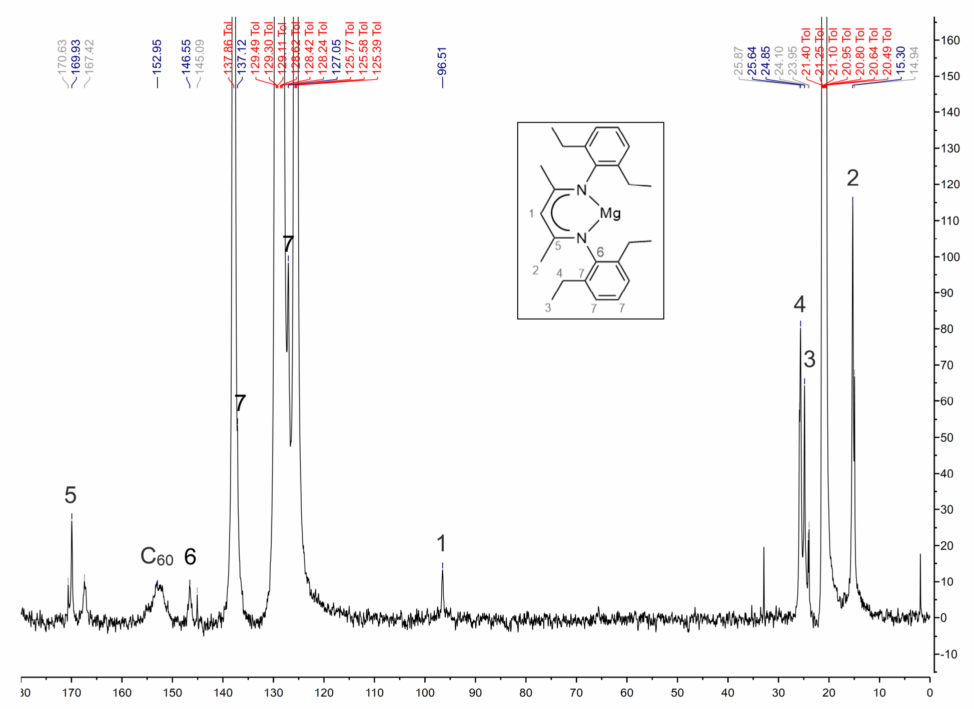

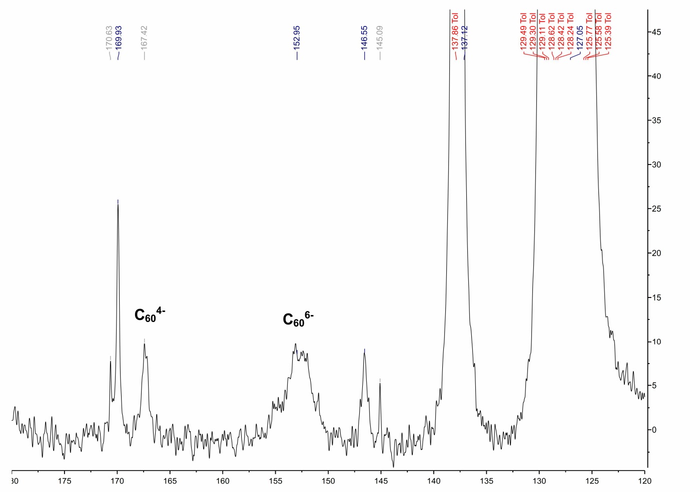

Figure S13. Low temperature $^{13}C\{^1H\}$ NMR spectrum (125.7 MHz, 193 K; full spectrum and 120-180 ppm excerpt) of isolated [{(^{Dep}nacnac)Mg}$_6C_{60}$] **2b** in toluene-d_8, showing the broadened/splitting C_{60}^{6-} resonance. The composition in solution changed whilst acquiring the spectrum, showing resonances corresponding to [{(^{Dep}nacnac)Mg}$_4C_{60}$] **4b** (grey labelling). The concentration of [{(^{Dep}nacnac)Mg}$_4C_{60}$] **4b** in the sample increased at low temperature during spectrum acquisition. Whilst compound **2b** converts in solution over time to **5b** and **4b**, we believe this path would be considerable slowed down at low temperature and would produce more **5b**. We believe the formation of **4b** may be due to the reaction of **2b** with air to form **4b** likely due to a poor seal of the J.Young NMR tube from the cooling process and relatively long acquisition time.

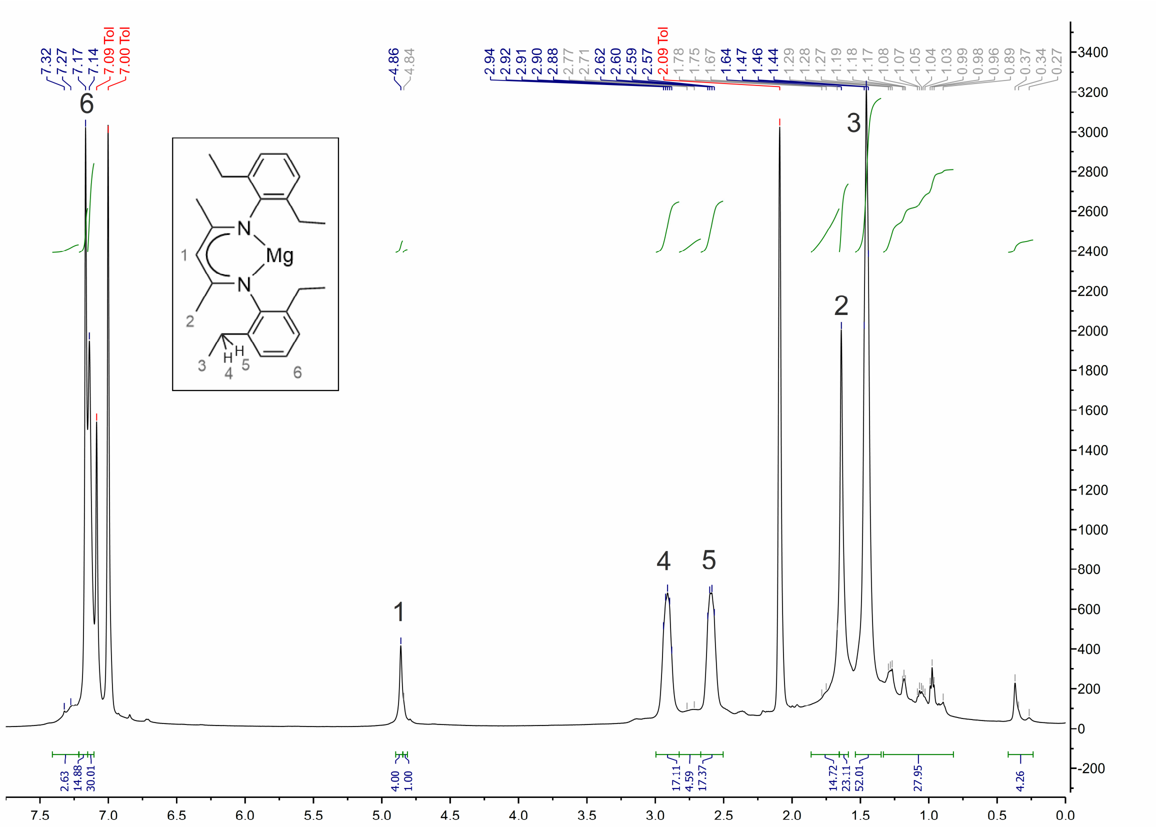

Figure S14. Low temperature ^{1}H NMR spectrum (499.9 MHz, 193 K) of [{(^{Dep}nacnac)Mg}$_4$C$_{60}$] **4b** in toluene-d_8. (This particular sample was obtained from [{(^{Dep}nacnac)Mg}$_6$C$_{60}$] **2b** in toluene-d_8, 48 hours after dissolution. The composition in solution changed over this period of time largely to **4b**.) Broadening of resonances is believed to arise from the poor solubility of **4b** in toluene-d_8 at low temperatures.

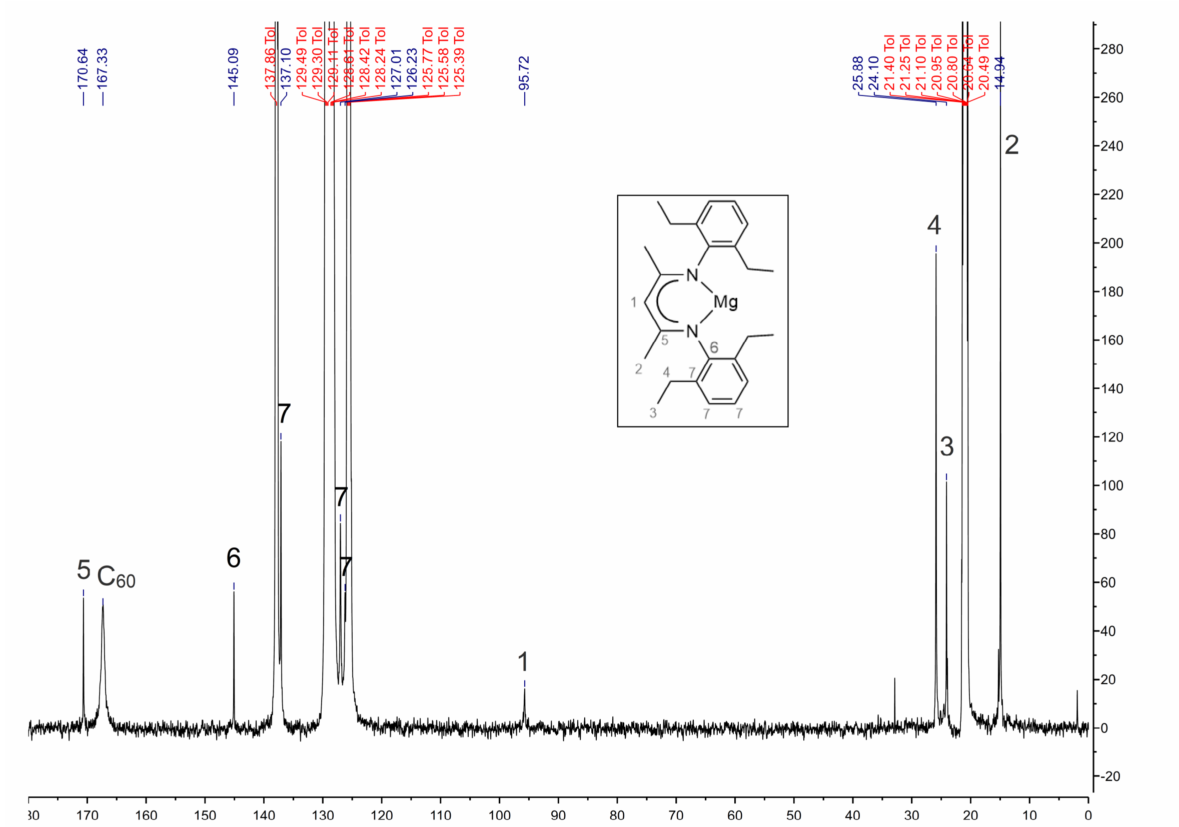

Figure S15. Low temperature $^{13}C\{^1H\}$ NMR spectrum (125.7 MHz, 193 K) of $[\{(^{Dep}nacnac)Mg\}_4C_{60}]$ **4b** in toluene-d_8. (This particular sample was obtained from $[\{(^{Dep}nacnac)Mg\}_6C_{60}]$ **2b** in toluene-d_8, 48 hours after dissolution. The composition in solution changed over this period of time largely to **4b**.)

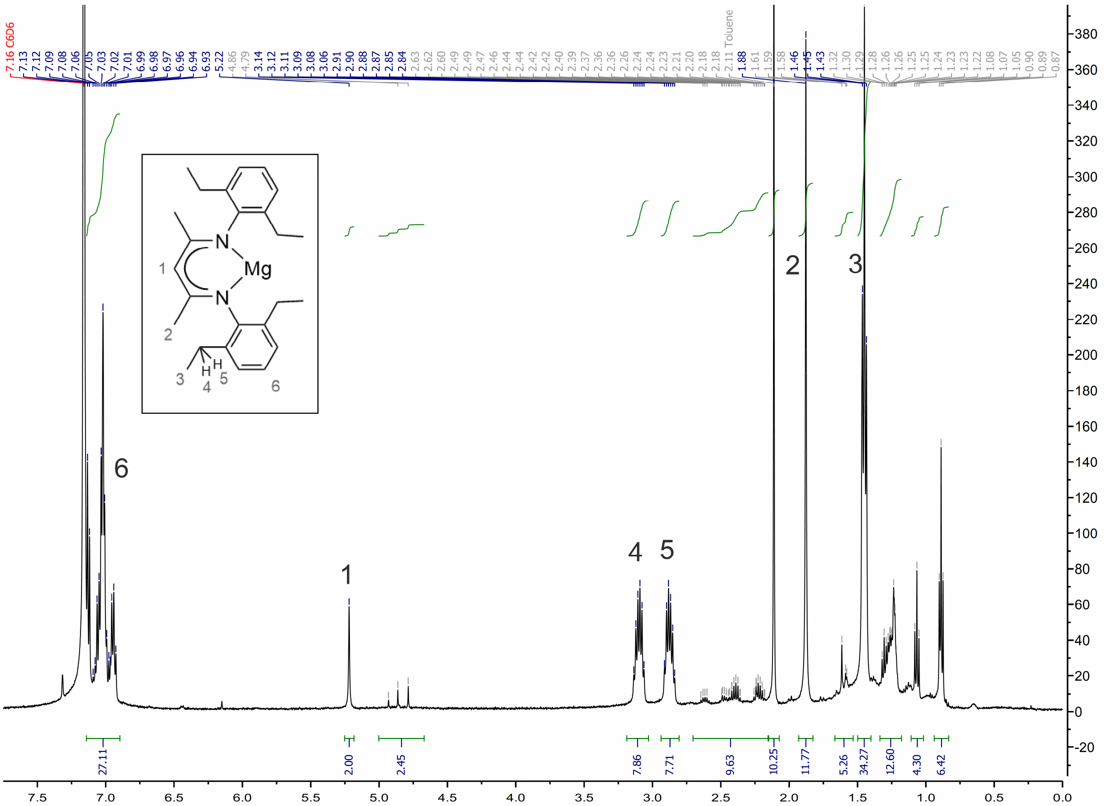

Figure S16. ^{1}H NMR spectrum (499.9 MHz, 295 K) of isolated [{(^{Dep}nacnac)Mg}$_2$C$_{60}$] **3b**.

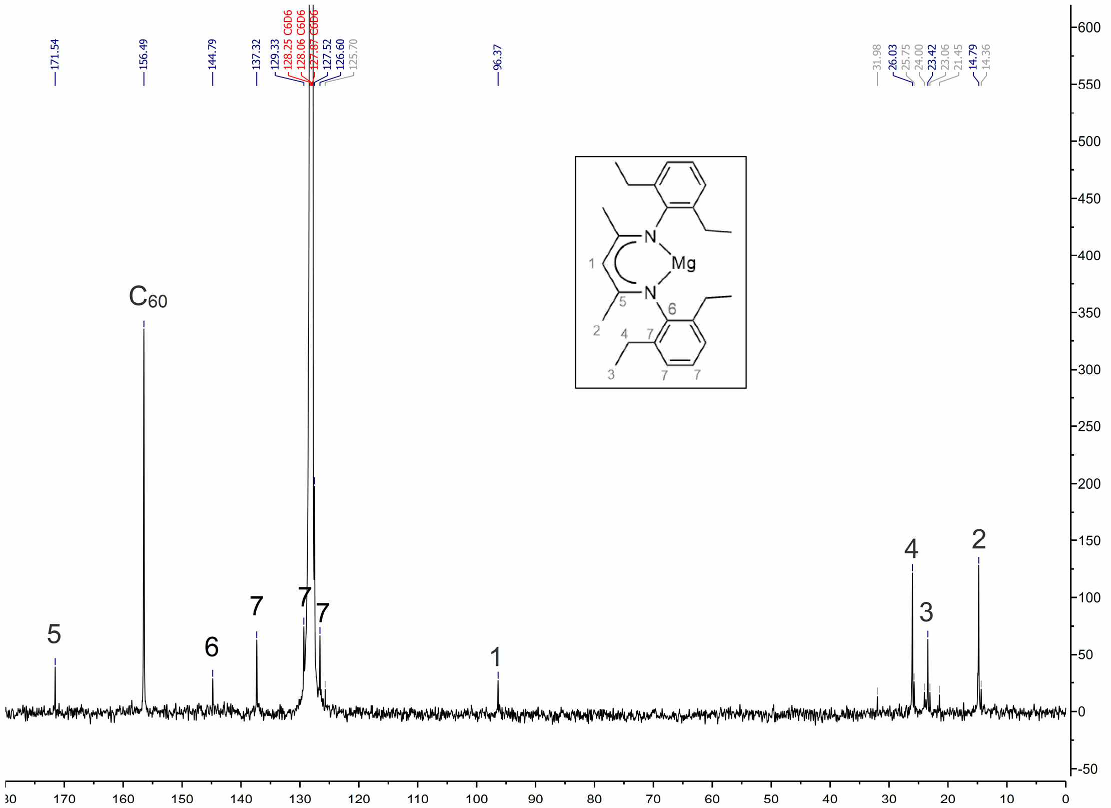

Figure S17. ^{13}C{^{1}H} NMR spectrum (125.7 MHz, 295 K) of isolated [{(^{Dep}nacnac)Mg}$_2$C$_{60}$] **3b**.

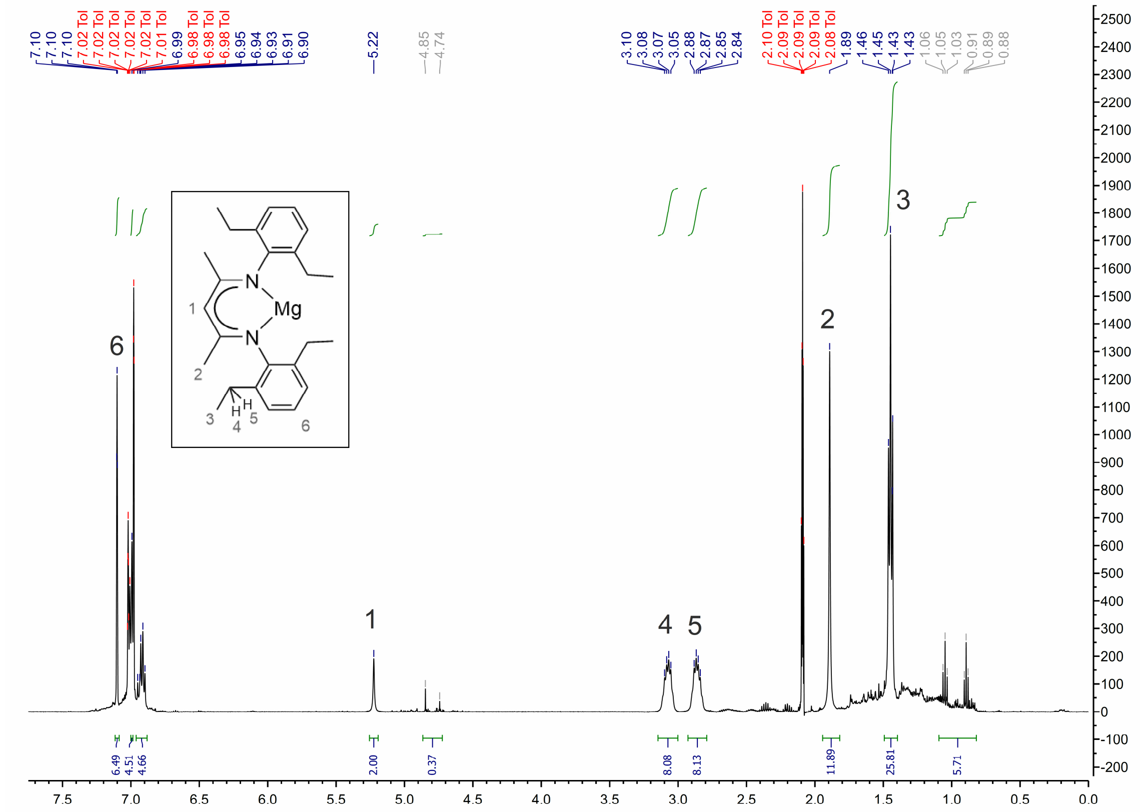

Figure S18. ^{1}H NMR spectrum (499.9 MHz, 295 K) of [{(^{Dep}nacnac)Mg}$_2$C$_{60}$] **3b** in toluene-d_8.

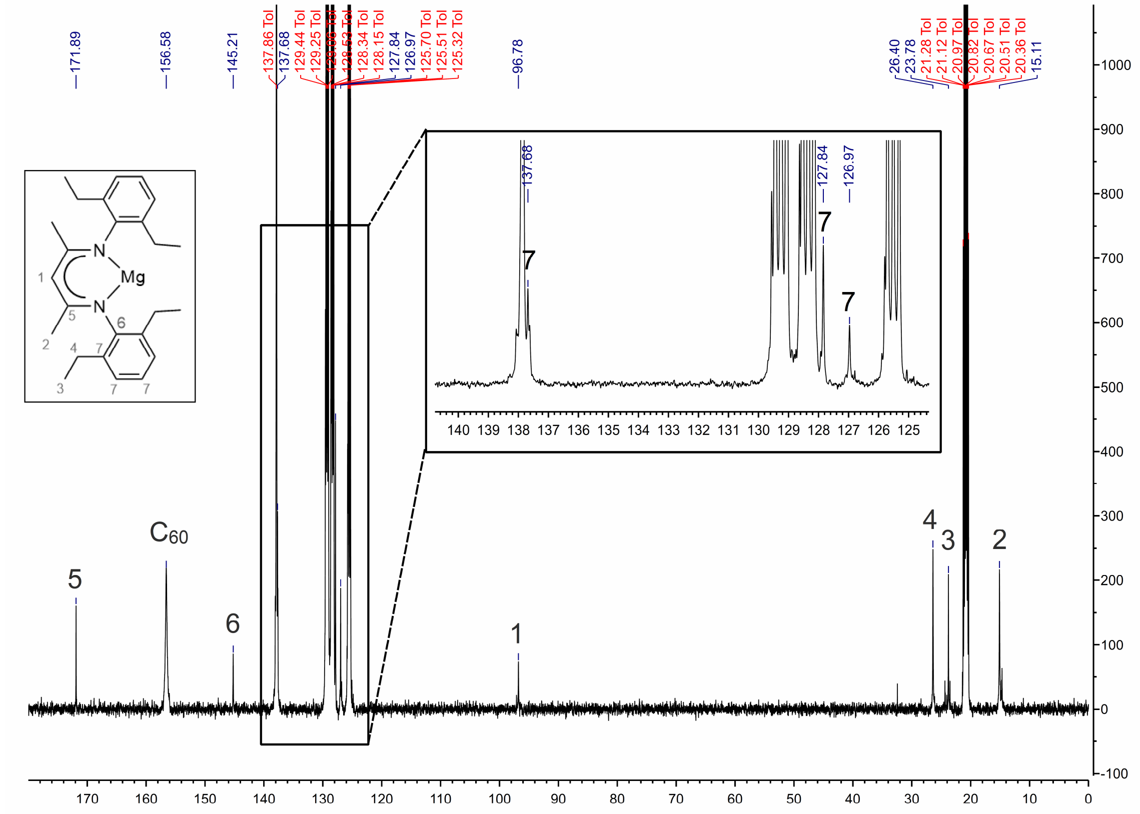

Figure S19. ^{13}C{^{1}H} NMR spectrum (100.6 MHz, 295 K) of [{(^{Dep}nacnac)Mg}$_2$C$_{60}$] **3b** in toluene-d_8.

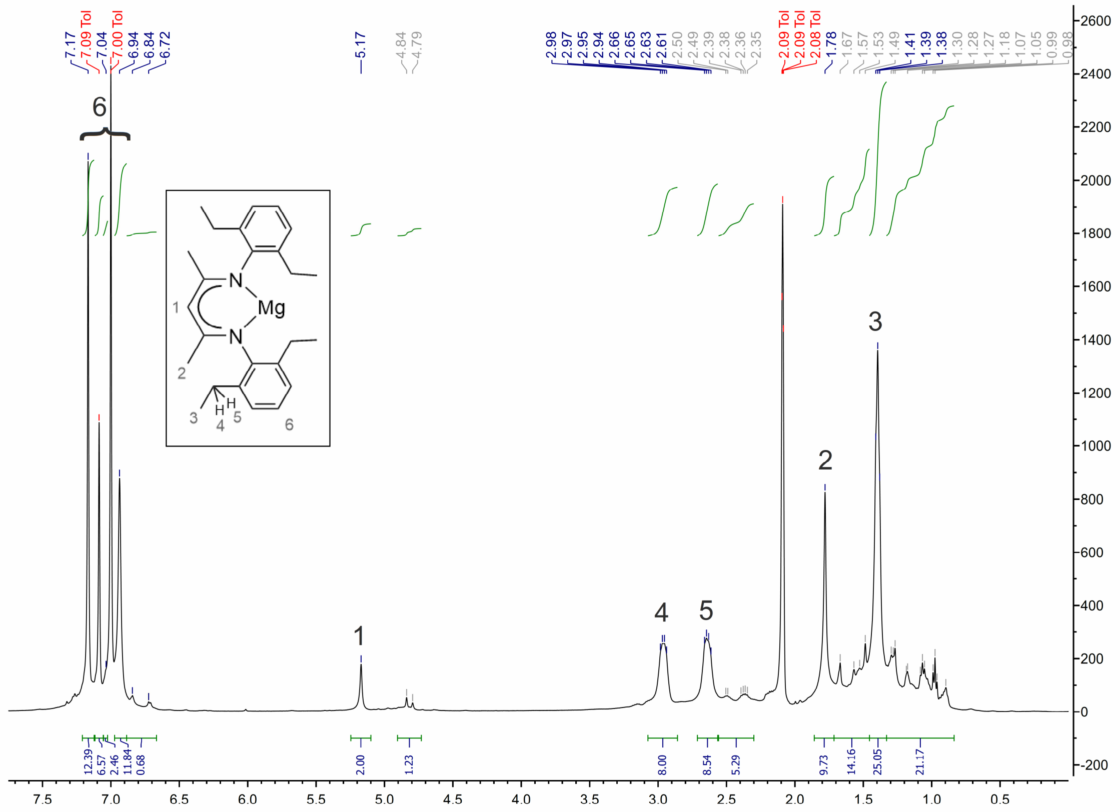

Figure S20. Low temperature ^{1}H NMR spectrum (499.9 MHz, 193 K) of [{(^{Dep}nacnac)Mg}$_2$C$_{60}$] **3b** in toluene-d_8.

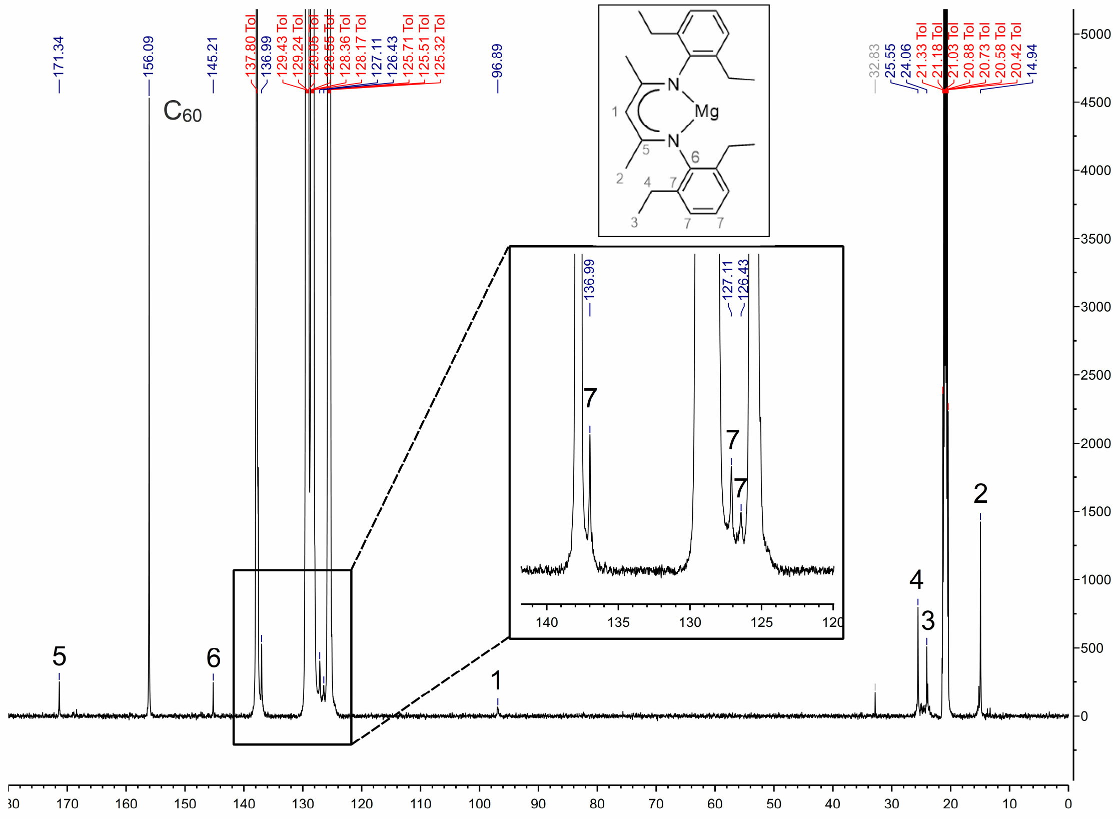

Figure S21. Low temperature ^{13}C{^{1}H} NMR spectrum (125.7 MHz, 193 K) of [{(^{Dep}nacnac)Mg}$_2$C$_{60}$] **3b** in toluene-d_8.

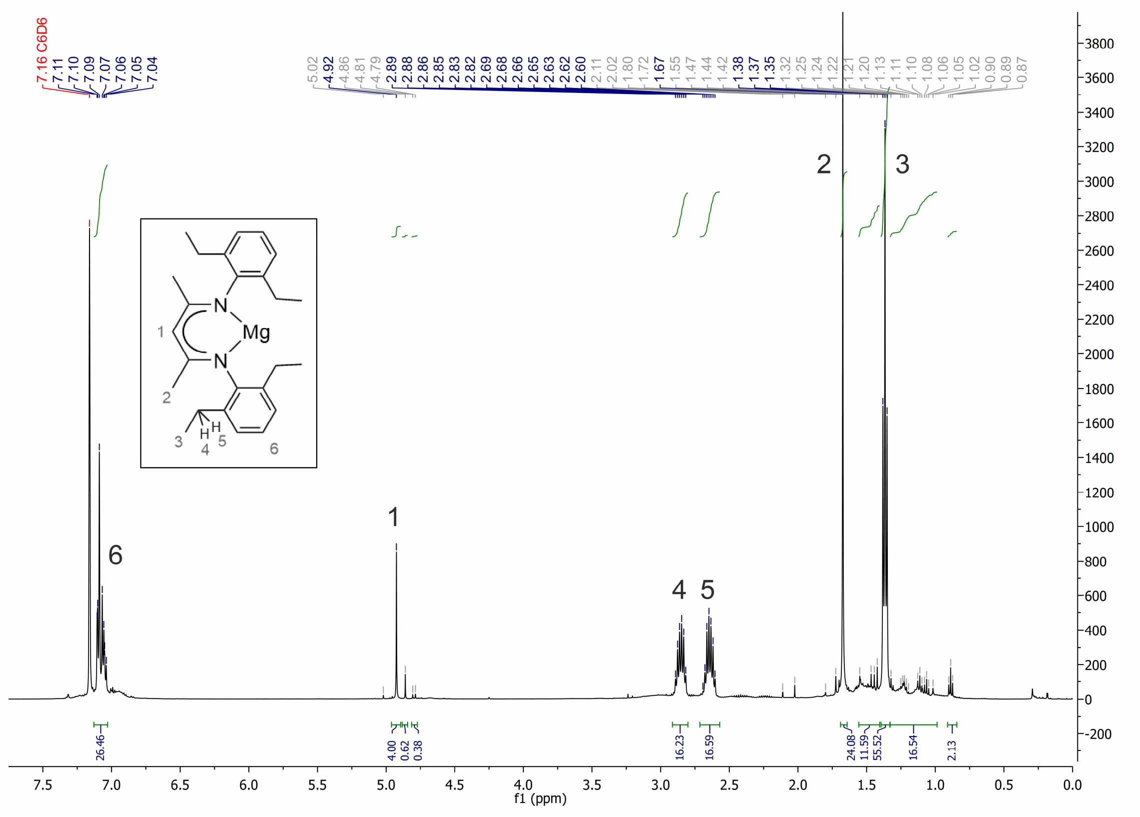

Figure S22. ^{1}H NMR spectrum (499.9 MHz, 295 K) of $[\{(^{Dep}nacnac)Mg\}_4C_{60}]$ **4b**.

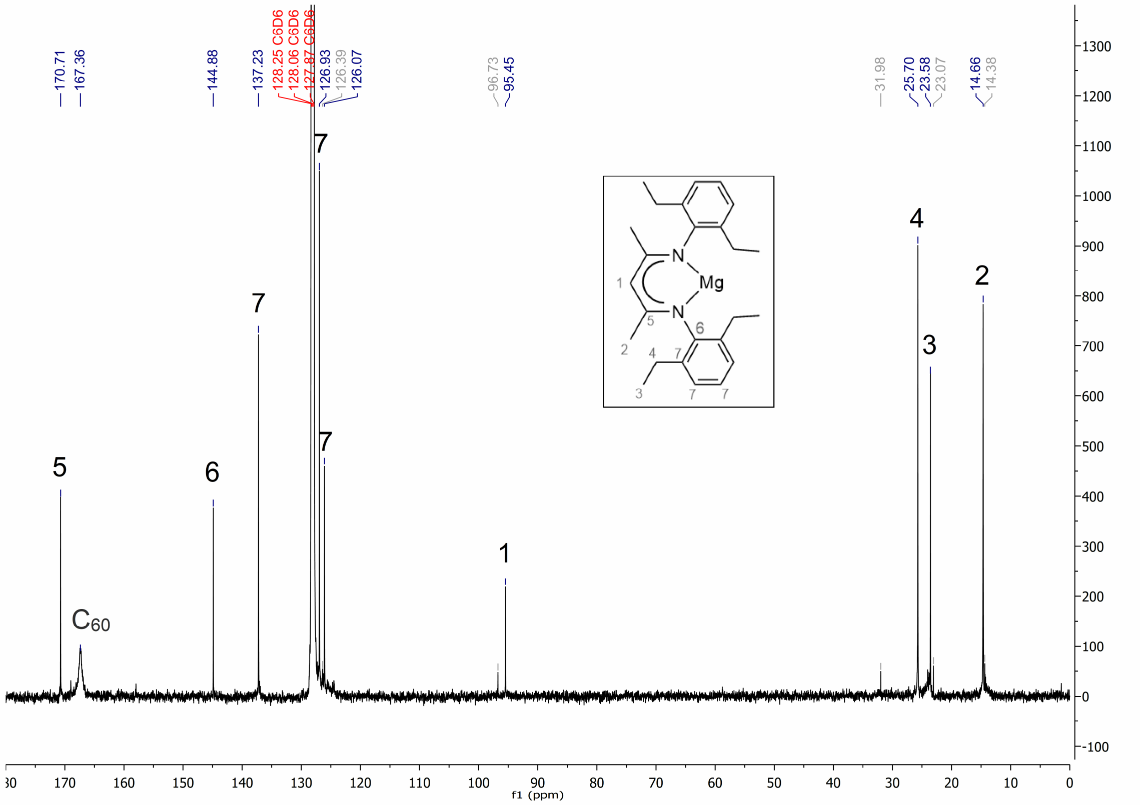

Figure S23. $^{13}C\{^{1}H\}$ NMR spectrum (125.7 MHz, 295 K) of $[\{(^{Dep}nacnac)Mg\}_4C_{60}]$ **4b**.

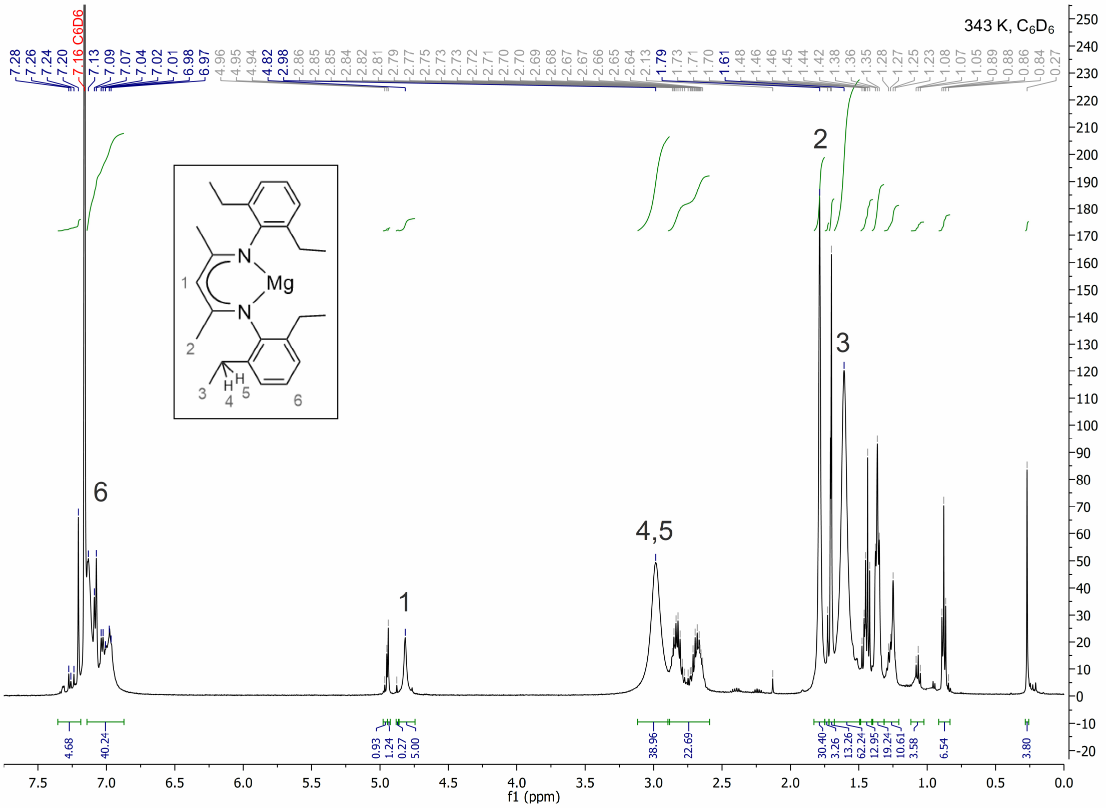

Figure S24. ^{1}H NMR spectrum (499.9 MHz, 343 K) obtained by dissolving isolated crystalline $[\{(^{Dep}nacnac)Mg\}_6C_{60}]$ **2b**, showing a product mixture that changed over time in solution. The broad resonances (blue peaks) are tentatively assigned as $[\{(^{Dep}nacnac)Mg\}_5C_{60}]$ **5b** and decrease slowly over time.

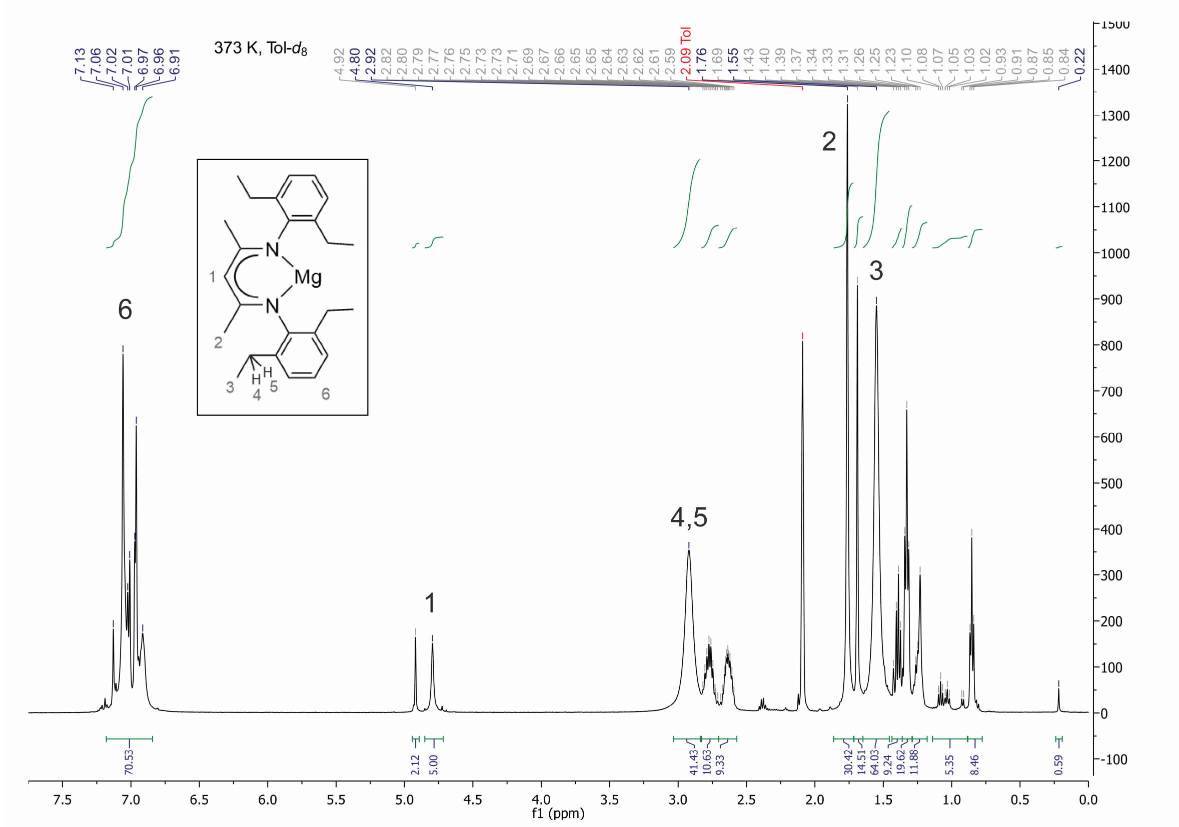

Figure S25. ^{1}H NMR spectrum (499.9 MHz, 373 K) obtained by dissolving isolated crystalline [{(^{Dep}nacnac)Mg}$_6$C$_{60}$] **2b** in toluene-d_8, showing a product mixture that changed over time in solution. The broad resonances (blue peaks) are tentatively assigned as [{(^{Dep}nacnac)Mg}$_5$C$_{60}$] **5b** and are decreasing slowly over time.

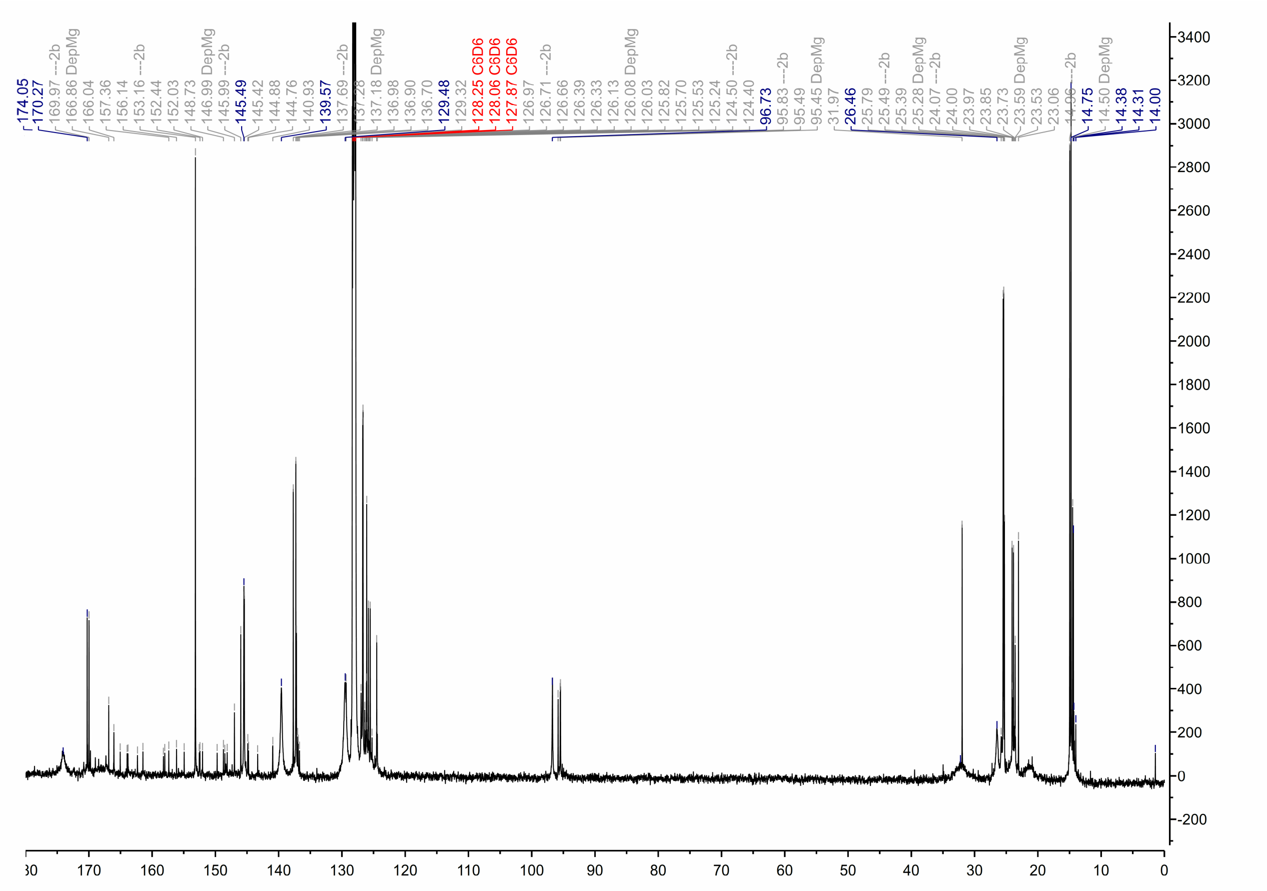

Figure S26. $^{13}C\{^1H\}$ NMR spectrum (125.7 MHz, 295 K) obtained by dissolving isolated crystalline [{(^{Dep}nacnac)Mg}$_6C_{60}$] **2b** in benzene-d_6 and adding 0.33 equivalents of [{(^{Dep}Nacnac)Mg}$_2$] **1b**, showing a product mixture that changed over time in solution. Attempts were made to tentatively assign selected carbon resonances to putative [{(^{Dep}nacnac)Mg}$_5C_{60}$] **5b**.

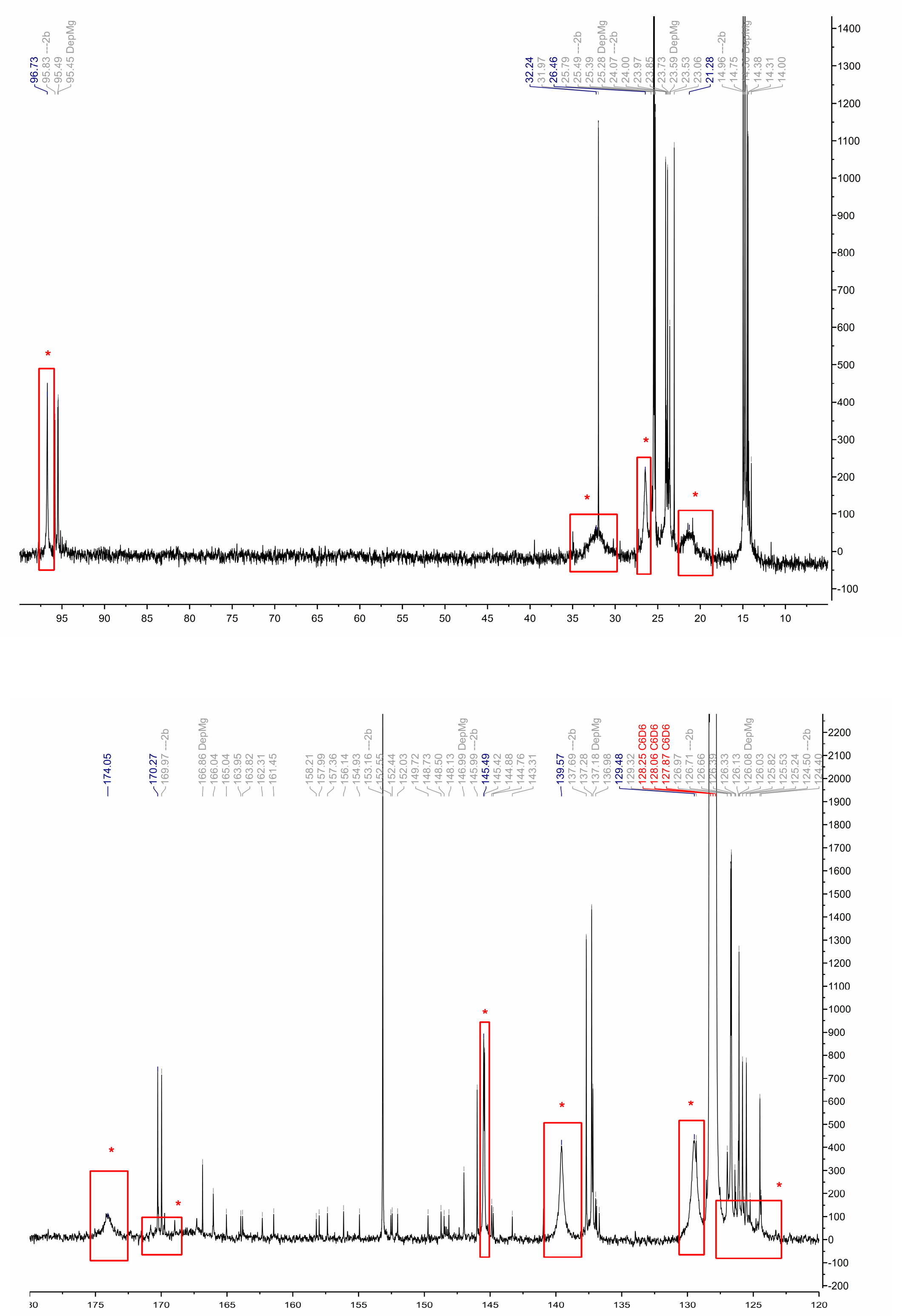

Figure S27. Two excerpts from the previous $^{13}C\{^{1}H\}$ NMR spectrum (Figure S26, 125.7 MHz, 295 K): 0-100 ppm (top), 120-180 ppm (bottom). Red boxes (and approx. blue labelled peaks) with asterisks give some resonances tentatively assigned to putative $[\{(^{Dep}nacnac)Mg\}_5C_{60}]$ **5b**.

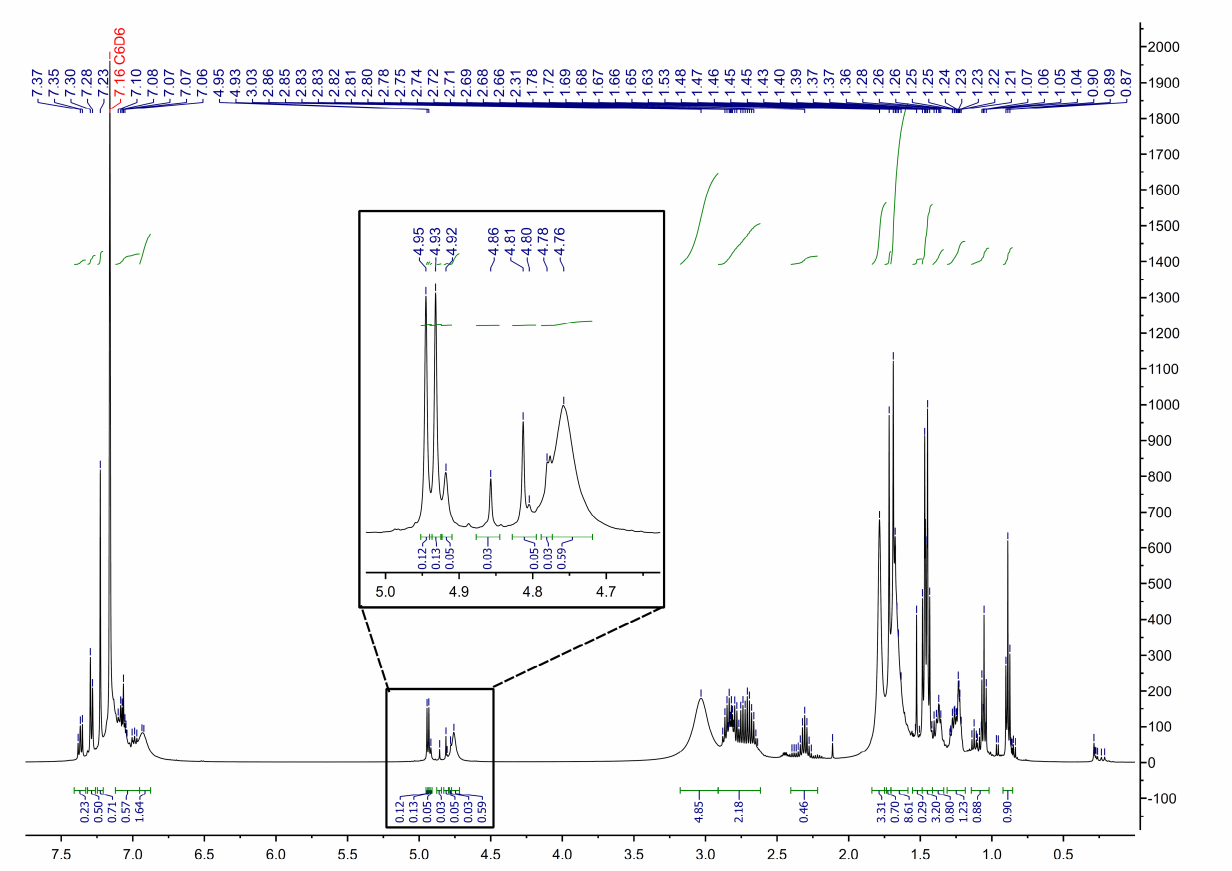

Figure S28. ^{1}H NMR spectrum (499.9 MHz, 295 K) that accompanies the product mixture for Figures S26-27, and the tentative assignment of [{(^{Dep}nacnac)Mg}$_5$C$_{60}$] **5b**.

2.2.3 NMR spectra of [{(^{Mes}nacnac)Mg}$_n$C$_{60}$]

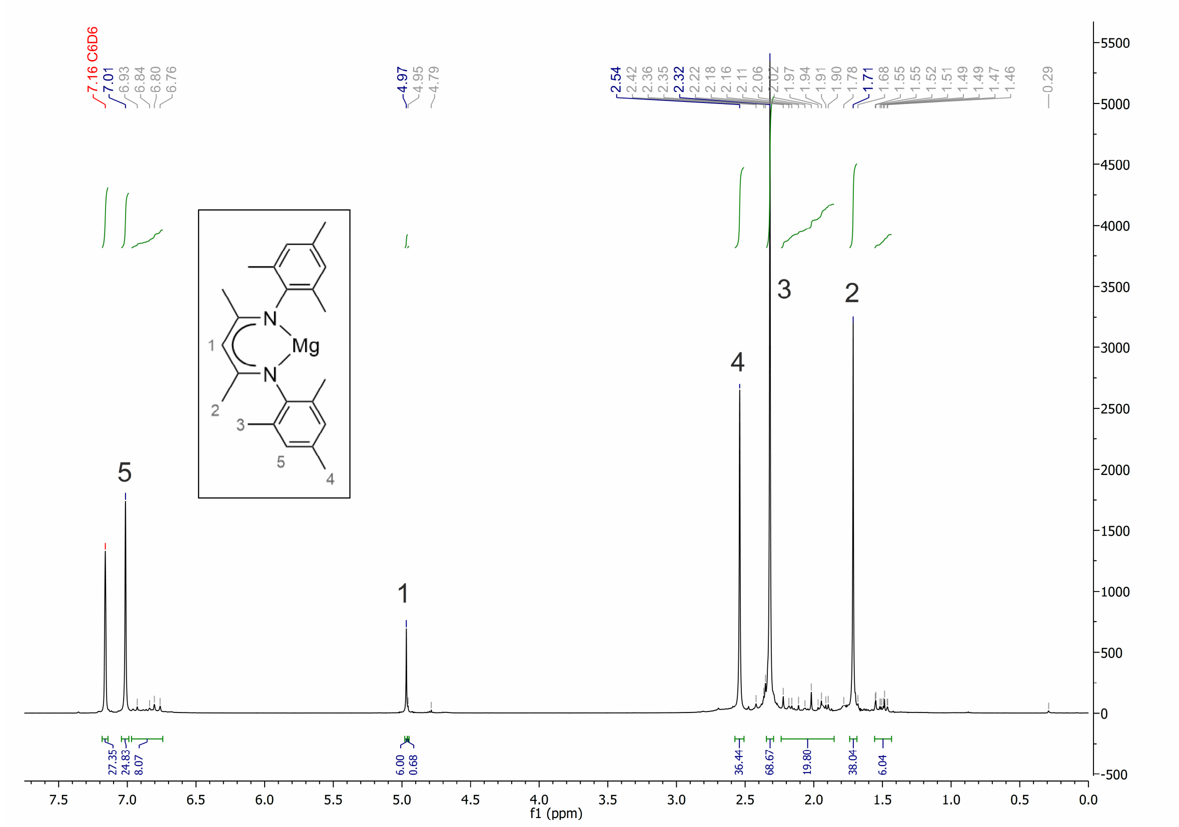

Figure S29. ^{1}H NMR spectrum (499.9 MHz, 295 K) of isolated [{(^{Mes}nacnac)Mg}$_6$C$_{60}$] **2c**.

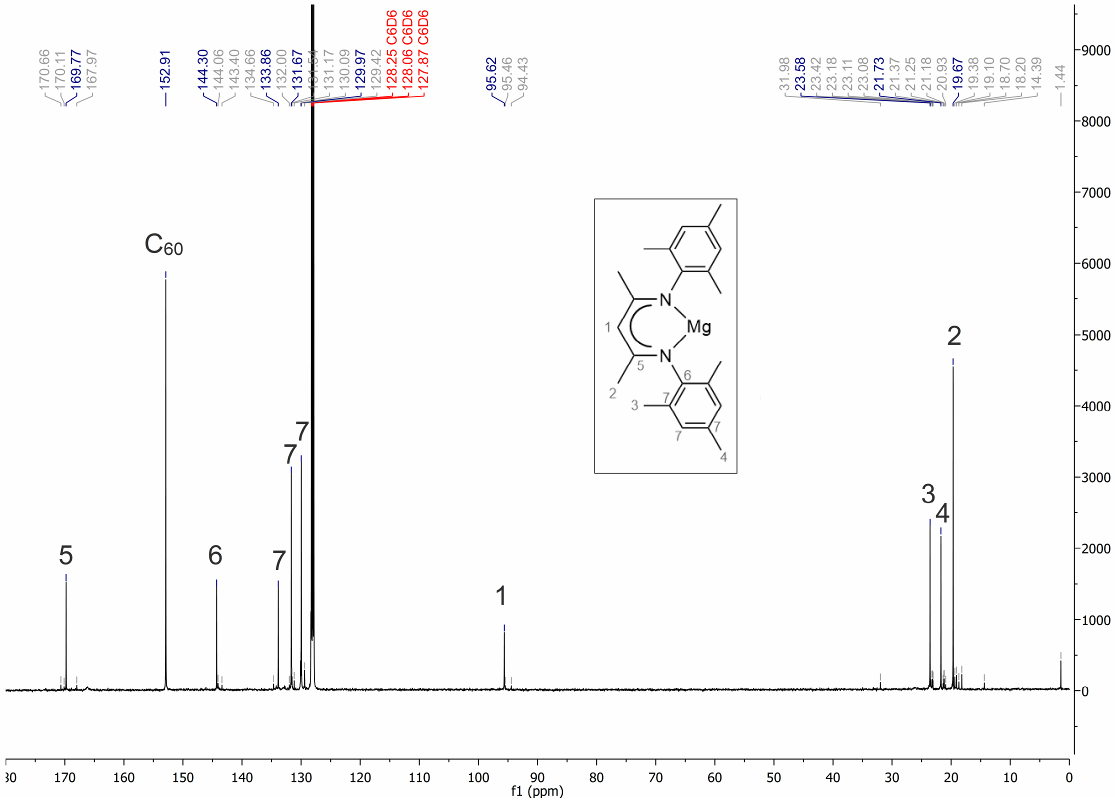

Figure S30. ^{13}C{^{1}H} NMR spectrum (125.7 MHz, 295 K) of isolated [{(^{Mes}nacnac)Mg}$_6$C$_{60}$] **2c**.

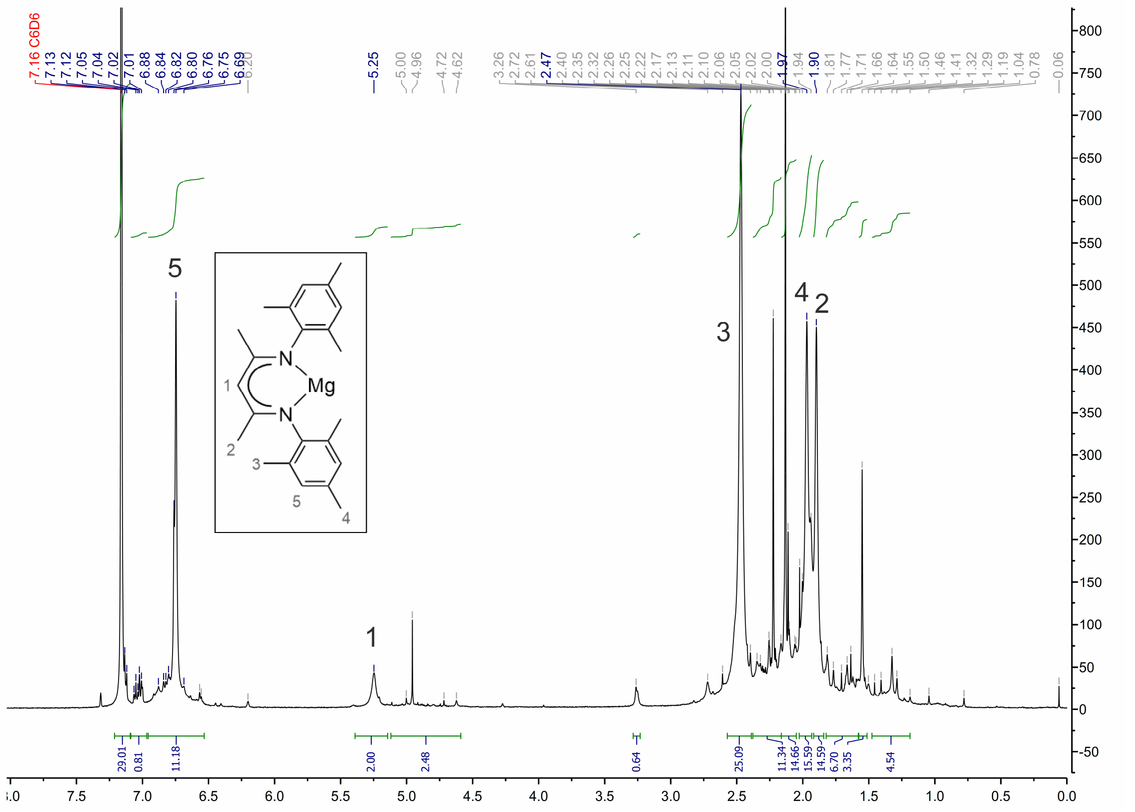

Figure S31. ^{1}H NMR spectrum (499.9 MHz, 295 K) of [{(^{Mes}nacnac)Mg}$_2$C$_{60}$] **3c**. The broadening of the resonances is believed to result from the poor solubility of **3c**.

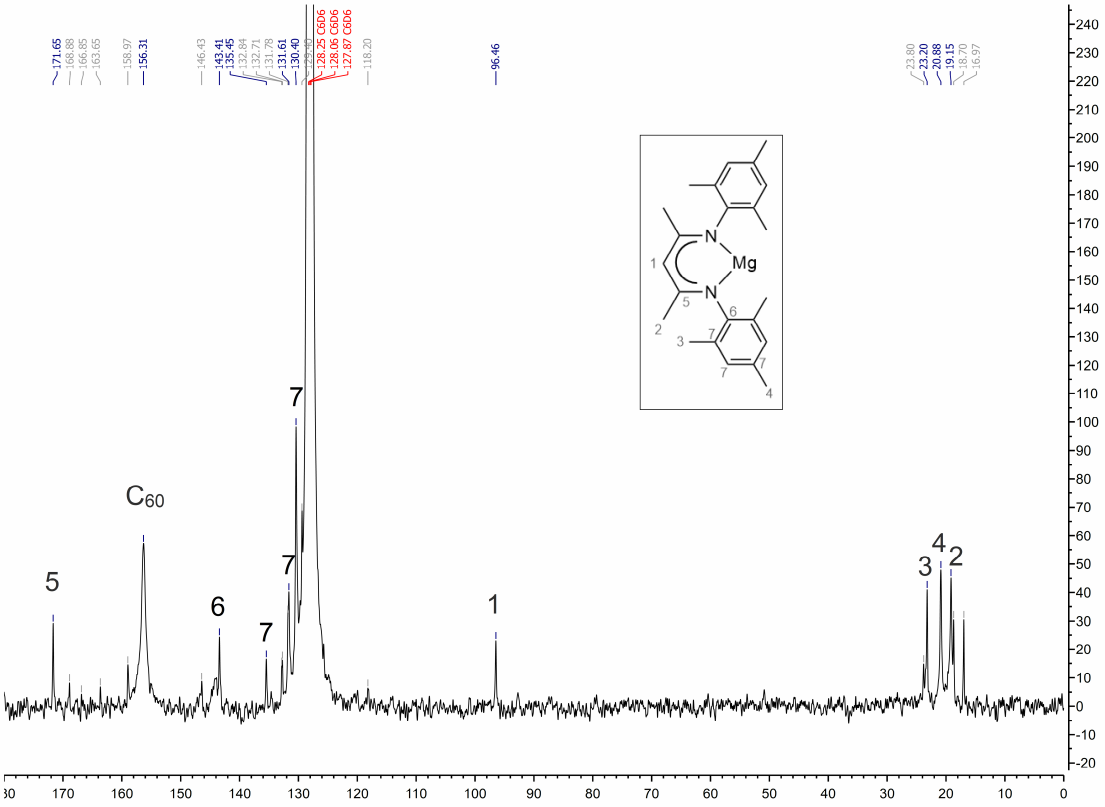

Figure S32. ^{13}C{^{1}H} NMR spectrum (125.7 MHz, 295 K) of [{(^{Mes}nacnac)Mg}$_2$C$_{60}$] **3c**.

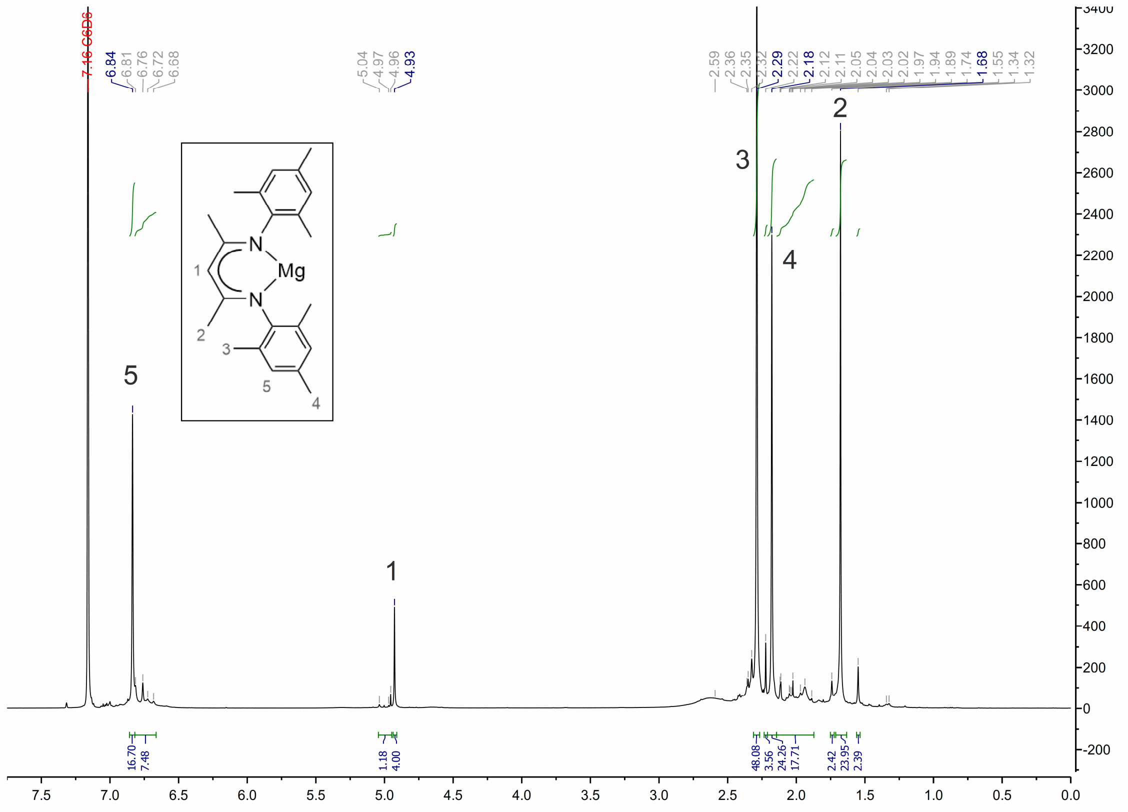

Figure S33. ^{1}H NMR spectrum (499.9 MHz, 295 K) of [{(^{Mes}nacnac)Mg}$_4$C$_{60}$] **4c**.

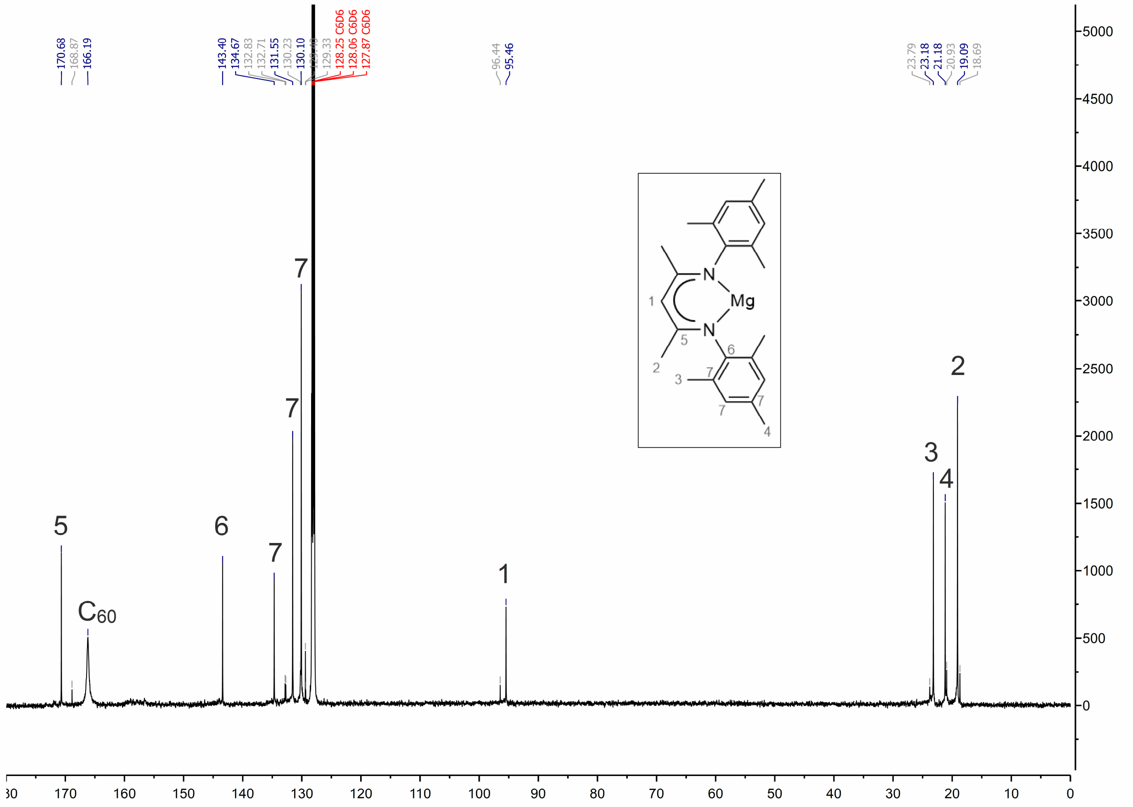

Figure S34. ^{13}C{^{1}H} NMR spectrum (125.7 MHz, 295 K) of [{(^{Mes}nacnac)Mg}$_4$C$_{60}$] **4c**.

2.2.4 NMR spectra of [{(^{Xyl}nacnac)Mg}$_n$C$_{60}$]

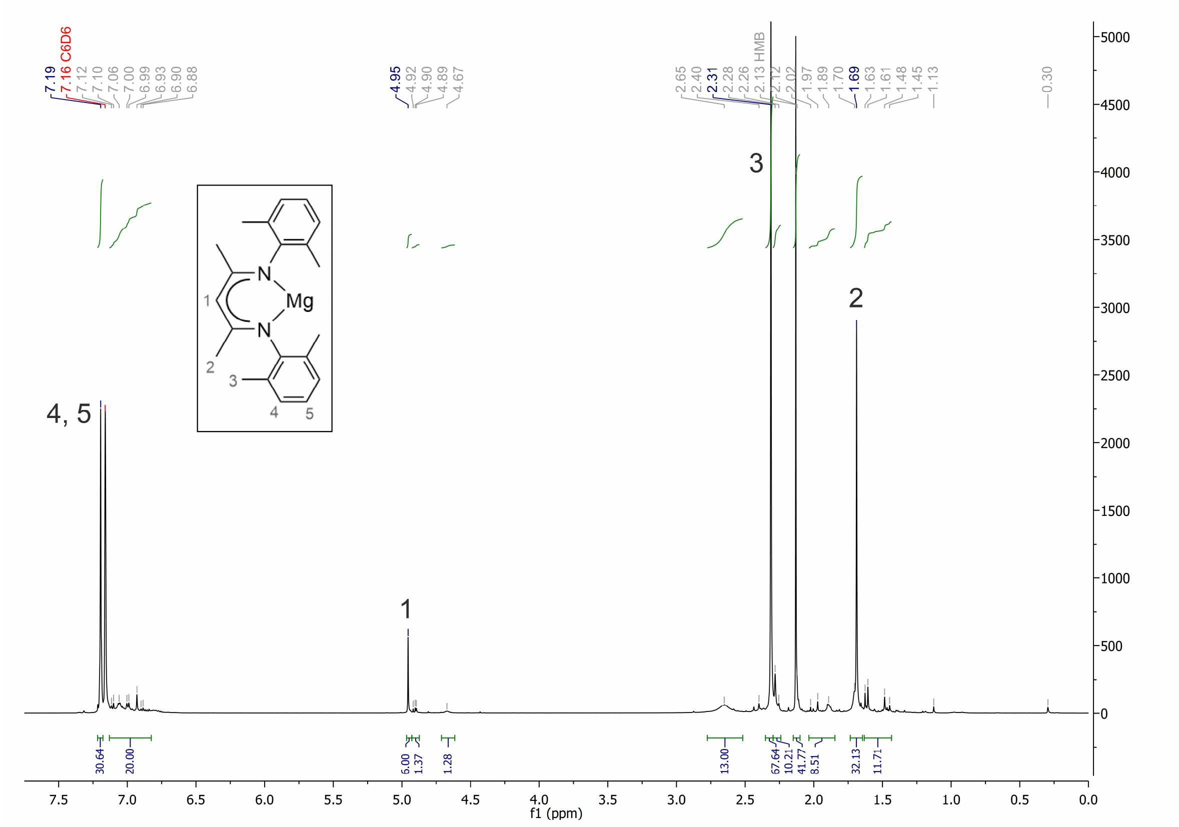

Figure S35. ^{1}H NMR spectrum (499.9 MHz, 295 K) of [{(^{Xyl}nacnac)Mg}$_6$C$_{60}$] **2d**.

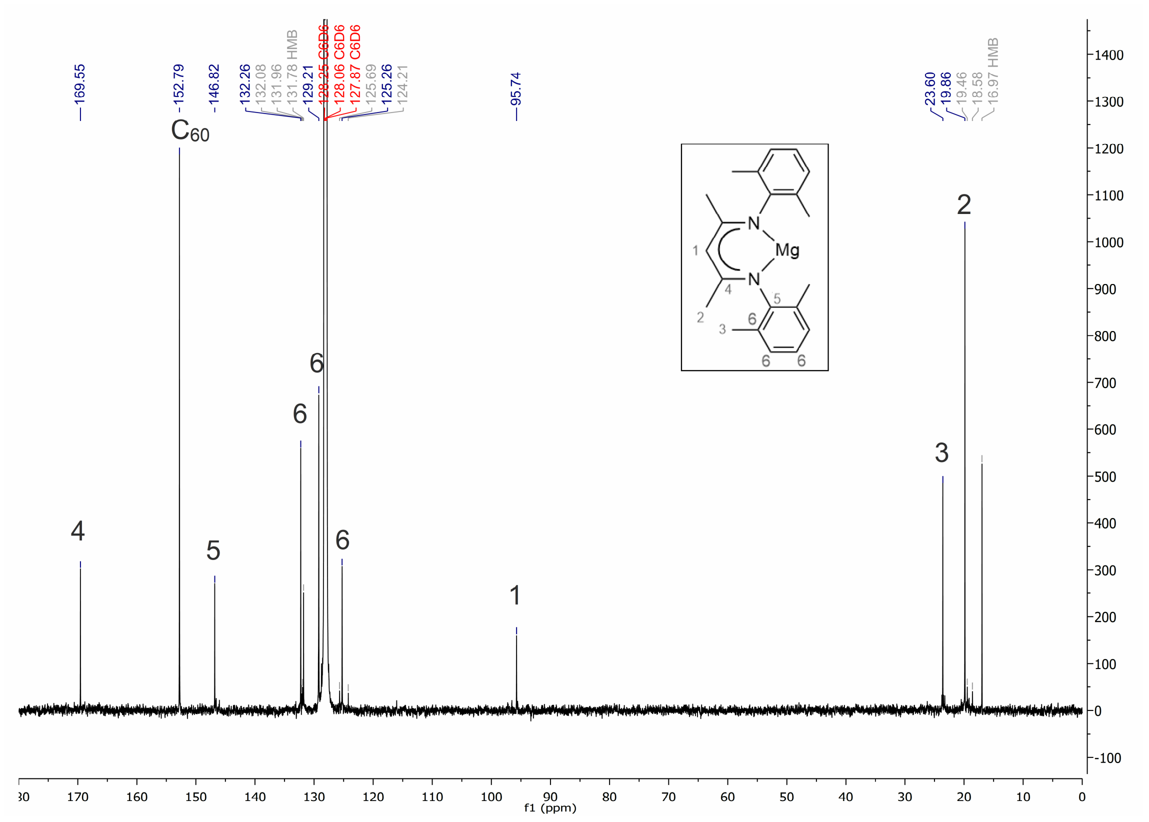

Figure S36. ^{13}C{^{1}H} NMR spectrum (125.7 MHz, 295 K) of [{(^{Xyl}nacnac)Mg}$_6$C$_{60}$] **2d**.

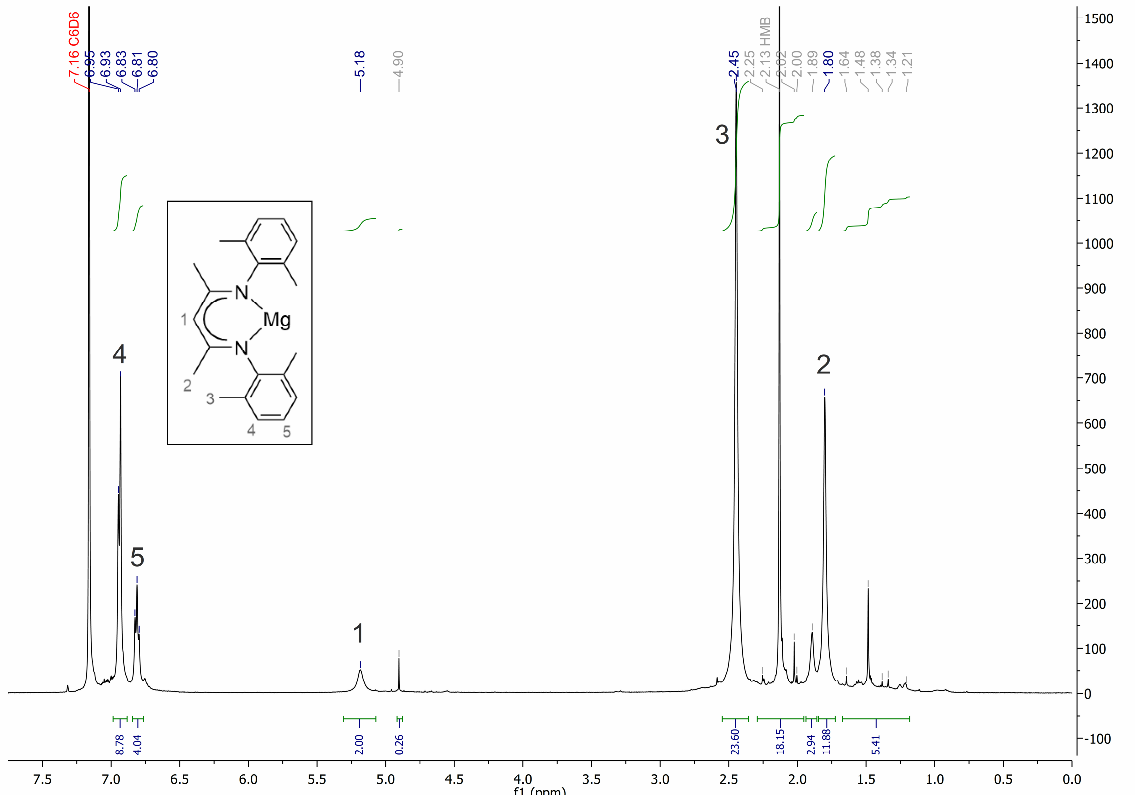

Figure S37. ^{1}H NMR spectrum (499.9 MHz, 295 K) of [{(^{Xyl}nacnac)Mg}$_2$C$_{60}$] **3d**. The broadening of the resonances is believed to result from the poor solubility of **3d**.

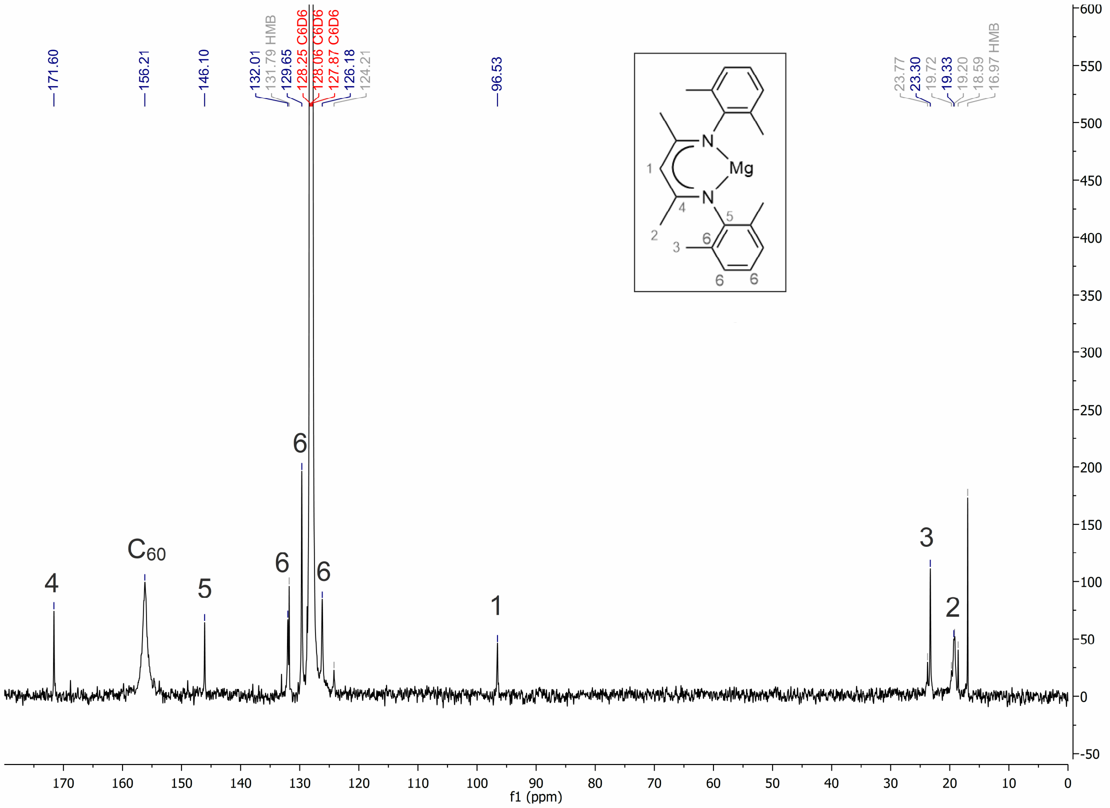

Figure S38. ^{13}C{^{1}H} (125.7 MHz, 295 K) NMR spectrum of [{(^{Xyl}nacnac)Mg}$_2$C$_{60}$] **3d**.

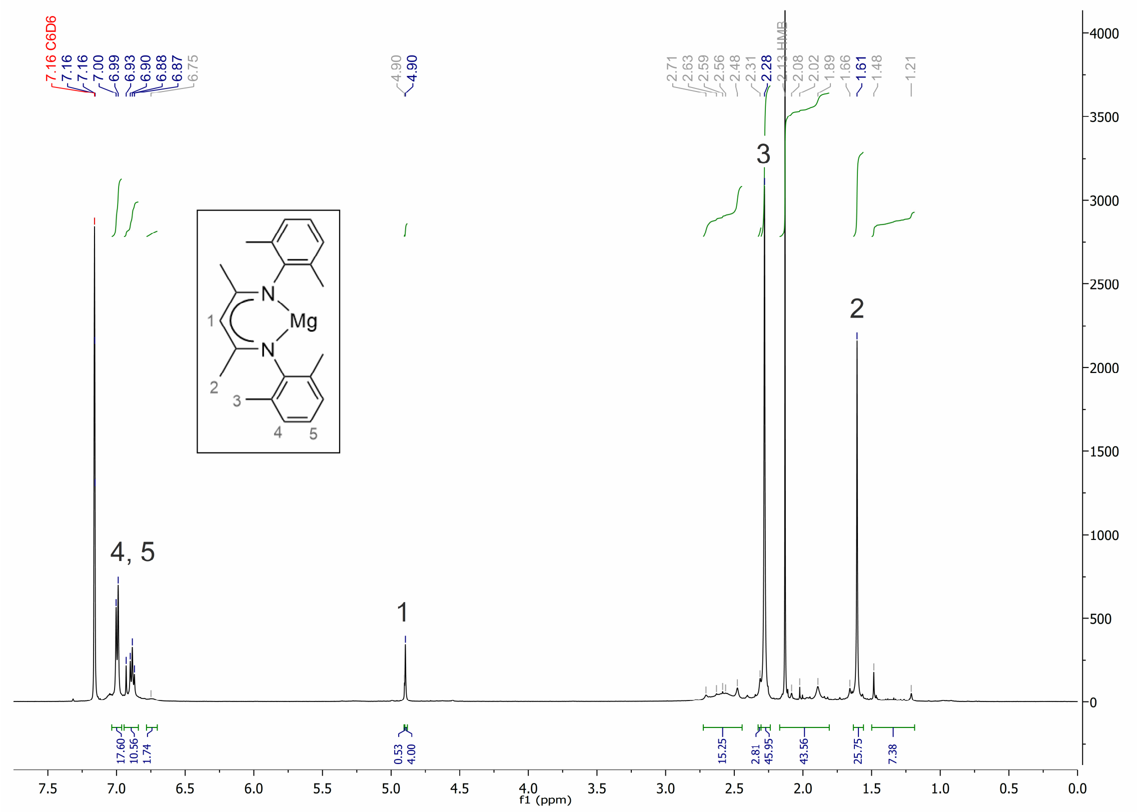

Figure S39. ^{1}H NMR spectrum (499.9 MHz, 295 K) of [{(^{Xyl}nacnac)Mg}$_4$C$_{60}$] **4d**.

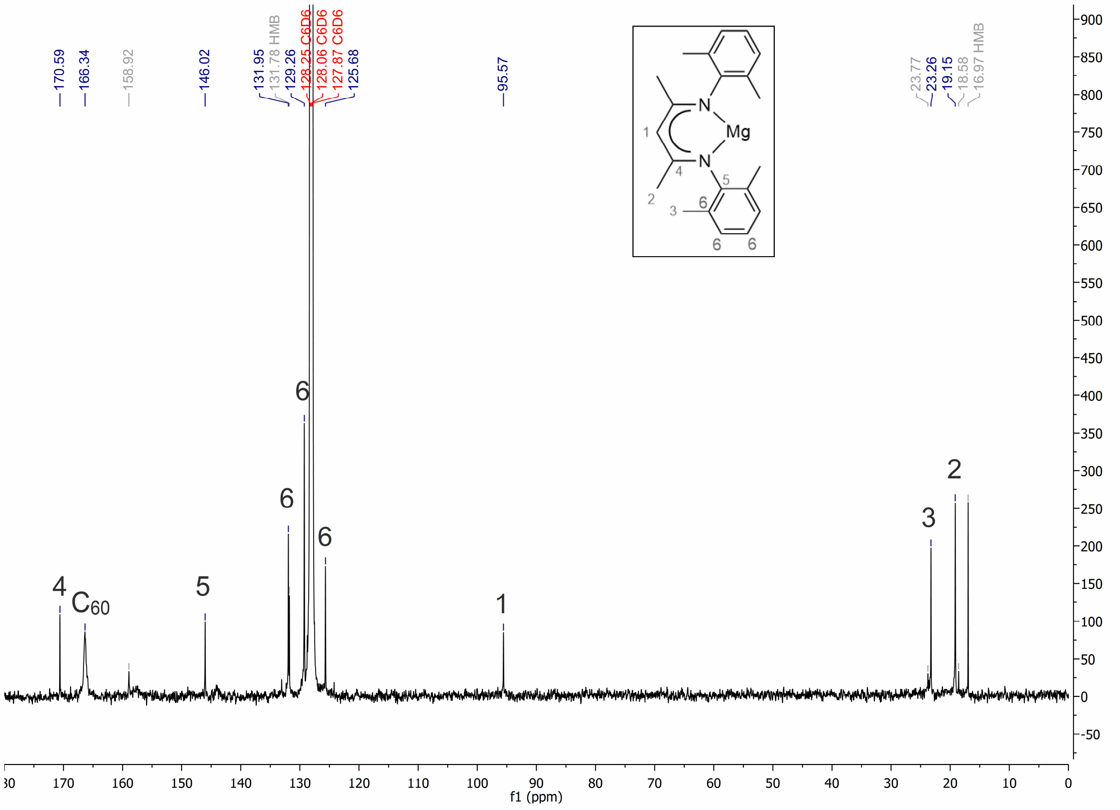

Figure S40. ^{13}C{^{1}H} NMR spectrum (125.7 MHz, 295 K) of [{(^{Xyl}nacnac)Mg}$_4$C$_{60}$] **4d**.

2.3 Titration experiments of [{(^{Ar}nacnac)Mg}$_2$] 1 with C_{60}

2.3.1 Titration reactions using [{(^{Dip}nacnac)Mg}$_2$] 1a

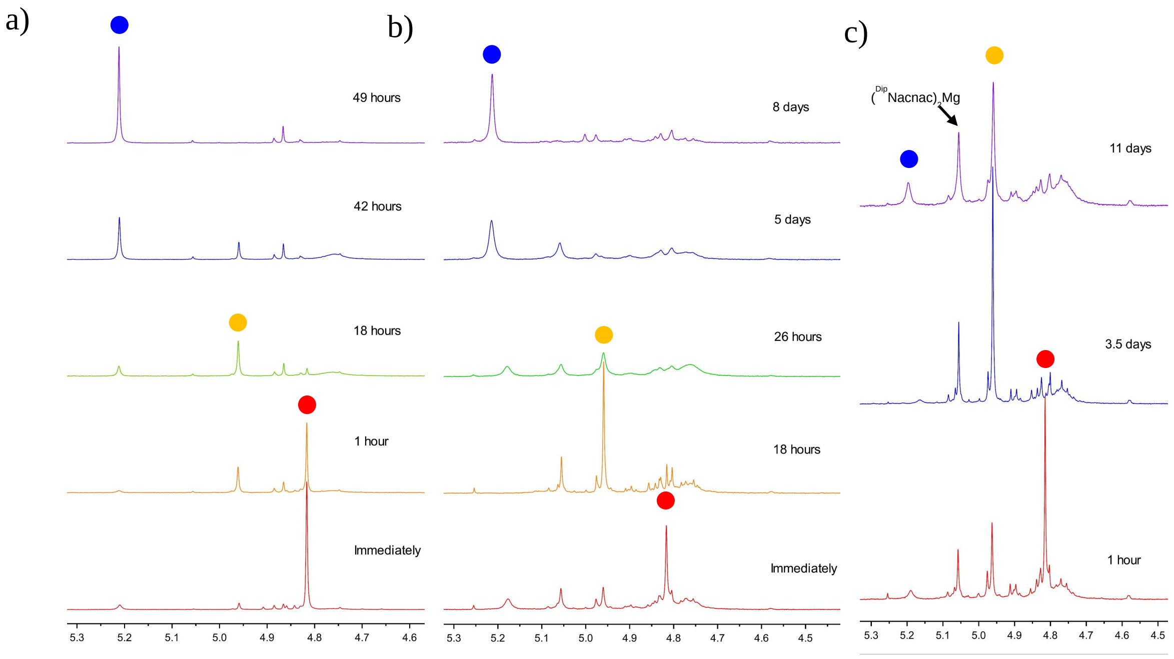

Figure S41. ^{1}H NMR spectroscopic time plots monitoring the a) first, c) second and c) third additions of 0.5 equivalents [{(^{Dip}Nacnac)Mg}$_2$] **1a** to C_{60}. Red dots: [{(^{Dip}Nacnac)Mg}$_2$] **1a**; blue dots: [{(^{Dip}Nacnac)Mg}$_2C_{60}$] **3a**; orange dots: [{(^{Dip}Nacnac)Mg}$_4C_{60}$] **4a**. For clarity, only the ligand backbone C-H environment is shown. Note that not all species are completely soluble during some stages of the reaction, especially at low **1a**:C_{60} ratios.

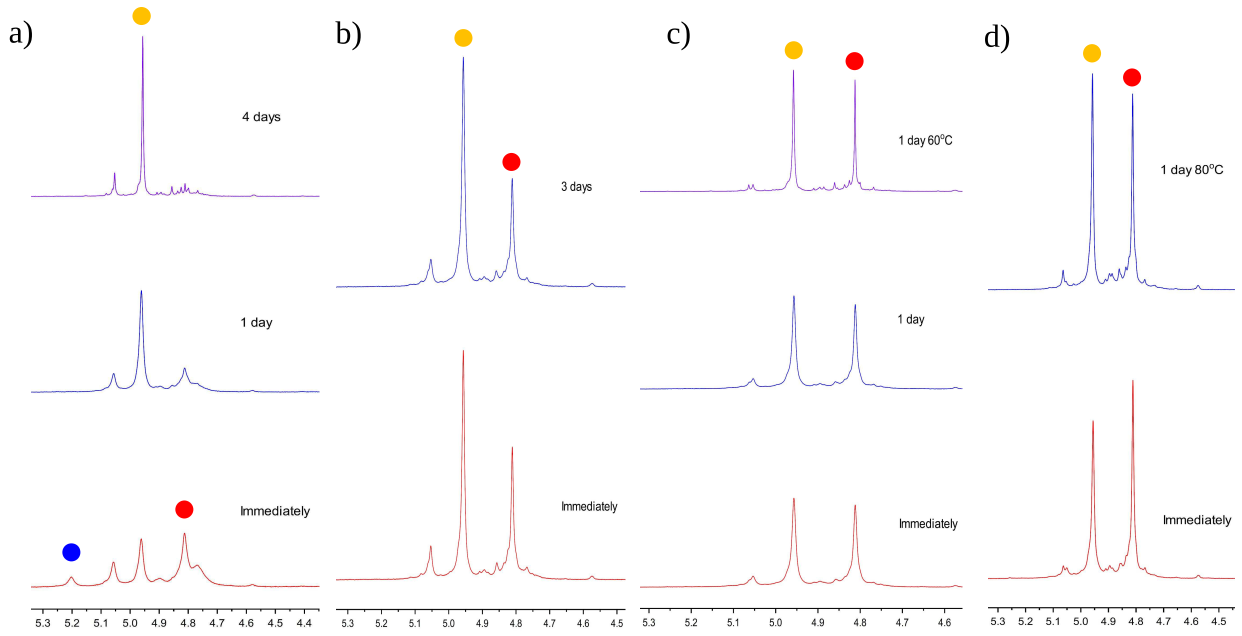

Figure S42. ^{1}H NMR spectroscopic time plots monitoring the a) fourth, c) fifth and c) sixth additions of 0.5 equivalents [{(^{Dip}Nacnac)Mg}$_2$] **1a** to C_{60}. Red dots: [{(^{Dip}Nacnac)Mg}$_2$] **1a**; blue dots: [{(^{Dip}Nacnac)Mg}$_2C_{60}$] **3a**; orange dots: [{(^{Dip}Nacnac)Mg}$_4C_{60}$] **4a**. For clarity, only the ligand backbone C-H environment is shown.

2.3.2 Titration reactions using [{(^{Dep}nacnac)Mg}$_2$] 1b

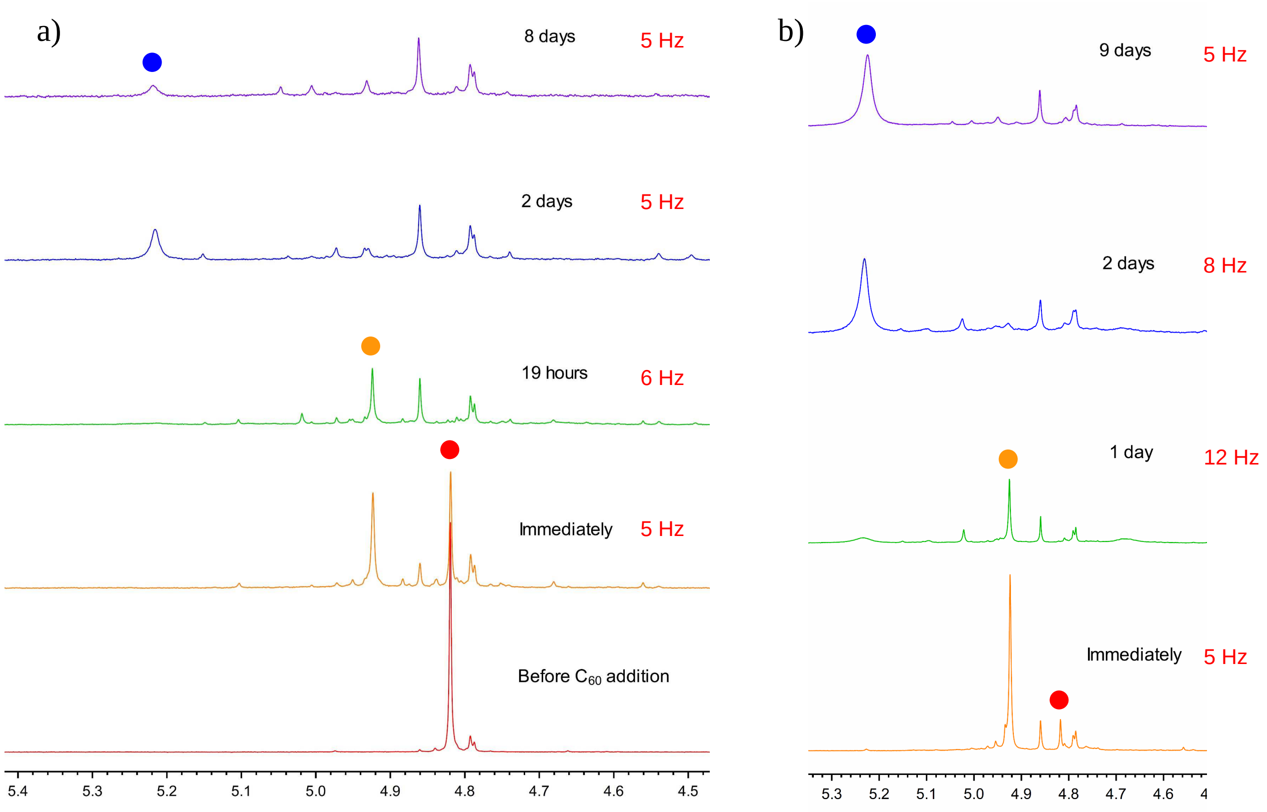

Figure S43. ^{1}H NMR spectroscopic time plots monitoring the a) first and b) second additions of 0.5 equivalents [{(^{Dep}Nacnac)Mg}$_2$] **1b** to C_{60}. Red dots: [{(^{Dep}Nacnac)Mg}$_2$] **1b**; blue dots: [{(^{Dep}Nacnac)Mg}$_2$$C_{60}$] **3b**; orange dots: [{(^{Dep}Nacnac)Mg}$_4$$C_{60}$] **4b**. For clarity, only the ligand backbone C-H environment is shown. Paramagnetic shifts arising from species in solution for each stage are listed beside the time signature in red (in Hz) for a relative measure of the solution magnetic moment. Note that not all species are completely soluble during some stages of the reaction, especially at low **1b**:C_{60} ratios.

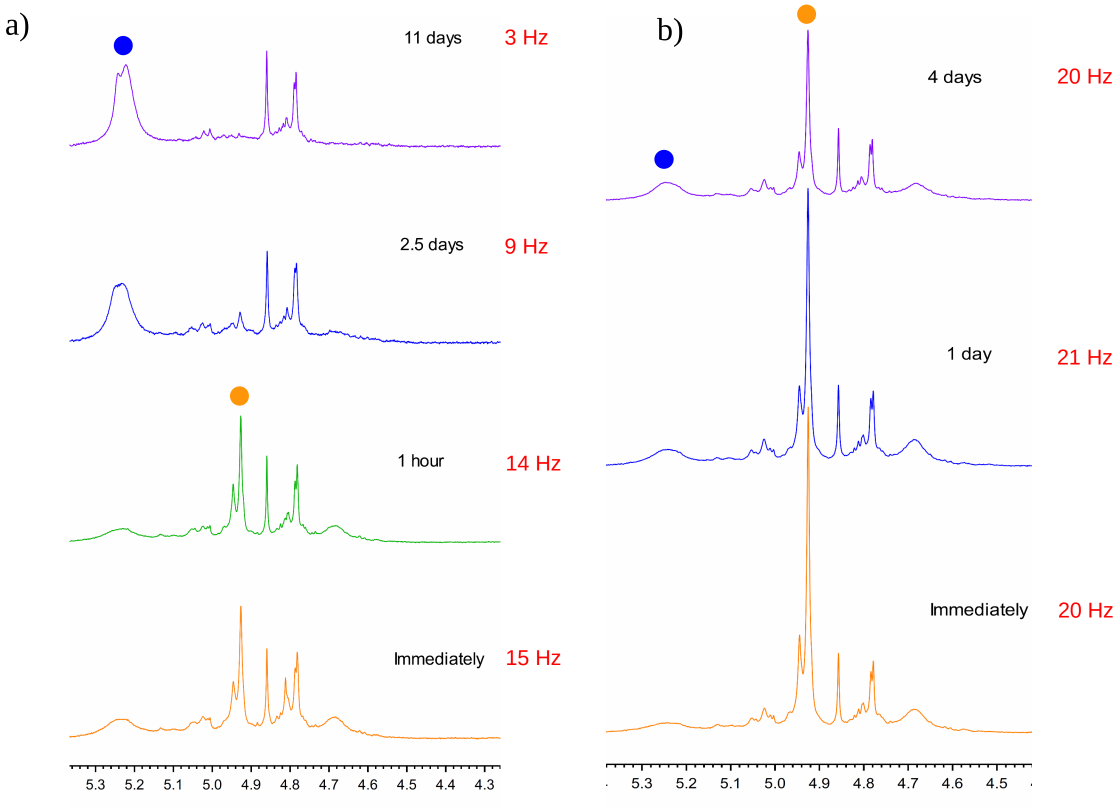

Figure S44. ^{1}H NMR spectroscopic time plots monitoring the a) third and b) fourth additions of 0.5 equivalents [{(^{Dep}Nacnac)Mg}$_2$] **1b** to C_{60}. Blue dots: [{(^{Dep}Nacnac)Mg}$_2C_{60}$] **3b**; orange dots: [{(^{Dep}Nacnac)Mg}$_4C_{60}$] **4b**. For clarity, only the ligand backbone C-H environment is shown. For clarity, only the ligand backbone C-H environment is shown. Paramagnetic shifts arising from species in solution for each stage are listed beside the time signature in red (in Hz) for a relative measure of the solution magnetic moment.

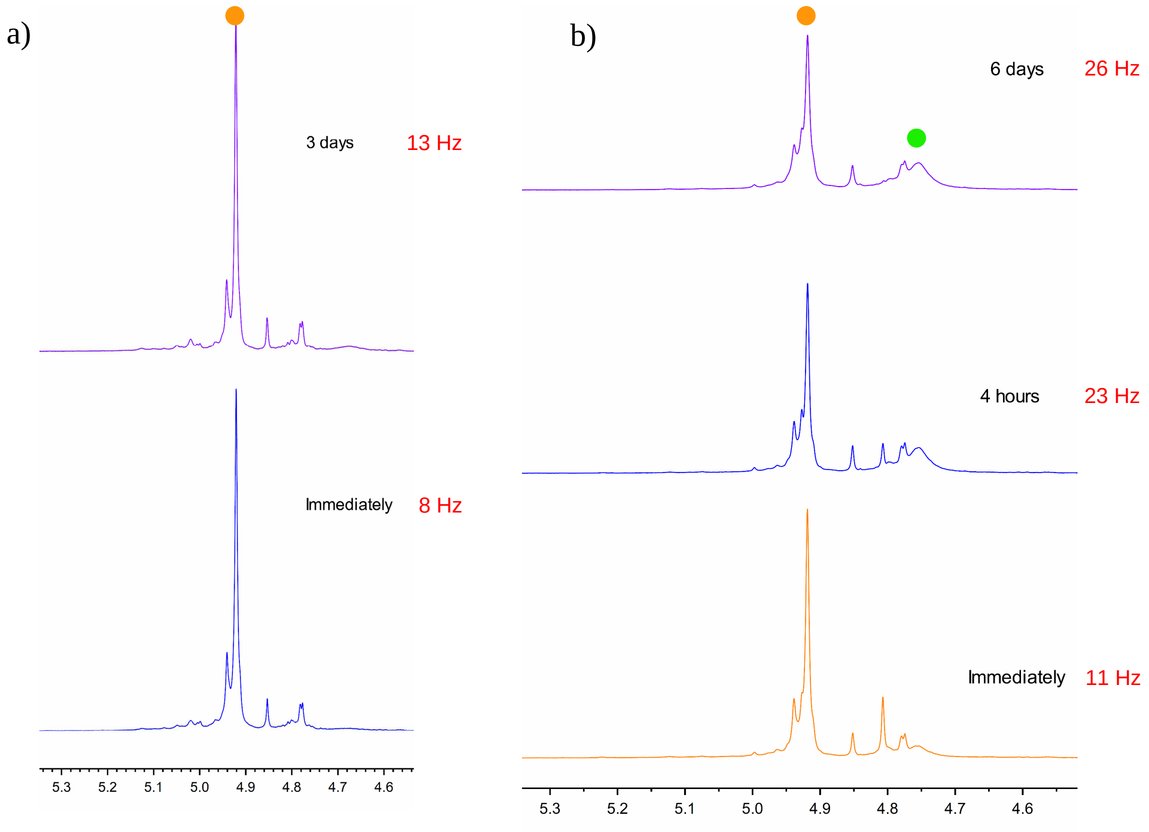

Figure S45. ^{1}H NMR spectroscopic time plots monitoring the a) fifth and b) sixth additions of 0.5 equivalents [{(^{Dep}Nacnac)Mg}$_2$] **1b** to C_{60}. Orange dots: [{(^{Dep}nacnac)Mg}$_4C_{60}$] **4b**; green dots: putative [{(^{Dep}nacnac)Mg}$_5C_{60}$] **5b**. For clarity, only the ligand backbone C-H environment is shown. For clarity, only the ligand backbone C-H environment is shown. Paramagnetic shifts arising from species in solution for each stage are listed beside the time signature in red (in Hz) for a relative measure of the solution magnetic moment.

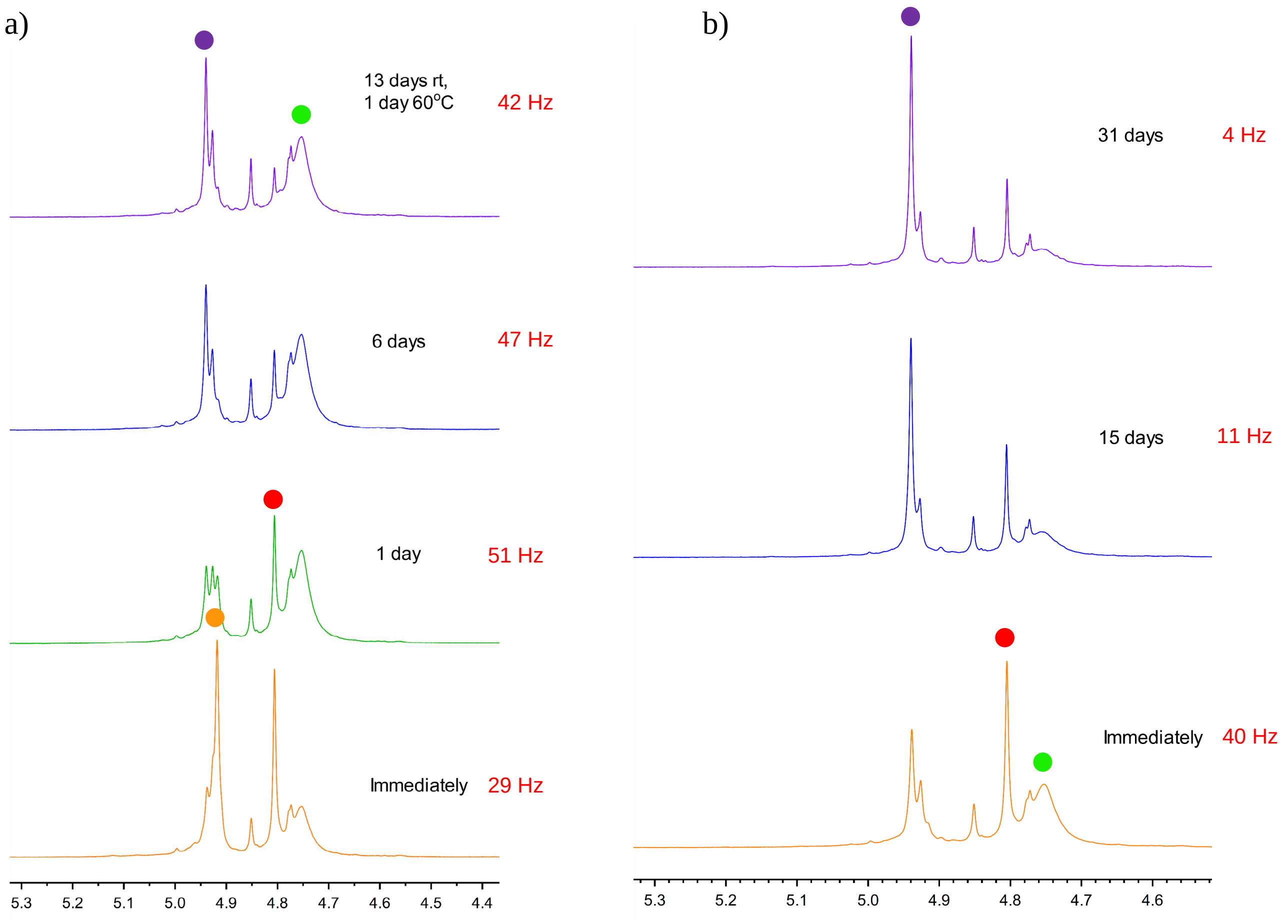

Figure S46. ^{1}H NMR spectroscopic time plots monitoring the a) seventh and b) eighth additions of 0.5 equivalents [{(^{Dep}Nacnac)Mg}$_2$] **1b** to C_{60}. Red dots: [{(^{Dep}Nacnac)Mg}$_2$] **1b**; purple dots: [{(^{Dep}nacnac)Mg}$_6$C$_{60}$] **2b**; orange dots: [{(^{Dep}nacnac)Mg}$_4$C$_{60}$] **4b**; green dots: [{(^{Dep}nacnac)Mg}$_5$C$_{60}$] **5b**. For clarity, only the ligand backbone C-H environment is shown. For clarity, only the ligand backbone C-H environment is shown. Paramagnetic shifts arising from species in solution for each stage are listed beside the time signature in red (in Hz) for a relative measure of the solution magnetic moment. The paramagnetic shift and the solution magnetic moment of the mixture appears to mainly depend on the concentratiuon of [{(^{Dep}nacnac)Mg}$_5$C$_{60}$] **5b**, green dot.

2.3.3 Titration reactions using [{(^{Mes}nacnac)Mg}$_2$] 1c

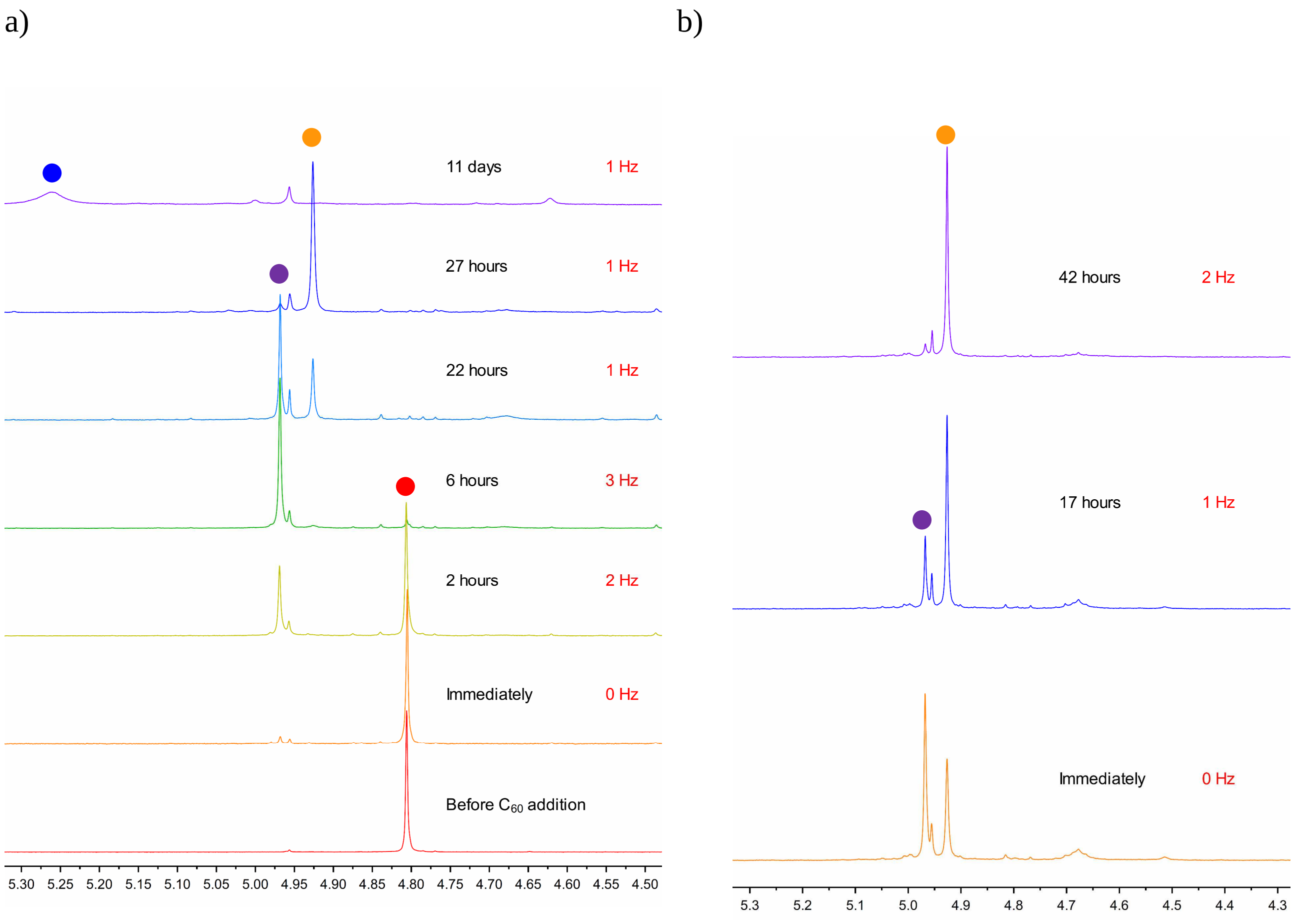

Figure S47. ^{1}H NMR spectroscopic time plots monitoring the a) first and b) second additions of 0.5 equivalents [{(^{Mes}Nacnac)Mg}$_2$] **1c** to C_{60}. Red dot: [{(^{Mes}Nacnac)Mg}$_2$] **1c**; purple dots: [{(^{Mes}nacnac)Mg}$_6$C$_{60}$] **2c**; orange dots: [{(^{Mes}nacnac)Mg}$_4$C$_{60}$] **4c**; blue dot: [{(^{Mes}nacnac)Mg}$_2$C$_{60}$] **3c**. For clarity, only the ligand backbone C-H environment is shown. For clarity, only the ligand backbone C-H environment is shown. Paramagnetic shifts arising from species in solution for each stage are listed beside the time signature in red (in Hz) for a relative measure of the solution magnetic moment. Note that not all species are completely soluble during some stages of the reaction, especially at low **1c**:C_{60} ratios.

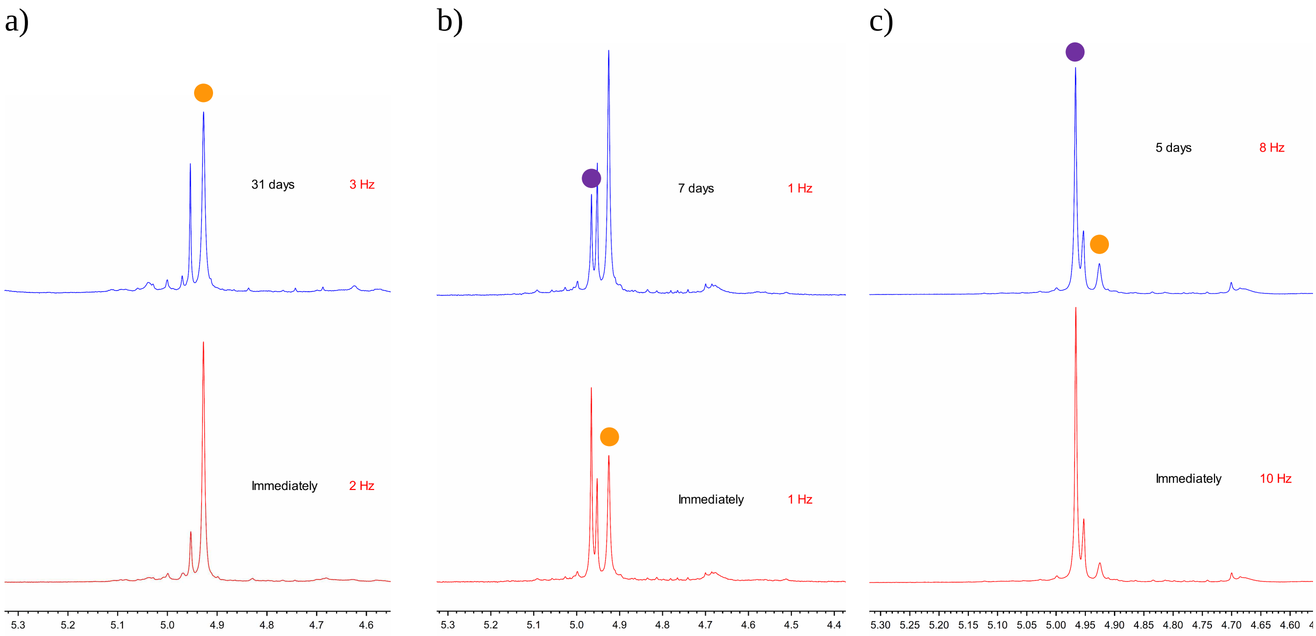

Figure S48. ^{1}H NMR spectroscopic time plots monitoring the a) third, b) fourth and c) fifth additions of 0.5 equivalents [{(^{Mes}Nacnac)Mg}$_2$] **1c** to C_{60}. Purple dots: [{(^{Mes}nacnac)Mg}$_6$C$_{60}$] **2c**; orange dots: [{(^{Mes}nacnac)Mg}$_4$C$_{60}$] **4c**. For clarity, only the ligand backbone C-H environment is shown. For clarity, only the ligand backbone C-H environment is shown. Paramagnetic shifts arising from species in solution for each stage are listed beside the time signature in red (in Hz) for a relative measure of the solution magnetic moment.

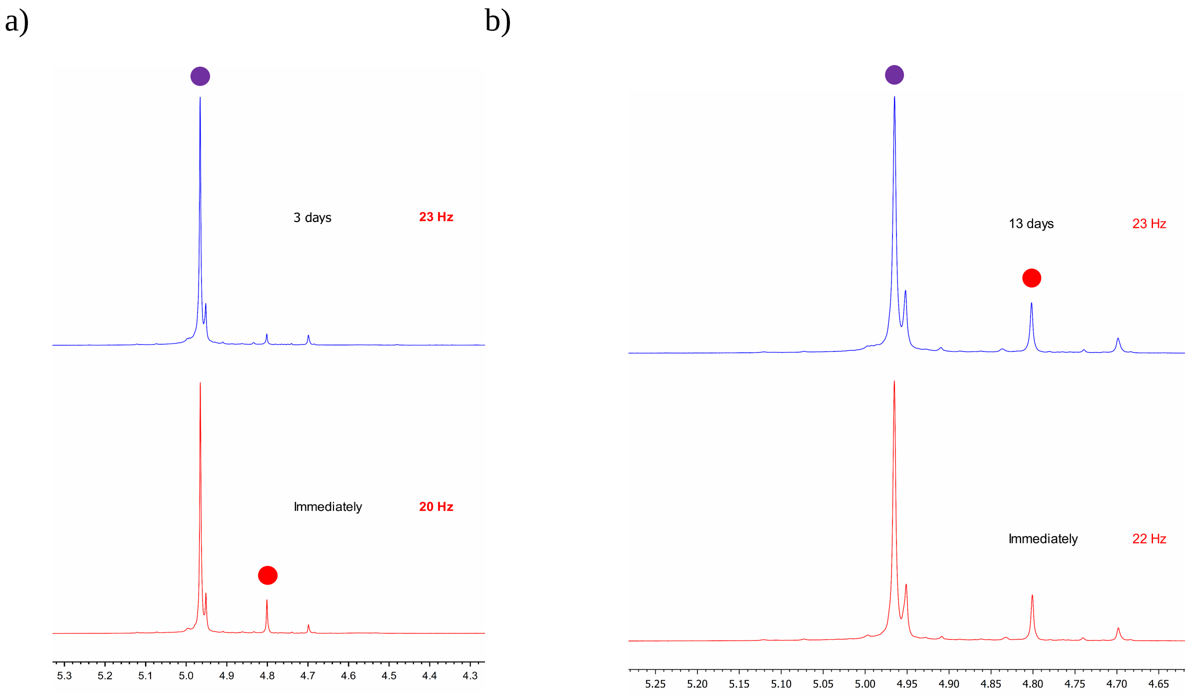

Figure S49. ^{1}H NMR spectroscopic time plots monitoring the a) sixth and b) seventh additions of 0.5 equivalents [{(^{Mes}Nacnac)Mg}$_2$] **1c** to C_{60}. Red dots: [{(^{Mes}Nacnac)Mg}$_2$] **1c**; purple dots: [{(^{Mes}nacnac)Mg}$_6$$C_{60}$] **2c**. Paramagnetic shifts arising from species in solution for each stage are listed eside the time signature in red, given in Hz.

2.4 Reaction of [{(^{Mes}nacnac)Mg}$_6$C$_{60}$] 2c with [(^{Xyl}nacnac)Li]

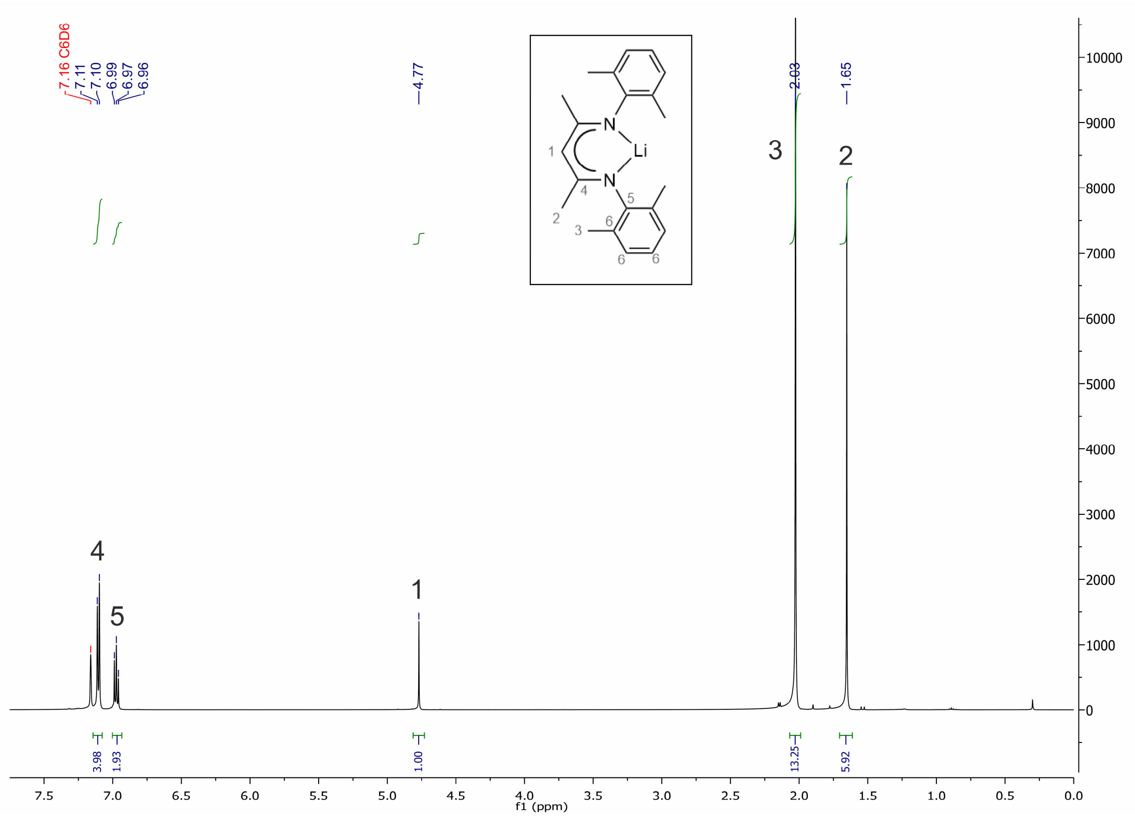

Figure S50. ^{1}H NMR spectrum (499.9 MHz, 295K) of isolated [(^{Xyl}Nacnac)Li].

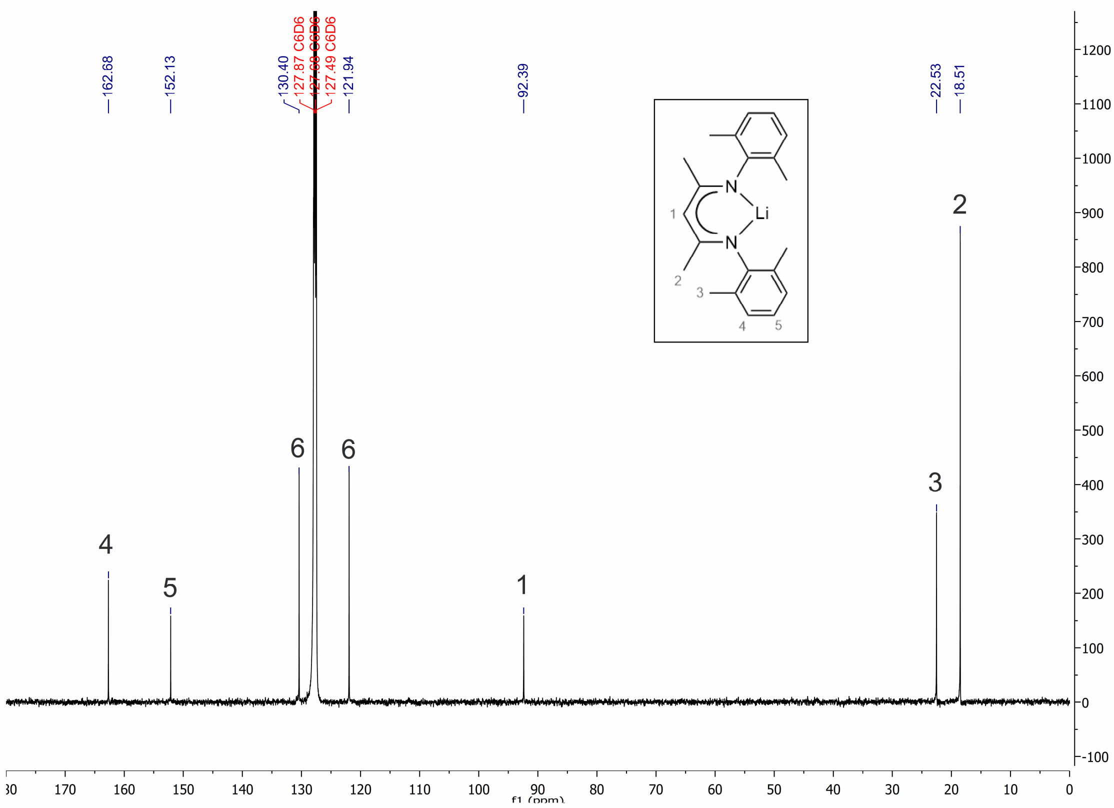

Figure S51. ^{13}C{^{1}H} NMR spectrum (125.7 MHz, 295K) of isolated [(^{Xyl}Nacnac)Li].

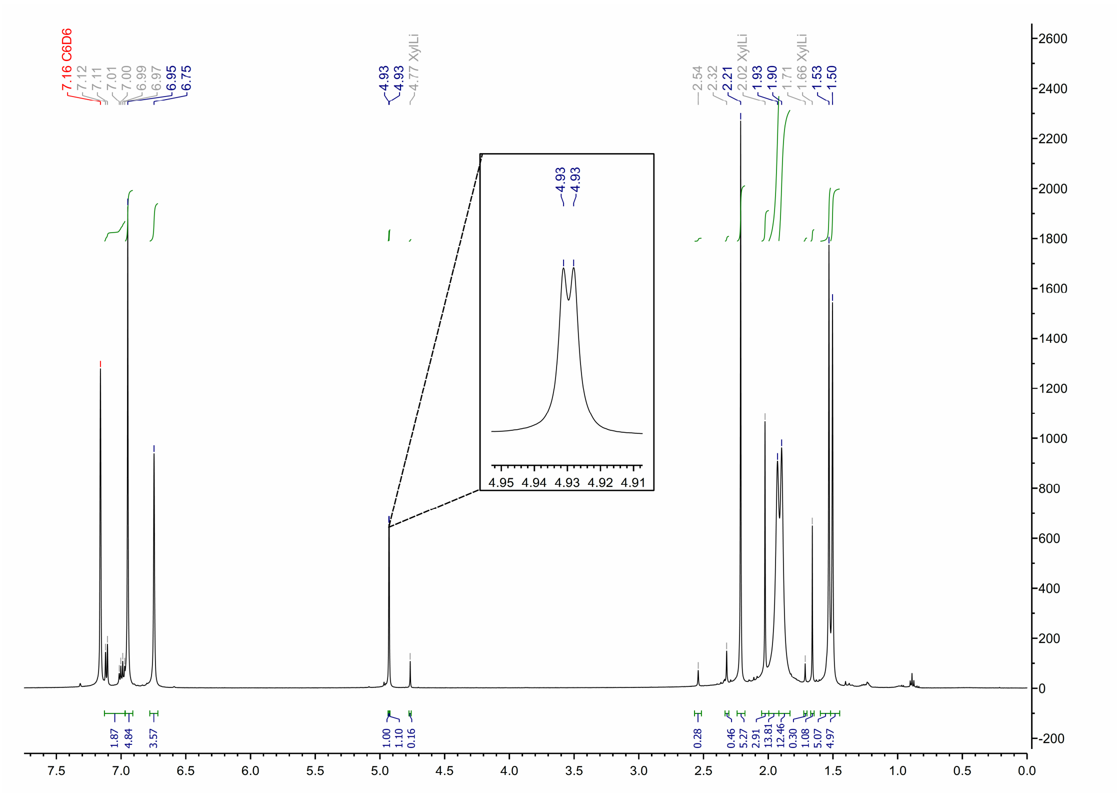

Figure S52. ^{1}H NMR spectrum (499.9 MHz, 295 K) of the reaction mixture of [{(^{Mes}nacnac)Mg}$_6$C$_{60}$] **2c** and six equivalents of [(^{Xyl}Nacnac)Li] taken immediately after addition.

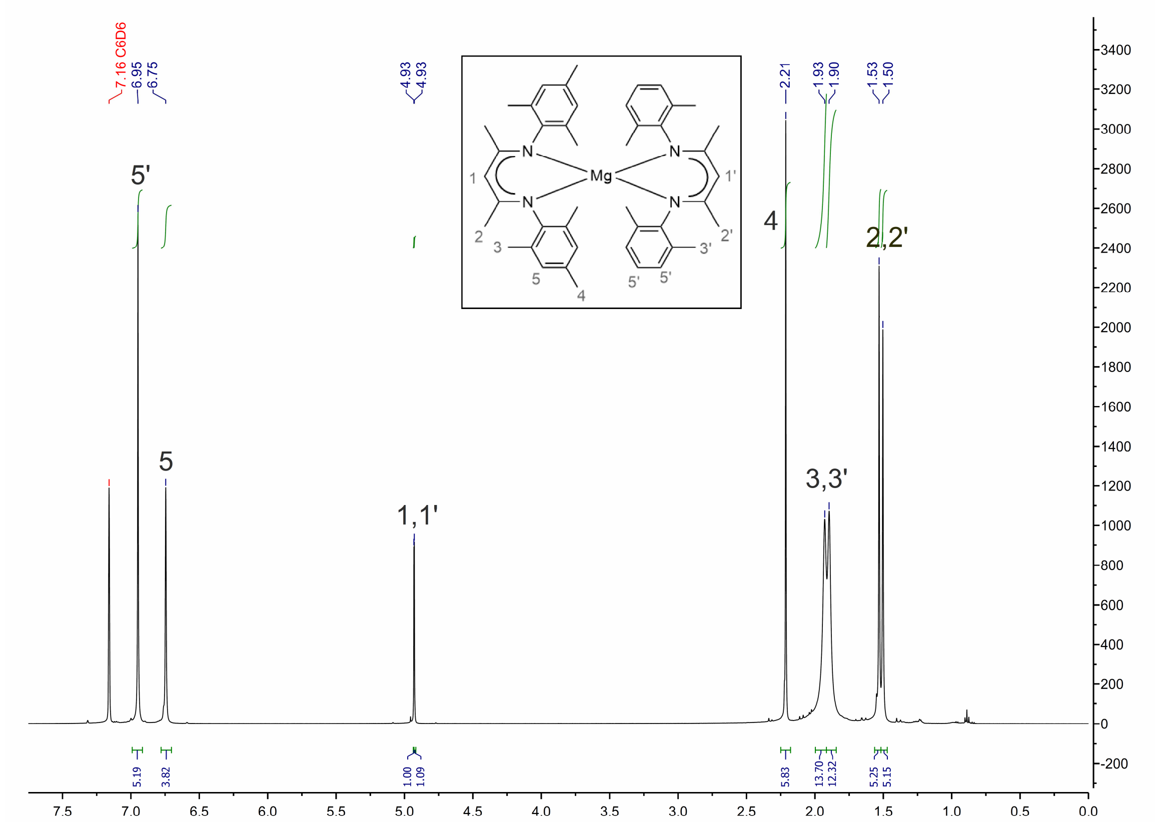

Figure S53. ^{1}H NMR spectrum (499.9 MHz, 295 K) of [(^{Mes}Nacnac)Mg(^{Xyl}Nacnac)] **6**, the product of the reaction of [{(^{Mes}nacnac)Mg}$_6$C$_{60}$] **2c** with six equivalents of [(^{Xyl}Nacnac)Li].

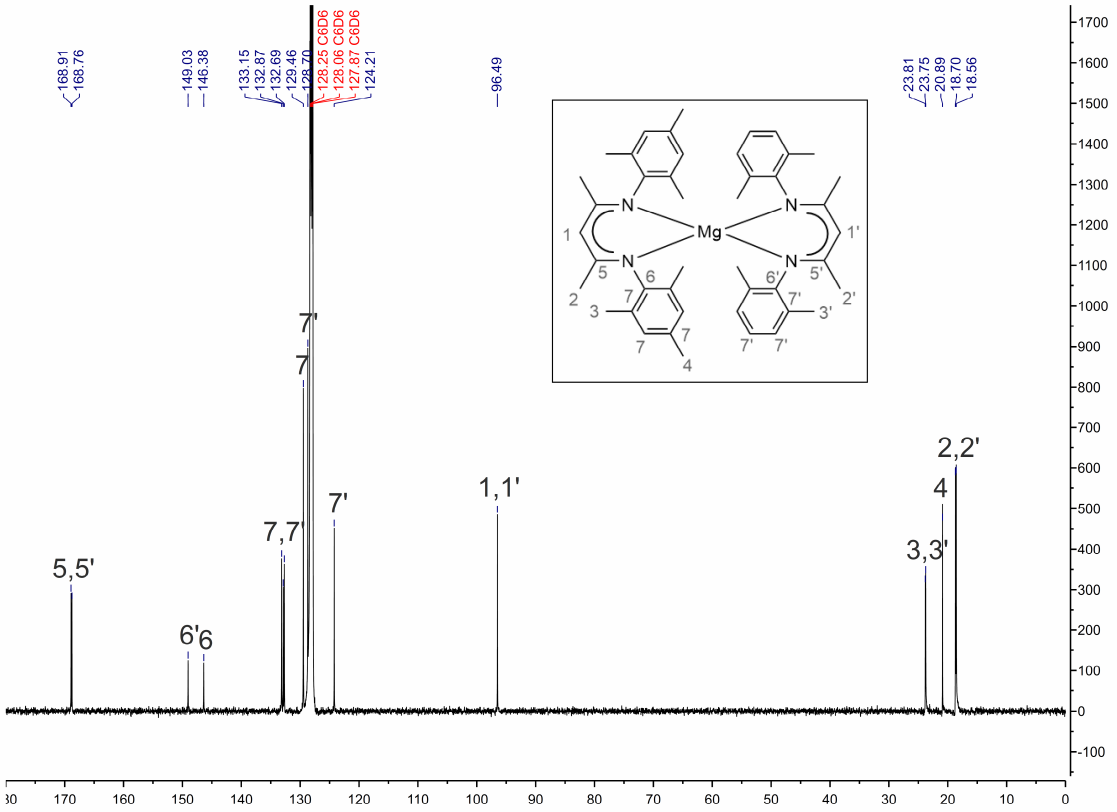

Figure S54. $^{13}C\{^{1}H\}$ NMR spectrum (125.7 MHz, 295 K) of [(^{Mes}Nacnac)Mg(^{Xyl}Nacnac)] **6**, the product of the reaction of [{(^{Mes}nacnac)Mg}$_6$C$_{60}$] **2c** with six equivalents of [(^{Xyl}Nacnac)Li].

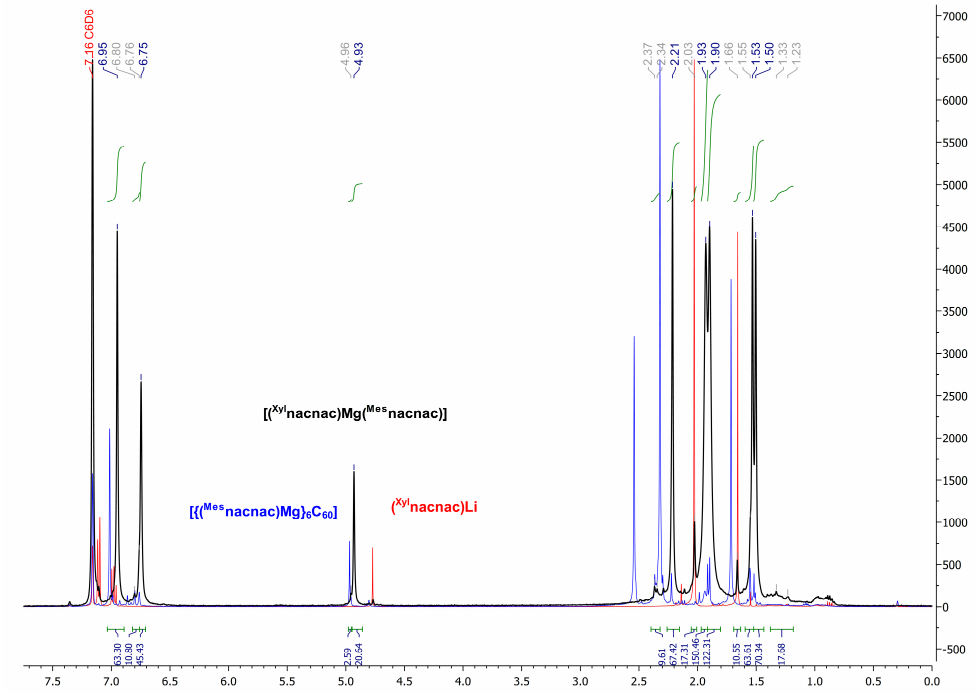

Figure S55. Superimposed ^{1}H NMR spectra (400.1 MHz, 295 K) of starting materials [{(^{Mes}nacnac)Mg}$_6$C$_{60}$] **2c** (blue) and [(^{Xyl}Nacnac)Li] (red), and product [(^{Mes}Nacnac)Mg(^{Xyl}Nacnac)] **6** (black).

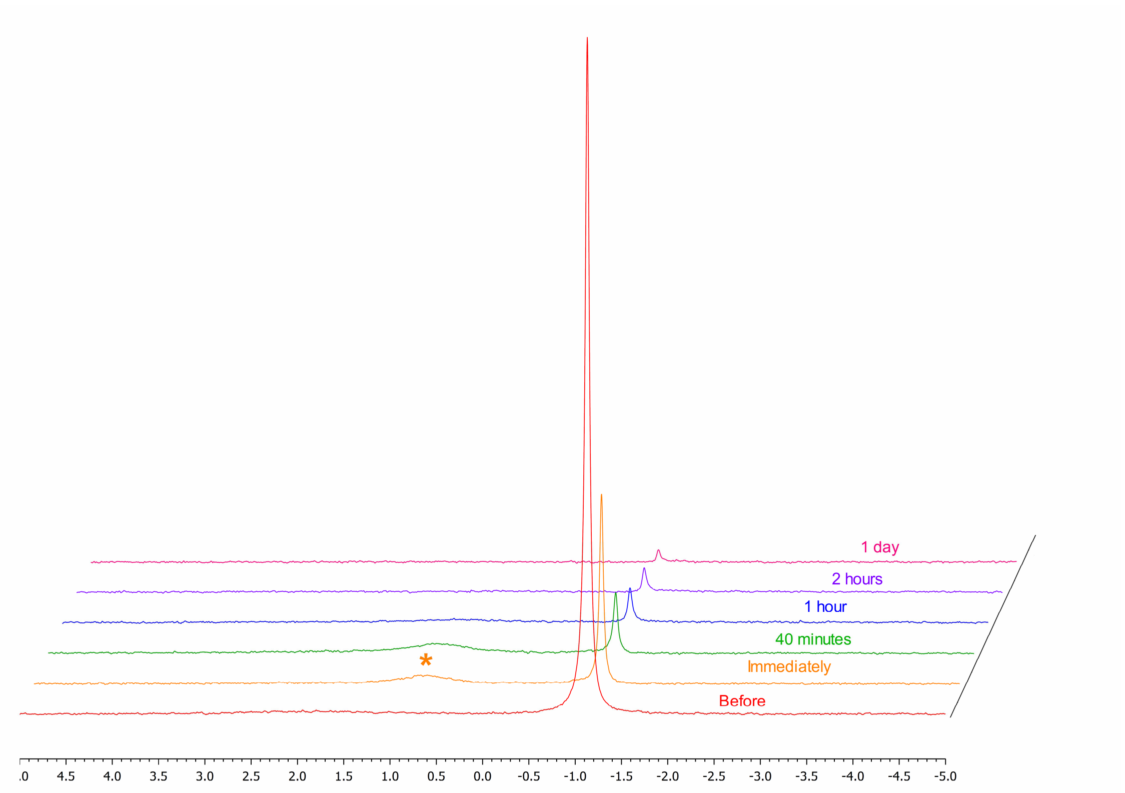

Figure S56. Stacked ^{7}Li NMR spectra (155.5 MHz, 295 K) of the reaction between [{(^{Mes}nacnac)Mg}$_6$C$_{60}$] **2c** with six equivalents of [(^{Xyl}Nacnac)Li] in deuterated benzene showing the consumption of [(^{Xyl}Nacnac)Li]. An intermediate with a broad resonance (*, 0.8 ppm) formed that could be putative Li$_6$C$_{60}$ or a closely related material before precipitation is completed.

3 X-ray crystallography

Suitable crystals were mounted in silicone oil and were measured using either a Rigaku FR-X Ultrahigh brilliance Microfocus RA generator/confocal optics with XtaLAB P200 diffractometer (Mo Kα radiation) or a Rigaku MM-007HF High Brilliance RA generator/confocal optics with XtaLAB P100 or P200 diffractometer (Cu Kα radiation). Data for all compounds analysed were collected using CrystalClear.[7] Data were processed (including correction for Lorentz, polarization and absorption) using either CrystalClear[7] or CrysAlisPro.[8] Structures were solved by direct (SIR2011),[9] charge-flipping (Superflip),[10] or dual-space (SHELXT-2014/5)[11] methods and refined by full-matrix least-squares against F^2 using SHELXL-2018/3.[12] All non-hydrogen atoms were refined anisotropically except in selected cases as described below. Hydrogen atoms were placed in calculated positions (riding model). In some cases, severely disordered solvent of crystallisation (hydrocarbons) was removed using the SQUEEZE routine.[13] The removed solvent electron-density is not included in the formula, density, $F(000)$ etc. A description of the individual SQUEEZE details including the volume, number of electrons removed and some residual electron density peaks etc are included in the CIF-files. All calculations were performed using either the CrystalStructure[14] or Olex2[15] interface, except for SQUEEZE, which was accessed via PLATON.[16] Crystallographic data is summarized in Table S1. Details on individual crystal structure determinations and refinements are given below. Further experimental and refinement details are given in the CIF-files. CCDC 1914895-1914903 contains the supplementary crystallographic data for this paper. These data can be obtained free of charge via https://www.ccdc.cam.ac.uk/structures/.

Table S1 Crystallographic data.

Compound reference	**1d**	**2b'**	**2c'**	**2c''**	**2d'**
Chemical formula	$C_{42}H_{50}Mg_2N_4$	$C_{210}H_{198}Mg_6N_{12}$	$C_{240}H_{216}Mg_6N_{12}$	$C_{198}H_{174}Mg_6N_{12}$	$C_{228}H_{192}Mg_6N_{12}$
Formula weight	659.49	3035.78	3414.26	2867.46	3245.94
Temperature/K	173(2)	173(2)	125(2)	173(2)	125(2)
Radiation type	Mo	Cu	Cu	Cu	Cu
Wavelength/Å	0.71073	1.54187	1.54187	1.54187	1.54184
Crystal system	Monoclinic	Monoclinic	Monoclinic	Monoclinic	Triclinic
Space group	*C*2/*c*	$P2_1/n$	$P2_1/n$	$P2_1$	*P*-1
a/Å	21.1207(6)	26.4392(3)	18.19780(19)	18.2173(8)	17.1410(3)
b/Å	8.4930(2)	23.3427(3)	33.3681(4)	30.2552(11)	17.1631(3)
c/Å	23.5701(8)	27.4336(4)	30.8873(4)	33.8828(13)	17.4199(3)
α/°	90	90	90	90	117.7300(16)
β/°	108.991(3)	93.4861(12)	90.8983(10)	102.678(4)	94.6116(13)
γ/°	90	90	90	90	100.4360(13)
Unit cell volume/$Å^3$	3997.8(2)	16899.7(4)	18753.3(4)	18219.8(13)	4380.62(15)
No. of formula units per unit cell, *Z*	4	4	4	4	1
Density (calc)/ Mg/m^3	1.096	1.193	1.209	1.045	1.230
Absorption coefficient, μ/mm^{-1}	0.092	0.729	0.714	0.652	0.740
F(000)	1416	6456	7248	6072	1716
Theta range/°	1.827 to 29.041	2.487 to 68.308	2.648 to 75.521	2.486 to 68.682	3.882 to 75.505
Reflections collected	25260	176295	223767	192610	46776
Independent reflections	4657	30656	38226	63773	17229
R_{int}	0.0422	0.0734	0.0545	0.1371	0.0261
Completeness (to theta)/%	100 (25.242°)	99.5 (67.687°)	99.9 (67.687°)	98.7 (67.687°)	98.0 (67.684°)
Data / restraints / parameter	4657 / 0 / 223	30656 / 94 / 2194	38226 / 0 / 2371	63773 / 1 / 3985	17229 / 0 / 1126
Goodness of fit on F^2	1.066	1.050	1.017	0.954	1.020
Final R_1 values ($I > 2\sigma(I)$)	0.0457	0.0831	0.0469	0.0894	0.0511
Final $wR(F^2)$ values ($I > 2\sigma(I)$)	0.1379	0.2379	0.1224	0.2212	0.1438
Final R_1 values (all data)	0.0532	0.1021	0.0596	0.1505	0.0534
Final $wR(F^2)$ values (all data)	0.1436	0.2503	0.1313	0.2648	0.1465
Largest diff. peak and hole/$e \cdot Å^{-3}$	0.278 and -0.248	0.556 and -0.332	0.356 and -0.348	0.661 and -0.363	0.378 and -0.435
Absolute structure parameter	-	-	-	0.42(4)	-
CCDC number	1914895	1914896	1914897	1914898	1914899

Table S1 continued Crystallographic data.

Compound reference	**3a'**	**3a''**	**3b'**	**4a'**
Chemical formula	$C_{142}H_{106}Mg_2N_4$	$C_{133}H_{97}Mg_2N_4$	$C_{110}H_{66}Mg_2N_4$	$C_{176}H_{164}Mg_4N_8$
Formula weight	1917.04	1799.87	1492.37	2488.51
Temperature/K	173(2)	173(2)	173(2)	173(2)
Radiation type	Cu	Cu	Cu	Cu
Wavelength/Å	1.54187	1.54187	1.54184	1.54187
Crystal system	Triclinic	Triclinic	Monoclinic	Monoclinic
Space group	*P*-1	*P*-1	$P2_1/n$	*C*2/*c*
a/Å	14.5468(7)	14.551(3)	15.8575(5)	34.388(2)
b/Å	14.6808(4)	14.651(2)	20.1901(4)	12.2868(7)
c/Å	24.3760(7)	22.814(4)	25.5394(8)	33.068(2)
α/°	80.200(2)	96.351(3)	90	90
β/°	79.333(3)	103.923(5)	98.745(3)	104.897(6)
γ/°	88.952(3)	91.039(5)	90	90
Unit cell volume/$Å^3$	5040.6(3)	4686.7(14)	8081.8(4)	13502.2(14)
No. of formula units per unit cell, *Z*	2	2	4	4
Density (calc)/ Mg/m^3	1.263	1.275	1.226	1.224
Absorption coefficient, μ/mm^{-1}	0.665	0.681	0.686	0.704
F(000)	2020	1894	3112	5296
Theta range/°	1.871 to 68.383	3.038 to 68.126	2.803 to 68.314	2.659 to 69.907
Reflections collected	52547	50351	84228	69467
Independent reflections	17955	16648	14677	12172
R_{int}	0.0555	0.1266	0.0576	0.2927
Completeness (to theta)/%	97.9 (67.687°)	97.7 (67.687°)	99.7 (67.684°)	98.8 (67.687°)
Data / restraints / parameter	17955 / 273 / 1422	16648 / 55 / 1272	14677 / 110 / 1071	12172 / 4484 / 1353
Goodness of fit on F^2	1.113	0.939	1.027	1.260
Final R_1 values ($I > 2\sigma(I)$)	0.1014	0.0643	0.1192	0.1365
Final $wR(F^2)$ values ($I > 2\sigma(I)$)	0.3280	0.1586	0.3310	0.3731
Final R_1 values (all data)	0.1132	0.1144	0.1385	0.2673
Final $wR(F^2)$ values (all data)	0.3364	0.1857	0.3459	0.4485
Largest diff. peak and hole/e·$Å^{-3}$	1.055 and -0.528	0.335 and -0.322	0.737 and -0.380	0.993 and -0.835
Absolute structure parameter	-	-	-	-
CCDC number	1914900	1914901	1914902	1914903

Compound 1d

The molecular structure of [(^{Xyl}nacnac)Mg}$_2$] **1d** (see Figure S57) was previously mentioned and the compound has been published though showed relatively poor structural data.[4] The complex crystallized with half a molecule in the asymmetric unit and shows unit cell parameters and an overall packing highly similar to those of [(^{Mes}nacnac)Mg}$_2$] **1c**.[3]

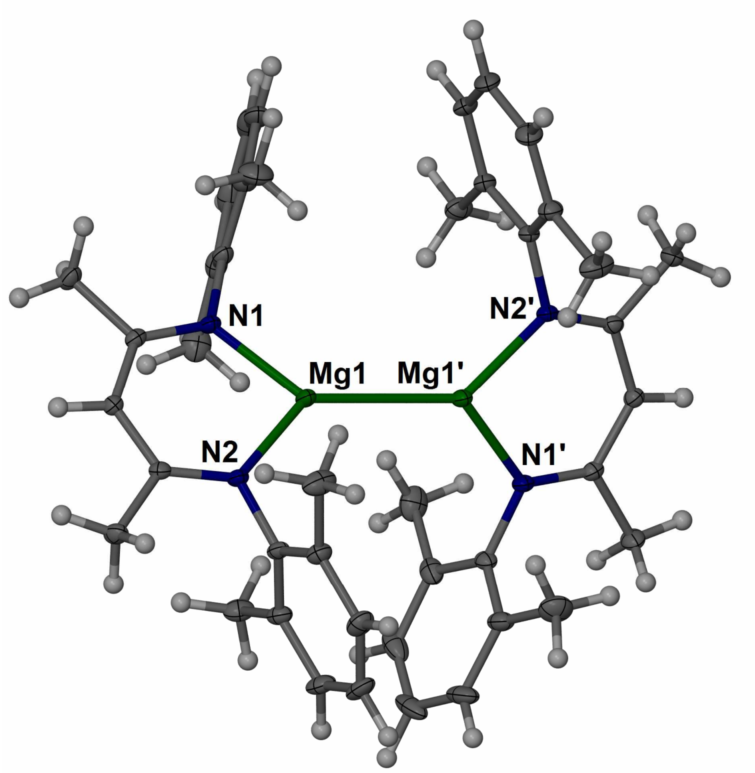

Figure S57. Molecular structure of **1d** (25% thermal ellipsoids). Selected bond lengths and angles: Mg(1)-Mg(1)' 2.8120(8), Mg(1)-N(2) 2.0429(11), Mg(1)-N(1) 2.0430(11), N(1)-C(2) 1.3322(17), N(1)-C(6) 1.4330(16), N(2)-C(4) 1.3236(16), N(2)-C(14) 1.4379(15), C(1)-C(2) 1.5103(17), C(2)-C(3) 1.3978(18), C(3)-C(4) 1.4058(17), C(4)-C(5) 1.5122(18); N(2)-Mg(1)-N(1) 92.39(4), N(2)-Mg(1)-Mg(1)' 135.30(4), N(1)-Mg(1)-Mg(1)' 131.42(4). Sum of angles around Mg1: 359.11(12).

Compound 2b'

The complex [{(^{Dep}nacnac)Mg}$_6$C$_{60}$]·4 C_6H_{14}, **2b'**, see Figure S58, crystallised with a full molecule in the asymmetric unit. Some outer ligand groups are poorly ordered and were modelled with two positions for each atom, their occupancies were refined, and geometry restraints were applied. One ethyl group (C74-C75) was modelled and refined with 67.5 and 32.5% occupancy, most of the atoms of another 2,6-diethylphenyl group (C106-C114) were modelled and refined with 55.5 and 44.5% occupancy, and one outer methyl group (C165) from a third 2,6-diethylphenyl group was modelled and refined with 65.3 and 34.7% occupancy. Minor contributions of the disordered parts are not shown in the image. Severely disordered solvent of crystallisation, *n*-hexane, was removed using the SQUEEZE routine (Solvent Accessible Volume = 1316, # Electrons Found in S.A.V. = 224).

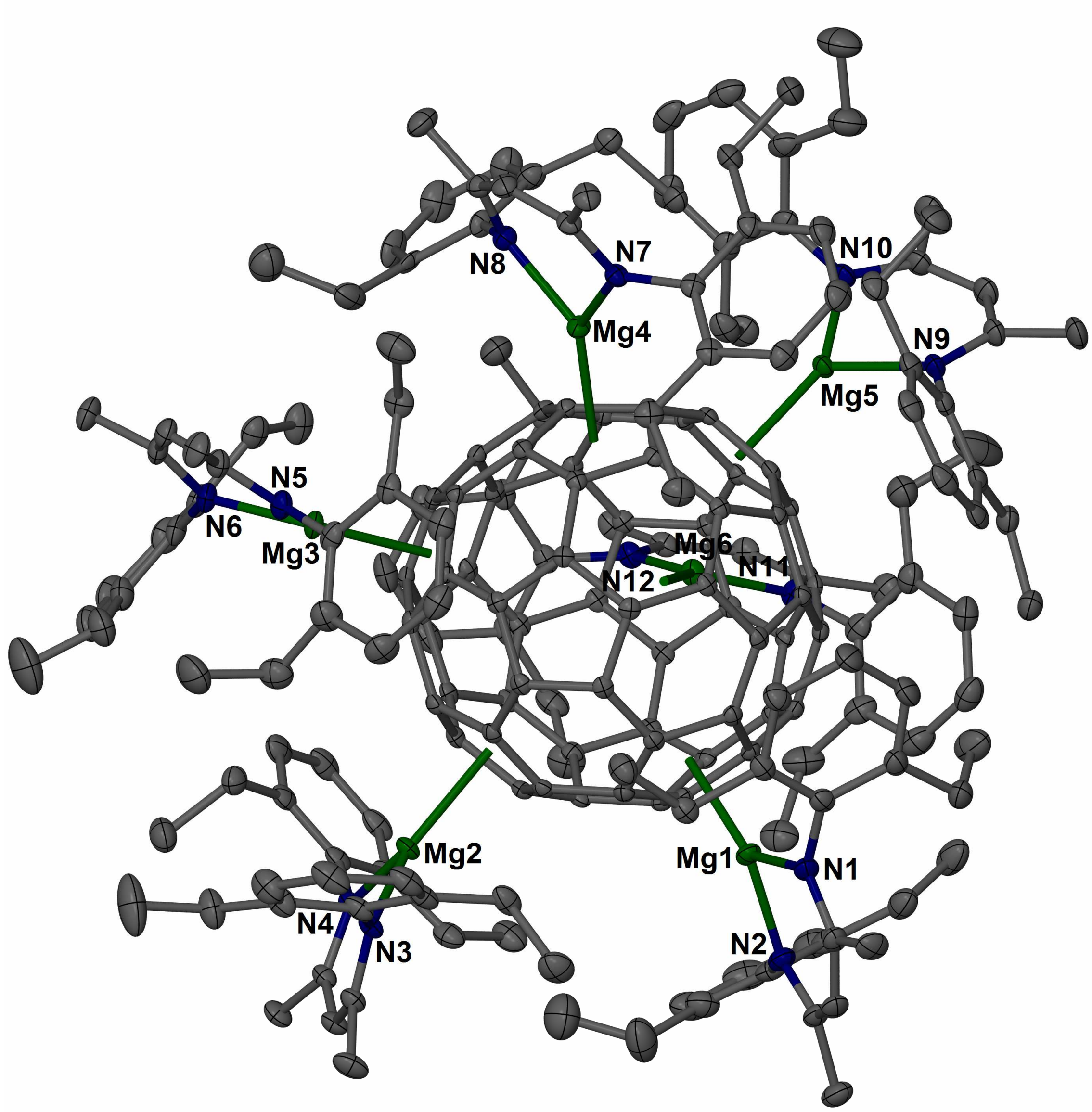

Figure S58. Molecular structure of **2b'** (25% thermal ellipsoids); minor disordered parts and hydrogen atoms not shown. Selected bond lengths and angles: Mg(1)-N(2) 2.012(4), Mg(1)-N(1) 2.017(4), Mg(1)-C(184) 2.385(4), Mg(1)-C(183) 2.402(4), Mg(1)-C(185) 2.505(4), Mg(1)-C(182) 2.511(4), Mg(1)-C(181) 2.551(4), Mg(2)-N(3) 2.015(3), Mg(2)-N(4) 2.035(3), Mg(2)-C(220) 2.443(4), Mg(2)-C(200) 2.444(4), Mg(2)-C(201) 2.499(4), Mg(2)-C(186) 2.527(4), Mg(2)-C(187) 2.603(4), Mg(3)-N(6) 2.023(4), Mg(3)-N(5) 2.034(4), Mg(3)-C(203) 2.391(4), Mg(3)-C(202) 2.417(4), Mg(3)-C(224) 2.450(4), Mg(3)-C(222) 2.584(4), Mg(3)-C(223) 2.596(4), Mg(4)-N(7) 2.012(3), Mg(4)-N(8) 2.031(4), Mg(4)-C(226) 2.392(4), Mg(4)-C(227) 2.396(4), Mg(4)-C(225) 2.474(4), Mg(4)-C(207) 2.578(4), Mg(4)-C(206) 2.616(4), Mg(5)-N(10) 2.019(4), Mg(5)-N(9) 2.032(4), Mg(5)-C(230) 2.472(4), Mg(5)-C(211) 2.473(4), Mg(5)-C(210) 2.504(4), Mg(5)-C(228) 2.505(4), Mg(5)-C(229) 2.537(4), Mg(6)-N(11) 2.003(4), Mg(6)-N(12) 2.003(4), Mg(6)-C(215) 2.402(4), Mg(6)-C(233) 2.417(4), Mg(6)-C(214) 2.470(4), Mg(6)-C(232) 2.474(4), Mg(6)-C(231) 2.477(4); N(2)-Mg(1)-N(1) 94.32(16), N(3)-Mg(2)-N(4) 93.62(14), N(6)-Mg(3)-N(5) 94.41(15), N(7)-Mg(4)-N(8) 95.09(15), N(10)-Mg(5)-N(9) 93.61(15), N(11)-Mg(6)-N(12) 95.29(15).

Compound 2c'

The complex $[\{(^{Mes}nacnac)Mg\}_6C_{60}]\cdot7C_6H_6$, **2c'**, see Figures S59 and S60, crystallised with a full molecule in the asymmetric unit.

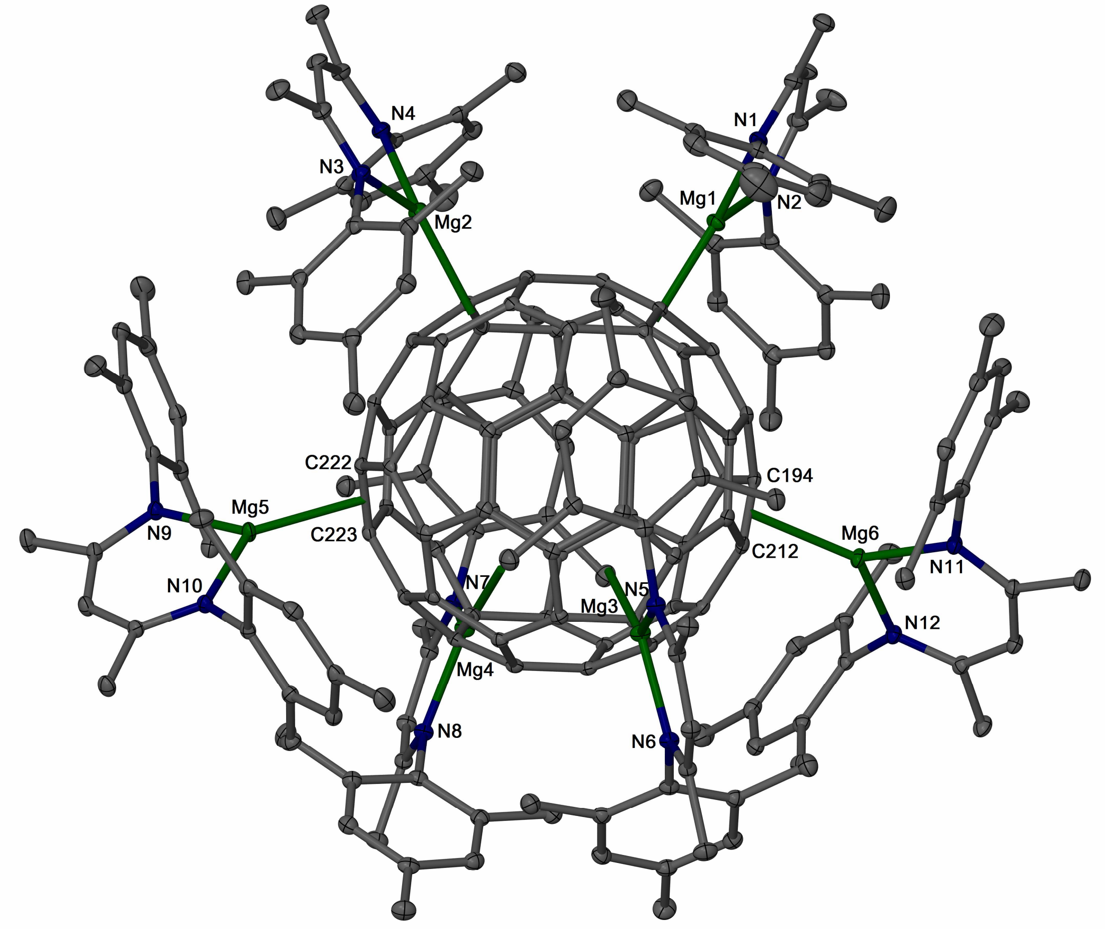

Figure S59. Molecular structure of **2c'** (25% thermal ellipsoids). Solvent molecules and hydrogen atoms not shown. Selected bond lengths and angles: Mg(1)-N(2) 2.0071(14), Mg(1)-N(1) 2.0092(15), Mg(1)-C(181) 2.3991(15), Mg(1)-C(185) 2.4258(15), Mg(1)-C(184) 2.4311(16), Mg(1)-C(182) 2.4671(16), Mg(1)-C(183) 2.4752(16), Mg(2)-N(4) 2.0058(14), Mg(2)-N(3) 2.0077(14), Mg(2)-C(186) 2.4071(15), Mg(2)-C(187) 2.4452(15), Mg(2)-C(201) 2.4453(16), Mg(2)-C(200) 2.4650(16), Mg(2)-C(220) 2.4766(16), Mg(3)-N(5) 2.0077(14), Mg(3)-N(6) 2.0114(14), Mg(3)-C(233) 2.4390(16), Mg(3)-C(214) 2.4417(16), Mg(3)-C(232) 2.4469(15), Mg(3)-C(215) 2.4534(16), Mg(3)-C(231) 2.4569(15), Mg(4)-N(7) 2.0122(14), Mg(4)-N(8) 2.0128(14), Mg(4)-C(225) 2.4309(15), Mg(4)-C(207) 2.4374(16), Mg(4)-C(206) 2.4442(16), Mg(4)-C(227) 2.4468(16), Mg(4)-C(226) 2.4545(15), Mg(5)-N(9) 2.0015(14), Mg(5)-N(10) 2.0023(14), Mg(5)-C(223) 2.2703(15), Mg(5)-C(222) 2.3528(15), Mg(6)-N(11) 1.9979(14), Mg(6)-N(12) 2.0008 (14), Mg(6)-C(212) 2.2647(15), Mg(6)-C(194) 2.3413(15); N(2)-Mg(1)-N(1)

94.35(6), N(4)-Mg(2)-N(3) 94.01(6), N(5)-Mg(3)-N(6) 95.31(6), N(7)-Mg(4)-N(8) 95.99(6), N(9)-Mg(5)-N(10) 97.03(6), N(11)-Mg(6)-N(12) 96.77(6).

Fulleride C-C distances in five-membered rings (in Å): With Mg coordination: 1.456(2), 1.455(2), 1.455(2), 1.453(2), 1.453(2), 1.453(2), 1.450(2), 1.450(2), 1.450(2), 1.449(2), 1.449(2), 1.448(2), 1.447(2), 1.446(2), 1.446(2), 1.445(2), 1.443(2), 1.442(2), 1.440(2), 1.438(2) [mean: 1.4484]. Without Mg coordination: 1.446(2), 1.445(2), 1.444(2), 1.444(2), 1.443(2), 1.443(2), 1.440(2), 1.439(2), 1.439(2), 1.437(2), 1.437(2), 1.434(2), 1.434(2), 1.434(2), 1.429(2) [mean 1.4389].

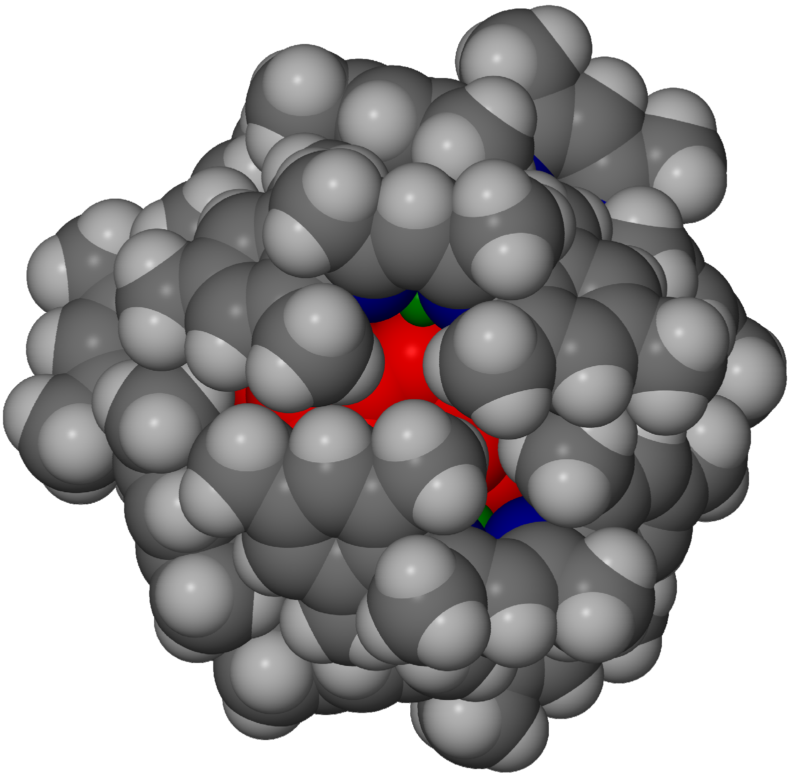

Figure S60. Space-filling model for **2c'** (C_{60} red, Mg green, N blue, C grey).

Compound 2c''

The complex [{(^{Mes}nacnac)Mg}$_6$C$_{60}$]·4C$_6$H$_{14}$, **2c''**, see Figure S61, crystallised with two full molecules in the asymmetric unit. Both independent molecules show the same overall geometry and Mg···C$_{60}$ coordination and thus only one is shown below and discussed in the main text. Severely disordered solvent of crystallisation, *n*-hexane, was removed using the SQUEEZE routine (Solvent Accessible Volume = 4071, Electrons Found in S.A.V. = 801).

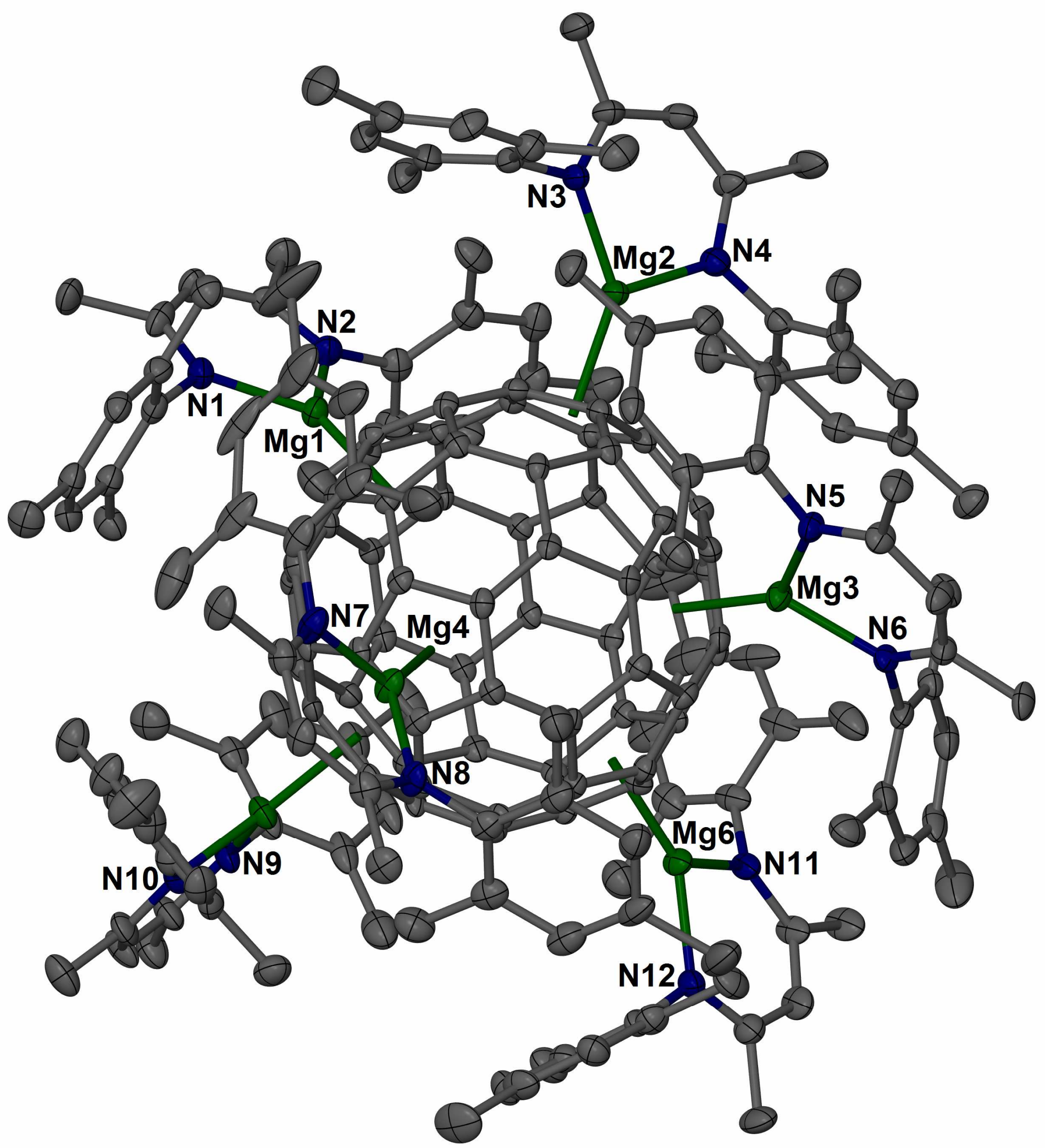

Figure S61. Molecular structure of, **2c''** (25% thermal ellipsoids). Only one independent molecule is shown. Solvent molecules and hydrogen atoms not shown. Selected bond lengths and angles are given for the shown independent molecule only: Mg(1)-N(1) 1.996(8), Mg(1)-N(2) 1.999(9), Mg(1)-C(181) 2.444(9), Mg(1)-C(182) 2.471(9), Mg(1)-C(183) 2.473(10), Mg(1)-C(184) 2.485(9),

Mg(1)-C(185) 2.495(9), Mg(2)-N(4) 1.991(9), Mg(2)-N(3) 1.993(8), Mg(2)-C(186) 2.414(9), Mg(2)-C(200) 2.422(9), Mg(2)-C(220) 2.462(9), Mg(2)-C(187) 2.518(9), Mg(2)-C(201) 2.521(10), Mg(3)-N(6) 1.997(8), Mg(3)-N(5) 2.010(8), Mg(3)-C(223) 2.425(8), Mg(3)-C(222) 2.425(9), Mg(3)-C(221) 2.533(10), Mg(3)-C(237) 2.553(10), Mg(3)-C(235) 2.651(9), Mg(3)-C(236) 2.666(10), Mg(4)-N(7) 1.982(8), Mg(4)-N(8) 2.014(9), Mg(4)-C(226) 2.377(8), Mg(4)-C(227) 2.397(8), Mg(4)-C(225) 2.476(9), Mg(4)-C(207) 2.522(9), Mg(4)-C(206) 2.555(9), Mg(5)-N(9) 1.997(9), Mg(5)-N(10) 2.013(9), Mg(5)-C(194) 2.403(9), Mg(5)-C(212) 2.454(9), Mg(5)-C(193) 2.494(10), Mg(5)-C(211) 2.612(9), Mg(5)-C(209) 2.633(9), Mg(5)-C(210) 2.721(10), Mg(6)-N(11) 1.981(8), Mg(6)-N(12) 2.007(9), Mg(6)-C(232) 2.406(8), Mg(6)-C(231) 2.409(8), Mg(6)-C(214) 2.475(9), Mg(6)-C(233) 2.507(9), Mg(6)-C(215) 2.548(10); N(1)-Mg(1)-N(2) 96.9(4), N(4)-Mg(2)-N(3) 96.1(3), N(6)-Mg(3)-N(5) 94.4(3), N(7)-Mg(4)-N(8) 95.1(3), N(9)-Mg(5)-N(10) 94.5(4), N(11)-Mg(6)-N(12) 94.1(3).

Compound 2d'

The complex $[\{(^{Xyl}nacnac)Mg\}_6C_{60}]\cdot 7C_6H_6$, **2d'**, see Figures S62 and S63, crystallized with half a molecule in the asymmetric unit.

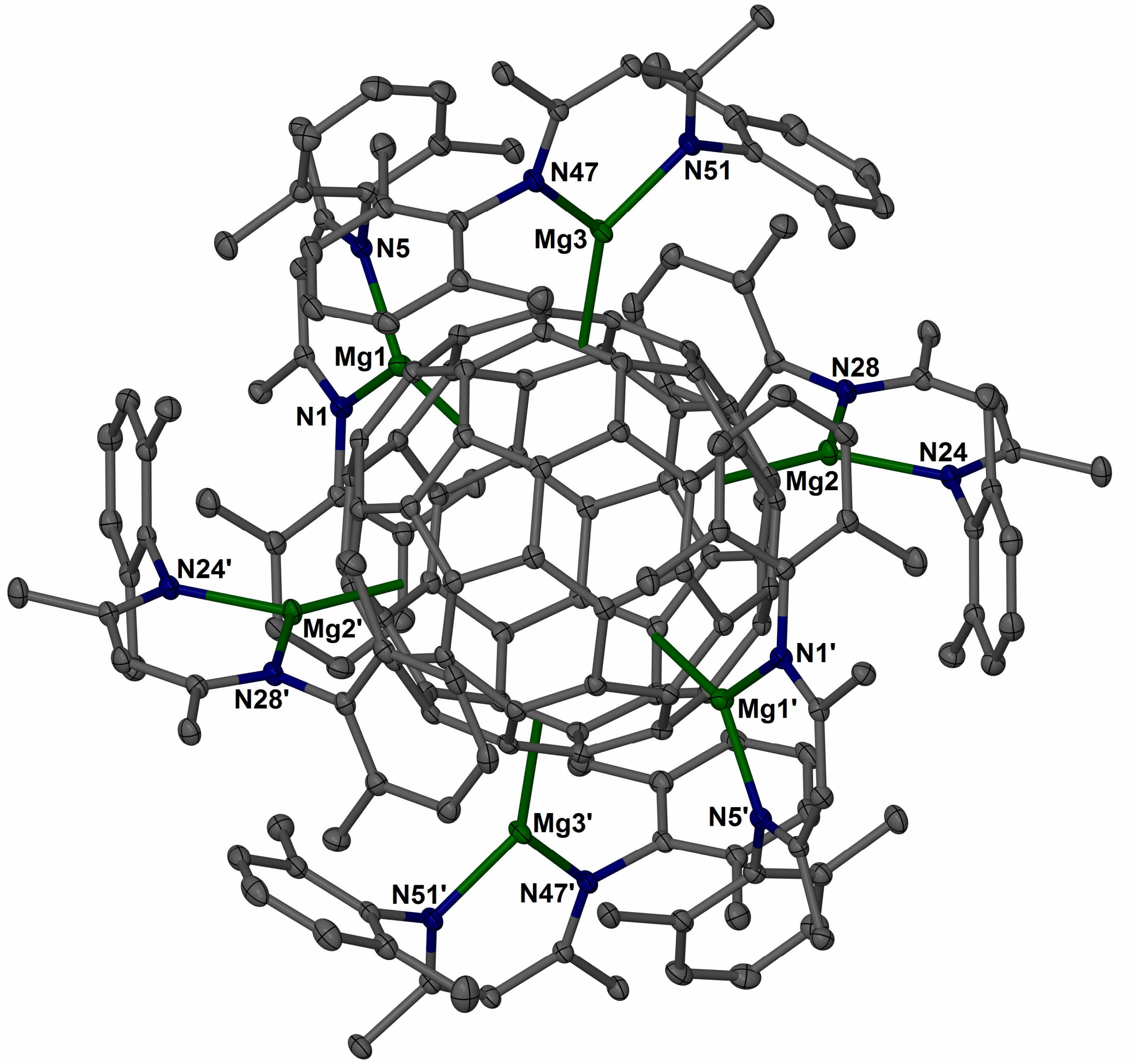

Figure S62. Molecular structure of **2d'** (25% thermal ellipsoids). Solvent molecules and hydrogen atoms not shown. Selected bond lengths and angles: Mg(1)-N(1) 2.0095(12), Mg(1)-C(81) 2.3656(14), Mg(1)-C(76) 2.4059(14), Mg(1)-C(99)' 2.4241(14), Mg(1)-C(75) 2.4674(14), Mg(1)-C(70) 2.4799(14), Mg(2)-N(28) 2.0079(12), Mg(2)-N(24) 2.0167(13), Mg(2)-C(94) 2.4218(14), Mg(2)-C(83)' 2.4401(14), Mg(2)-C(93) 2.5071(14), Mg(2)-C(82)' 2.5336(14), Mg(2)-C(72)' 2.6082(14), Mg(2)-C(73)' 2.6192(14), Mg(3)-N(47) 1.9805(12), Mg(3)-N(51) 1.9867(12), Mg(3)-C(90) 2.2551(14), Mg(3)-C(97)' 2.2660(14); N(5)-Mg(1)-N(1) 96.28(5), N(28)-Mg(2)-N(24) 95.29(5), N(47)-Mg(3)-N(51) 98.09(5).

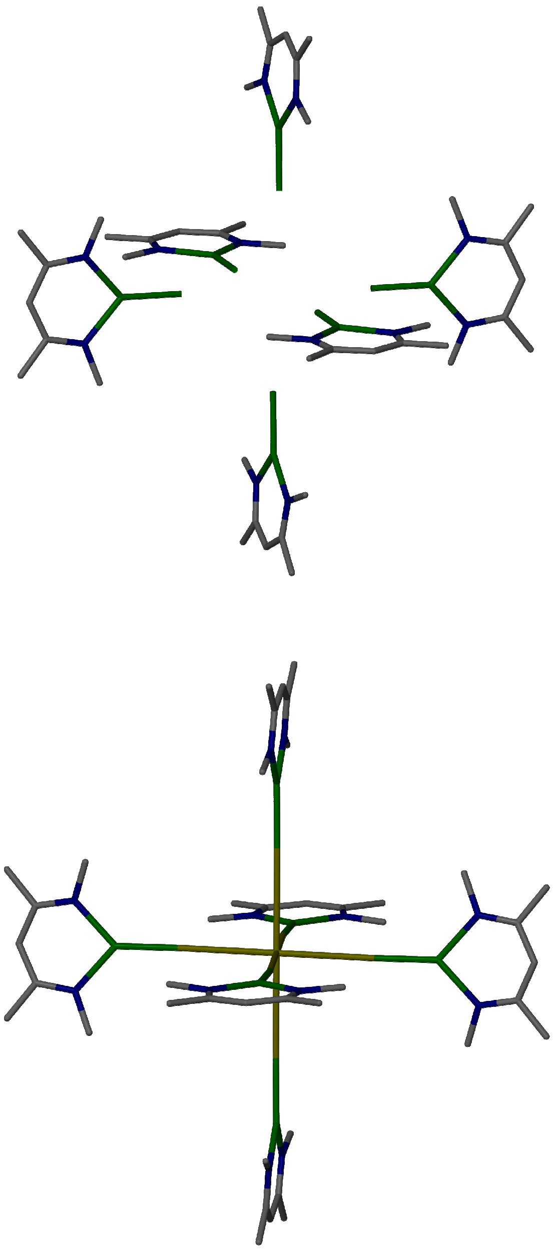

Figure S63. Cut-back wire-frame views (ipso-aryl carbon atoms only, no H atoms) of **2d'** to highlight the octahedral orientation of $[(^{Xyl}nacnac)Mg]^+$ groups. Top: without C_{60} fragment including Mg bonds to fulleride fragments (ring centroids or bond midpoints); bottom: C_{60} fragment replaced by its centre, including Mg bonds to fulleride fragments (green) and further bonds to centre (yellow).

Compound 3a'

The complex [{(^{Dip}nacnac)Mg}$_2$C$_{60}$]·4C$_6$H$_6$, **3a'**, see Figure S64, crystallized with a full molecule in the asymmetric unit. Geometry restraints were applied to refine several benzene solvent molecules. A simplified partial packing of the molecules, showing no close C$_{60}$···C$_{60}$ interactions, is given in Figure S65.

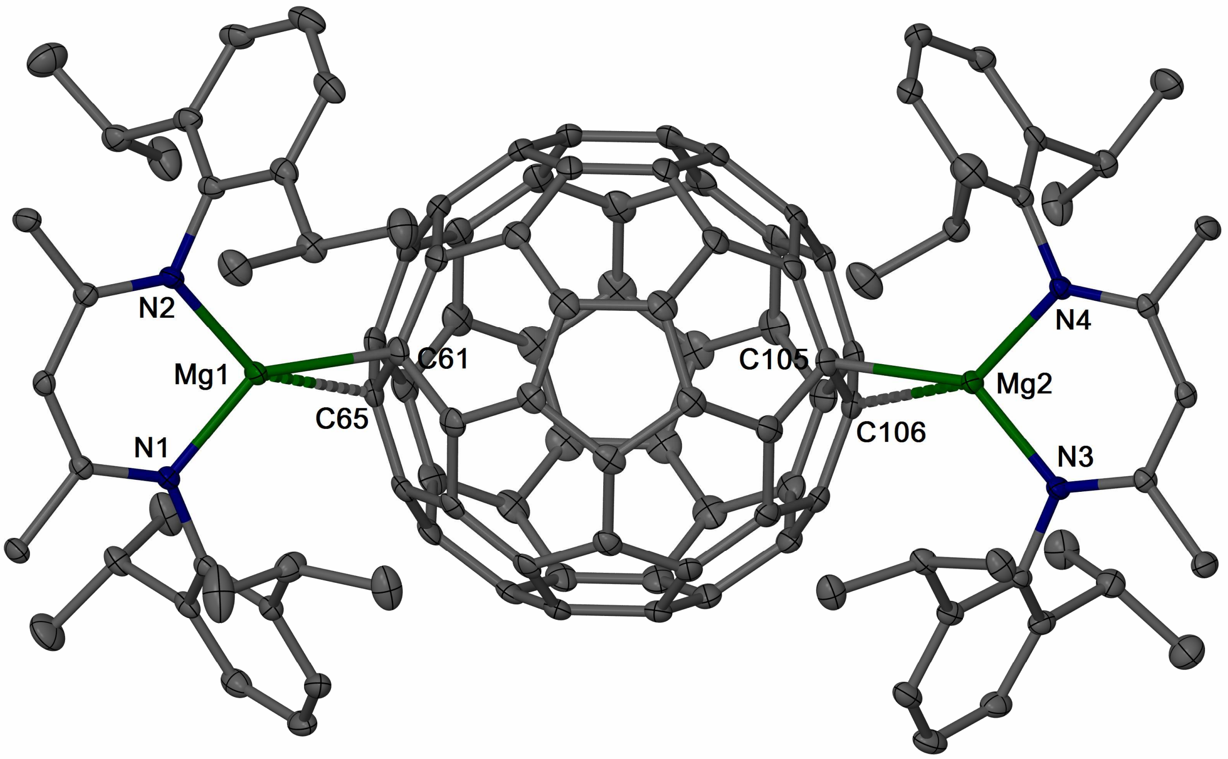

Figure S64. Molecular structure of **3a'** (25% thermal ellipsoids). Solvent molecules and hydrogen atoms not shown. Selected bond lengths and angles: Mg(1)-N(2) 1.967(4), Mg(1)-N(1) 1.980(4), Mg(1)-C(61) 2.209(5), Mg(1)-C(65) 2.668(6), Mg(2)-N(4) 1.969(4), Mg(2)-N(3) 1.979(4), Mg(2)-C(105) 2.224(5), Mg(2)-C(106) 2.658(6); N(2)-Mg(1)-N(1) 96.87(16), N(2)-Mg(1)-C(61) 126.34(18), N(1)-Mg(1)-C(61) 136.67(18), N(4)-Mg(2)-N(3) 95.94(16), N(4)-Mg(2)-C(105) 127.95(18), N(3)-Mg(2)-C(105) 136.08(18). Sum of angles: Mg1: 359.9(5); Mg2: 360.0(5).

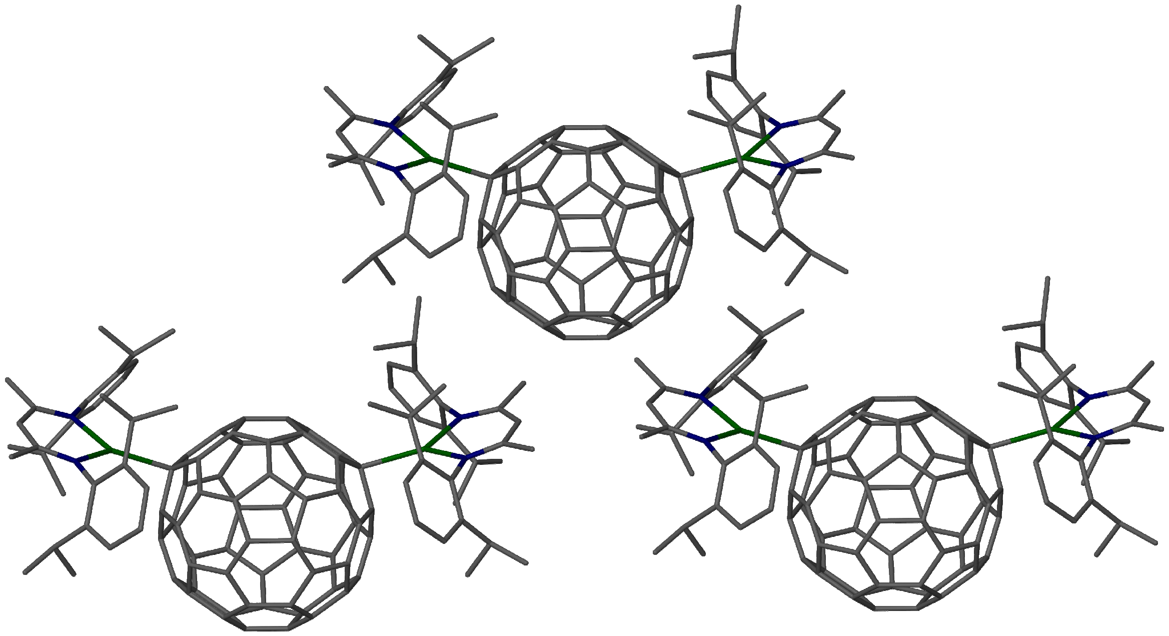

Figure S65. Simplified partial packing of molecules of **3a'**; wire frame representation. Solvent molecules not shown.

Compound 3a''

The complex $[\{(^{Dip}nacnac)Mg\}_2C_{60}]\cdot 2.5C_6H_6$, **3a''**, see Figure S66, crystallized as in the case of **3a'** with a full molecule in the asymmetric unit. Geometry restraints were applied to refine the benzene solvent molecules. The overall structure (including the Mg···C_{60} coordination) and packing is highly comparable to that of **3a'** again showing no close C_{60}···C_{60} interactions.

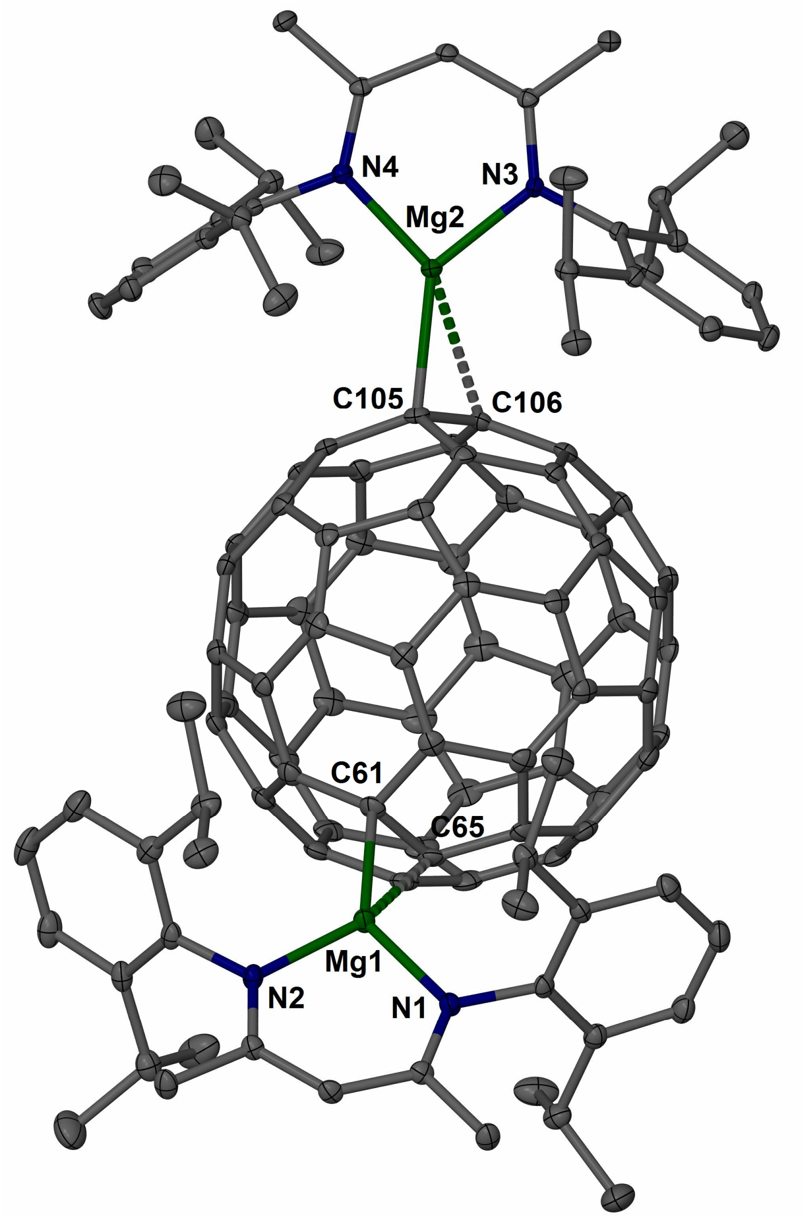

Figure S66. Molecular structure of **3a''** (25% thermal ellipsoids). Solvent molecules and hydrogen atoms not shown. Selected bond lengths and angles: Mg(1)-N(2) 1.966(3), Mg(1)-N(1) 1.981(2), Mg(1)-C(61) 2.199(3), Mg(1)-C(65) 2.683(4), Mg(2)-N(4) 1.972(2), Mg(2)-N(3) 1.983(3), Mg(2)-C(105) 2.214(3), Mg(2)-C(106) 2.709(3); N(2)-Mg(1)-N(1) 98.21(11), N(2)-Mg(1)-C(61)

125.49(12), N(1)-Mg(1)-C(61) 136.01(12), N(4)-Mg(2)-N(3) 95.19(10), N(4)-Mg(2)-C(105) 130.23(12), N(3)-Mg(2)-C(105) 134.39(11). Sum of angles: Mg1: 359.7(4), Mg2: 359.8(3).

Compound 3b'

The complex $[\{(^{Dep}nacnac)Mg\}_2C_{60}]\cdot 1.5C_6H_6$, **3b'**, see Figure S67, crystallized with a full molecule in the asymmetric unit. The molecular structure suffers in general from poor ordering and large thermal ellipsoids. Some outer ligand groups are poorly ordered and were modelled with two positions for each atom, their occupancies were refined, and geometry restraints were applied. A methyl group (C43) from a 2,6-diethylphenyl group was modelled and refined with 58.5 and 41.5% occupancy, and another methyl group (C53) from the same 2,6-diethylphenyl group was modelled and refined with 66.4 and 33.6% occupancy. Restraints to bond distances and thermal motion were applied during the refinement of other poorly ordered ethyl groups. Minor contributions of the disordered parts are not shown in the image. Solvent of crystallisation, benzene, was removed using the SQUEEZE routine (Solvent Accessible Volume = 1119, # Electrons Found in S.A.V. = 260).

The two $(^{Dep}nacnac)Mg$ cations coordinate in a more acute fashion to C_{60}^{2-} when the $Mg\cdots C_{60\text{-centre}}\cdots Mg$ angle (*ca.* 79°) is considered, compared to those of **3a'** and **3a''** (both *ca.* 126°). A partial simplified packing diagram is shown in Figure S68. This molecular geometry allows packing with relatively close interactions between C_{60}^{2-} units that are arranged in a one-dimensional stack and are only slightly offset. The closest approach is via two co-planar five-membered carbon rings on neighbouring C_{60}^{2-} moieties with shortest intermolecular C···C interactions of *ca.* 3.4 Å.

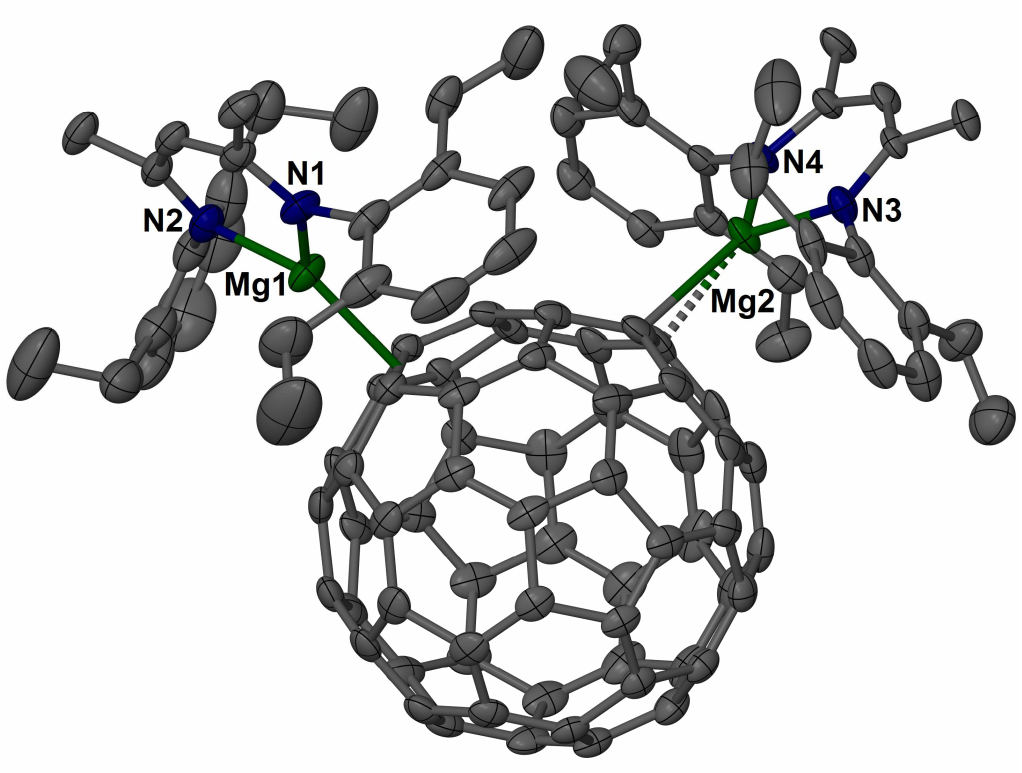

Figure S67. Molecular structure of **3b'** (25% thermal ellipsoids). Minor disordered parts and hydrogen atoms not shown. Selected bond lengths and angles: Mg(1)-N(1) 1.957(6), Mg(1)-N(2) 1.973(6), Mg(1)-C(66) 2.272(6), Mg(1)-C(61) 2.341(7), Mg(2)-N(4) 1.950(5), Mg(2)-N(3) 1.973(5), Mg(2)-C(76) 2.206(6), Mg(2)-C(77) 2.551(7); N(1)-Mg(1)-N(2) 96.3(3), N(4)-Mg(2)-N(3) 98.2(2).

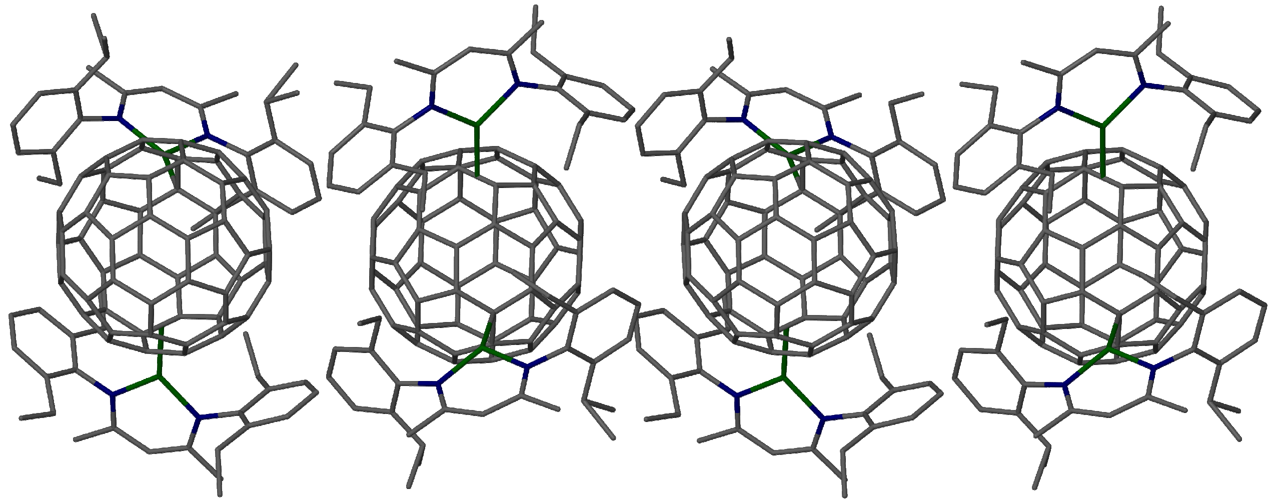

Figure S68. Simplified partial packing of molecules of **3b'**; wire frame representation.

Compound 4a

The complex [{(^{Dip}nacnac)Mg}$_4$C$_{60}$], **4a**, sees Figures S69, crystallized with half a full molecule in the asymmetric unit. The molecular structure suffers in general from poor ordering, including the C$_{60}$ unit, relatively large thermal ellipsoids and high values for R_1 (13.7%) and R_{int} (29.3%), likely arising from poor crystal-quality and weak diffraction; however, the molecular connectivity is unambiguous. The position and orientation of the four (^{Dip}nacnac)Mg with respect to the fulleride centre are shown in Figure S70.

In **4a**, the disordered C$_{60}$ section was modelled by constructing two independent full C$_{60}$ models (with half-occupancy atoms and with bonds to symmetry-related atoms suppressed), constrained to idealised geometries, and their contributions were freely refined anisotropically to 70.5 and 29.5%, respectively, resulting in four positions for each carbon atom in C$_{60}$. The final model was refined with restraints to thermal motion for the C$_{60}$ section. The 2,6-diisopropylphenyl substituent on N4 shows generally poor ordering and an isopropyl group (C57-C59) was modelled with two positions for each atom (refined occupancies 62.0 and 38.0%) and refined anisotropically with geometry and thermal restraints.

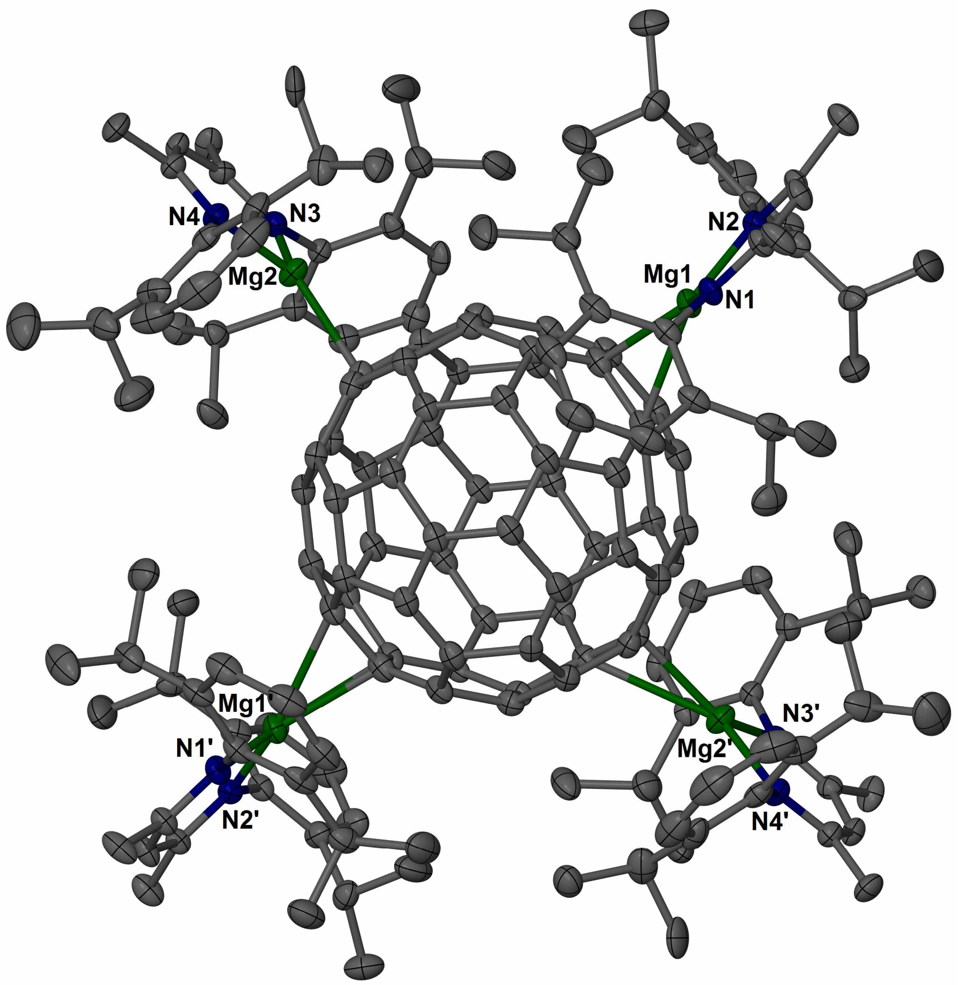

Figure S69. Molecular structure of **4a** (25% thermal ellipsoids). Only one of four sets of C_{60} positions shown. Minor disordered parts and hydrogen atoms not shown. Selected bond lengths and angles: Mg(1)-N(1) 1.980(7), Mg(1)-N(2) 1.988(7), Mg(2)-N(3), 1.977(7), Mg(2)-N(4) 1.988(7); N(1)-Mg(1)-N(2) 96.4(3), N(3)-Mg(2)-N(4) 94.6(3); Mg-C distances are not given due to poor overall ordering.

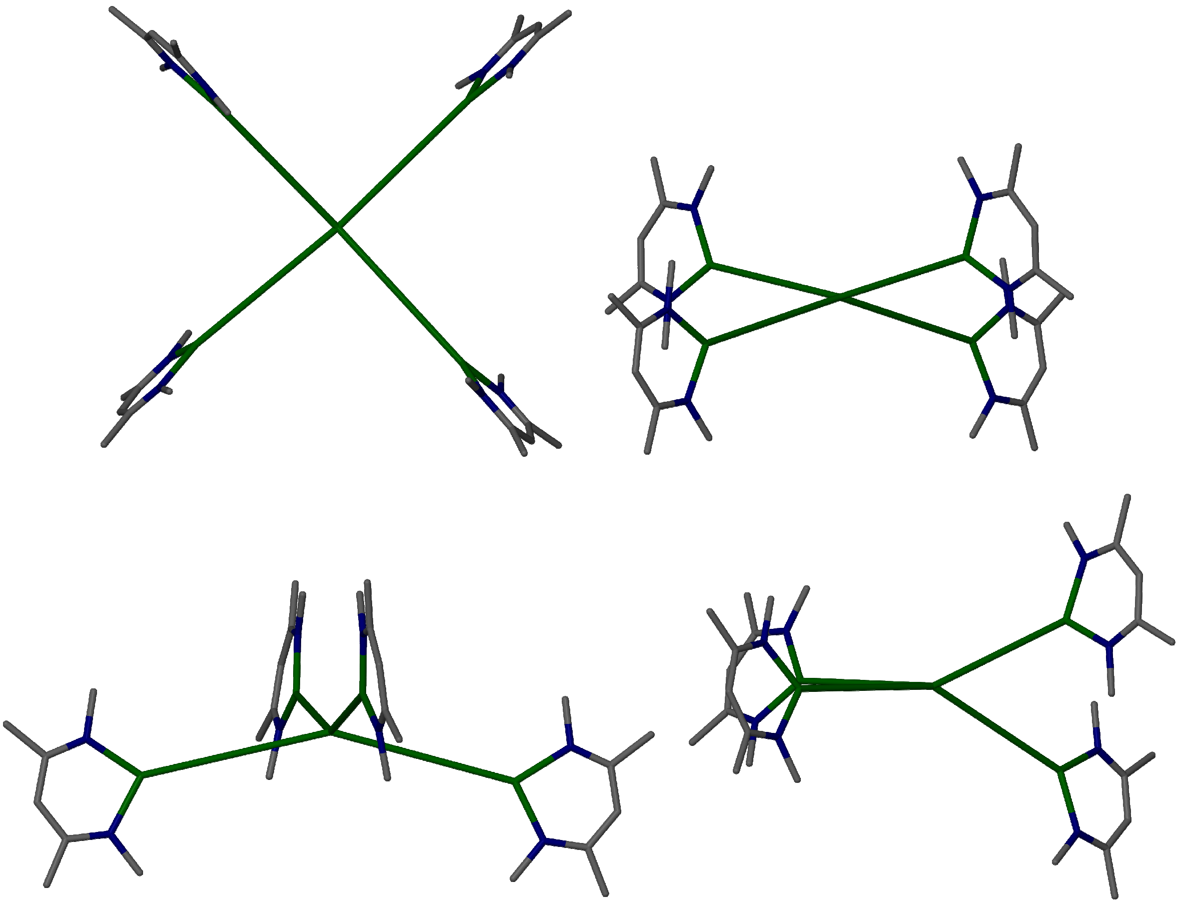

Figure S70. Cut-back wire-frame views of **4a** with the C_{60} fragment replaced a C_{60}-centre position to visualise the geometry.

4 Computational studies

Computations using density functional theory were carried out at the pbe0[17]/def2-svp[18] level of theory with PCM (heptane), ultrafine grid, using G16 Rev A.03.[19] Basis sets were obtained from the Basis Set Exchange.[20] Computations were carried out on the cut-back model complex $[\{(^{Me}nacnac)Mg\}_6C_{60}]$ **2Me**, where ^{Me}nacnac = $HC(MeCNMe)_2$, and on C_{60}, C_{60}^{6-} and $[(^{Me}nacnac)Mg]^+$, respectively, for comparison at the same level of theory. All molecules or ions were optimized in their singlet states. Calculations at the tzvp level of theory afforded highly similar results with respect to orbital energies and orbital shapes; only the svp results are shown here. Four different starting geometries of **2Me** were derived from molecular structures obtained by single crystal X-ray diffraction and their geometries optimised. This led to four different isomers; **2Me-1** (using **2c'** as a starting geometry), **2Me-2** (from **2b'**), **2Me-3** (from **2c''**) and **2Me-4** (from **2d'**). That a local minimum had been obtained was tested using normal mode analysis. A correction for dispersion (pbe0-GD3-BJ)[21] for isomers of **2Me** was applied to test the impact on the energies of the isomers (Table S2) and it is worth noting that the effect on molecules with a full ligand sphere is expected to be larger. Natural bond order analyses were carried out using NBO6.[22]

Geometry optimisation on the **2Me** isomers led to some movement of the $[(^{Me}nacnac)Mg]^+$ positions. The relative energies of the resulting isomers and the Mg coordination modes are summarised in Table S2. Images of the optimised geometries of **2Me-1** to **2Me-4** are presented in Figures S71-S74.

Table S2. Relative energies, Mg coordination modes of 2Me isomers and C_{60} charges.

	**2Me-1**	**2Me-2**	**2Me-3**	**2Me-4**
rel. isomer energy svp [kJ/mol (kcal/mol)]	+12.4 (2.96)	+3.86 (0.924)	0 (0)	+19.9 (4.76)
rel. isomer energy, plus dispersion [kJ/mol (kcal/mol)]	+24.9 (+5.96)	+13.8 (+3.30)	0 (0)	+23.9 (+5.71)
Mg η^6	-	-	-	-
Mg η^5	4	6	5	2
Mg η^2 ([6,6])	-	-	1	3
Mg η^2 ([5,6])	2	-	-	-
Mg η^1	-	-	-	1
Sum of C_{60} charges (NBO)			-5.8	

It is highly likely that further isomers would optimise to stable geometries and could show lower energies than isomer **2Me-3**. Isomers **2Me-1**, **2Me-2** and **2Me-3** all show experimentally observed Mg···C_{60} coordination modes and lie within 3 kcal/mol, or 6 kcal/mol when a correction for dispersion effects is added. Because the full ligand sphere and crystal packing effects could not be included in the calculations, and we observed some movement of $[(^{Me}nacnac)Mg]^+$ positions during the optimisations, we consider the isomers relatively close in energy for the purpose of this study. Isomer **2Me-4** lies almost 5 kcal/mol (no dispersion considered) above **2Me-3** and shows an experimentally not observed terminal (η^1) Mg···C_{60} coordination mode. The molecular orbital shapes of the four isomers are similar and those of isomer **2Me-1** have been used for the discussion and the orbital images. Overall, Mg η^5 and η^2 coordination modes seem to be favoured in the isomers and likely contribute to a low complex energy. An Mg η^6 coordination mode was not found from DFT optimisations though was present in some crystal structures.

Inspecting the NBO charges shows the expected values for C_{60} and C_{60}^{6-}. For neutral C_{60}, an average charge of 0.00 is found, and for C_{60}^{6-} an average charge on a carbon atom of -0.1 is found (range from -0.105 to -0.095). The four isomers of **2Me** show a larger variation of charges on their central C_{60} fragments and depend on nearby Mg coordination which appears to lead to significant polarisation. Generally, carbon atoms that coordinate to Mg show a more negative charge than their neighbours. This effect is most pronounced in carbon centres with low hapticity Mg coordination. The carbon atom binding in a η^1 fashion to an Mg centre (terminal) in **2Me-4** carries a charge of -0.41, whereas its three direct neighbours show an average charge of -0.06, respectively. In isomer **2Me-3**, the average value for the charges of an η^2 coordinated carbon atom is *ca.* -0.28. Its four

neighbours again carry a significantly lower charge of *ca.* -0.02. Less polarisation of the central C_{60} unit is generally found around η^5 coordination modes. Calculated charges on the magnesium centres in isomers **2Me** are typically +1.76-1.78 (*c.f.* +1.77 calculated for free [(^{Me}nacnac)Mg^+]) and +1.73 for the Mg centre with η^1 coordination in **2Me-4**. The ^{Me}nacnac-fragments carry as expected some anionic charge. The sum of charges on the C_{60} fragment in **2Me-3** (the lowest energy isomer) is -5.8. These values support that the complexes can be regarded as contact ion systems with a central C_{60}^{6-} ion coordinating to six [(^{Me}nacnac)Mg]$^+$ cations and that indeed ionic interactions are in operation. Similarly, the electrostatic potential (a colour-coded image of the calculated work done in bringing a positive point charge from infinity to a surface point in the molecule) supports this, please see the main manuscript.

Partial molecular orbital diagrams with orbital images for the species **2Me-1** (Figure S75, also see the main manuscript), C_{60} (Figure S76), C_{60}^{6-} (Figure S77), and [(^{Me}nacnac)Mg]$^+$ (Figure S78) were calculated at the pbe0/def2-svp level of theory.

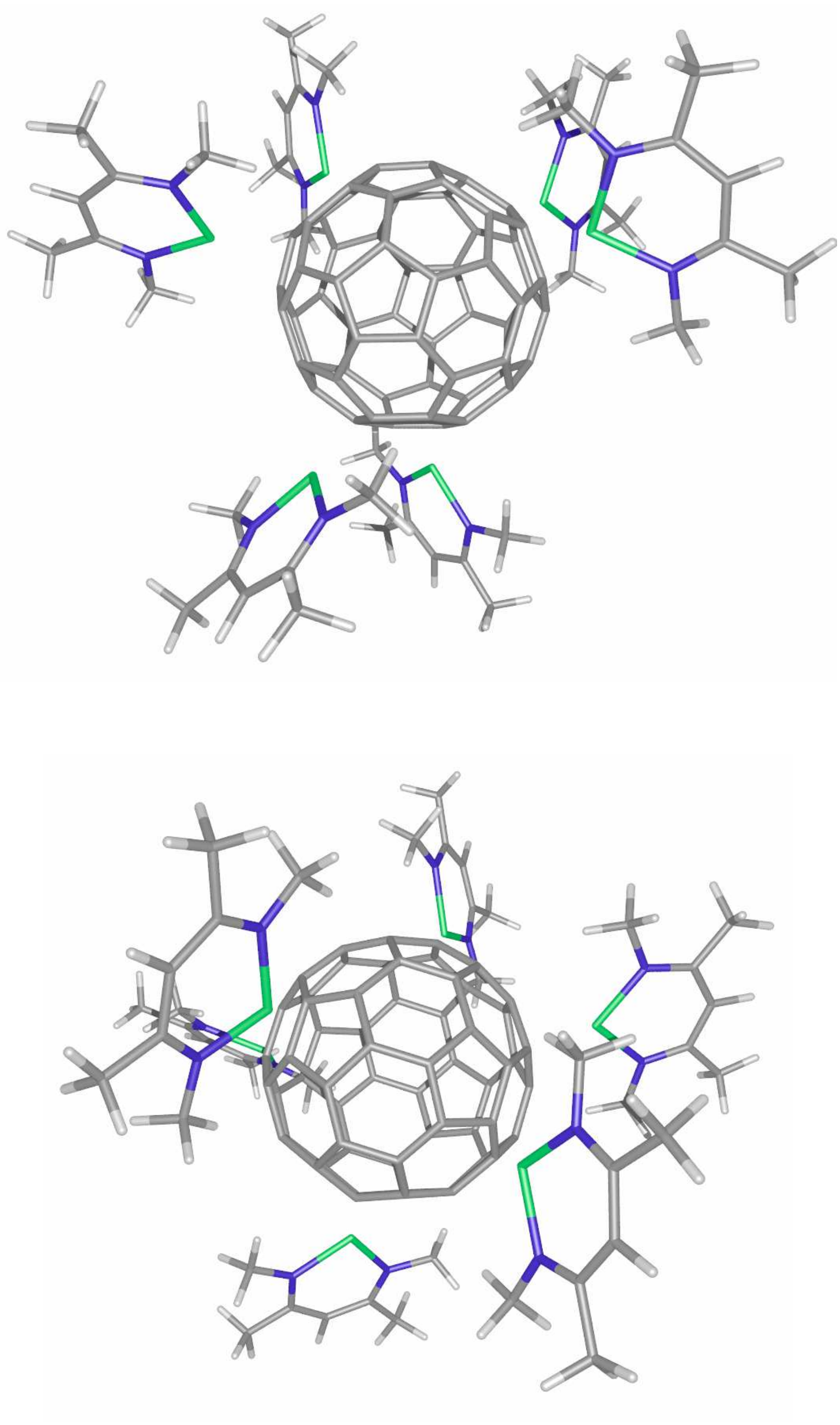

Figure S71. Optimised geometry of isomer **2Me-1**, two views.

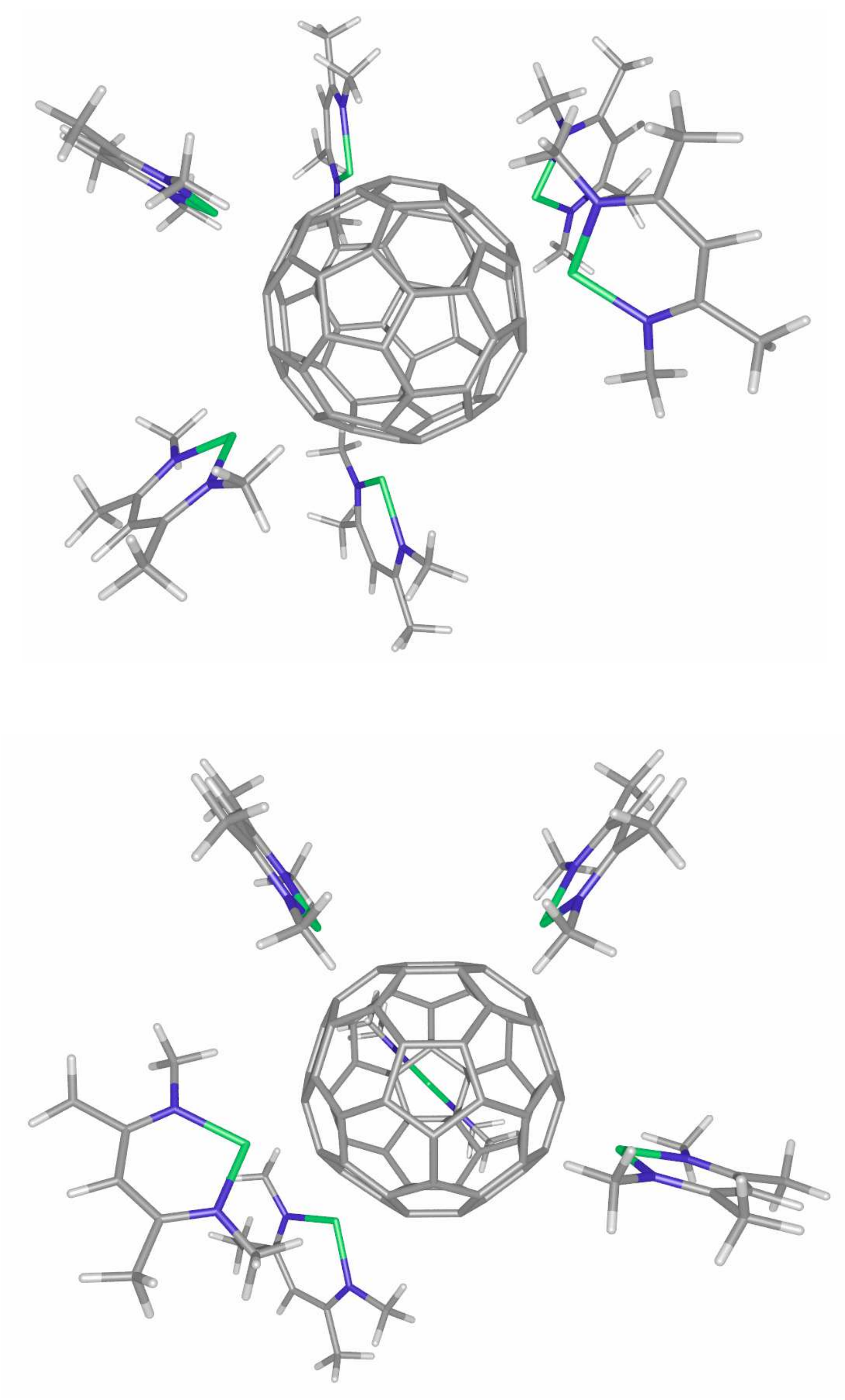

Figure S72. Optimised geometry of isomer **2Me-2**, two views.

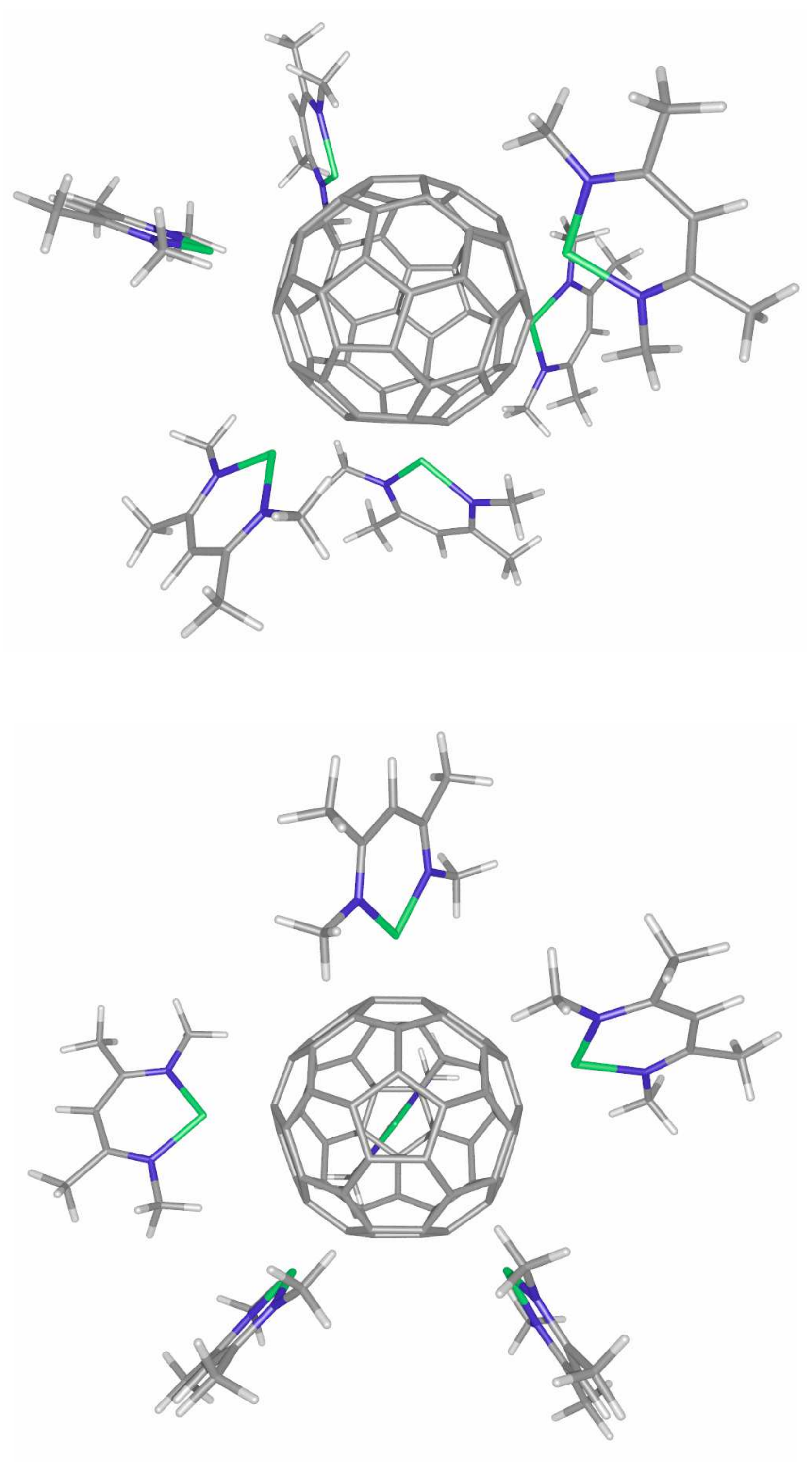

Figure S73. Optimised geometry of isomer **2Me-3**, two views.

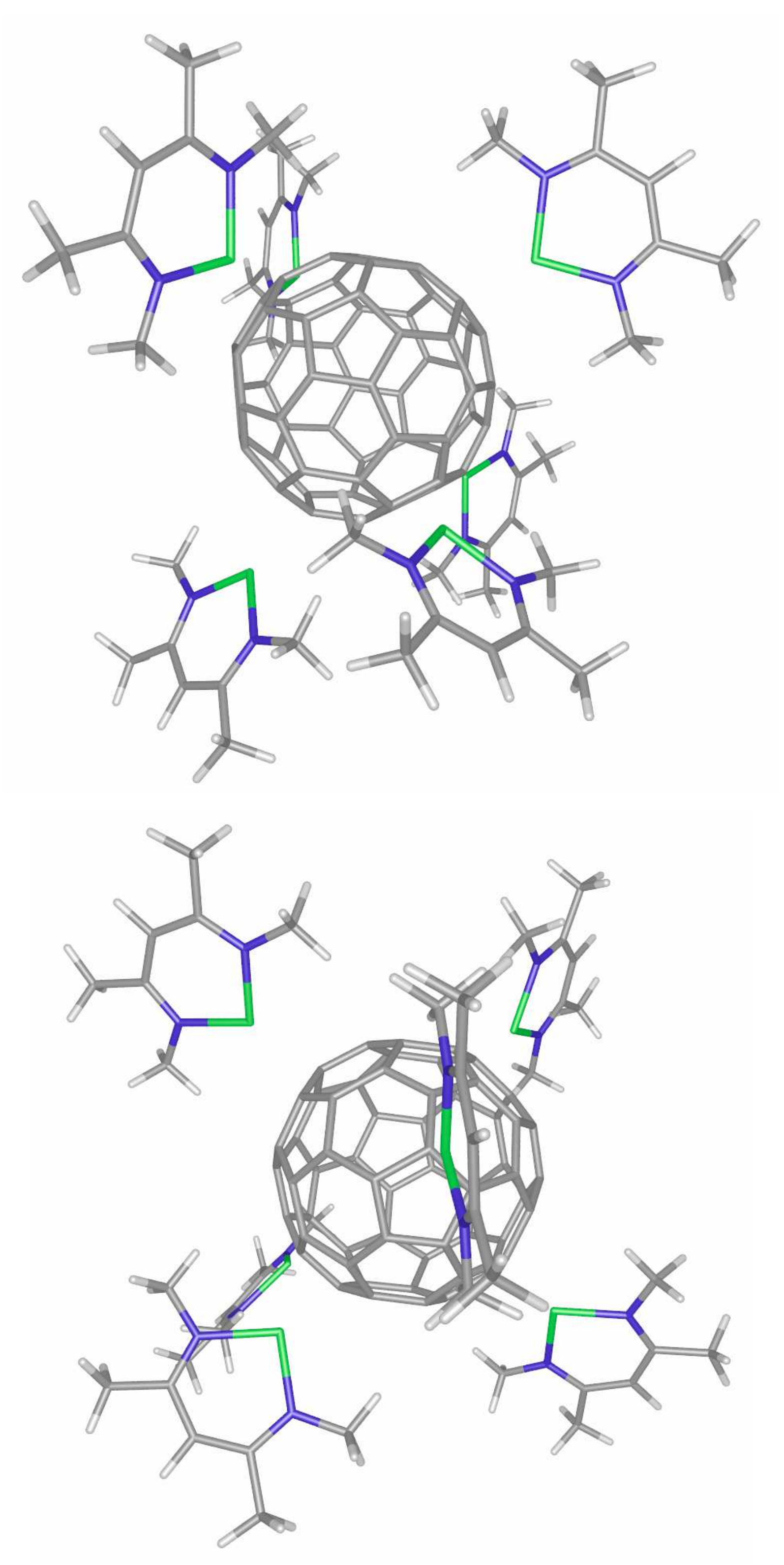

Figure S74. Optimised geometry of isomer **2Me-4**, two views. The η^1 Mg···C_{60} coordination mode is found for the top right (^{Me}nacnac)Mg unit (top image), and in the front (bottom image).

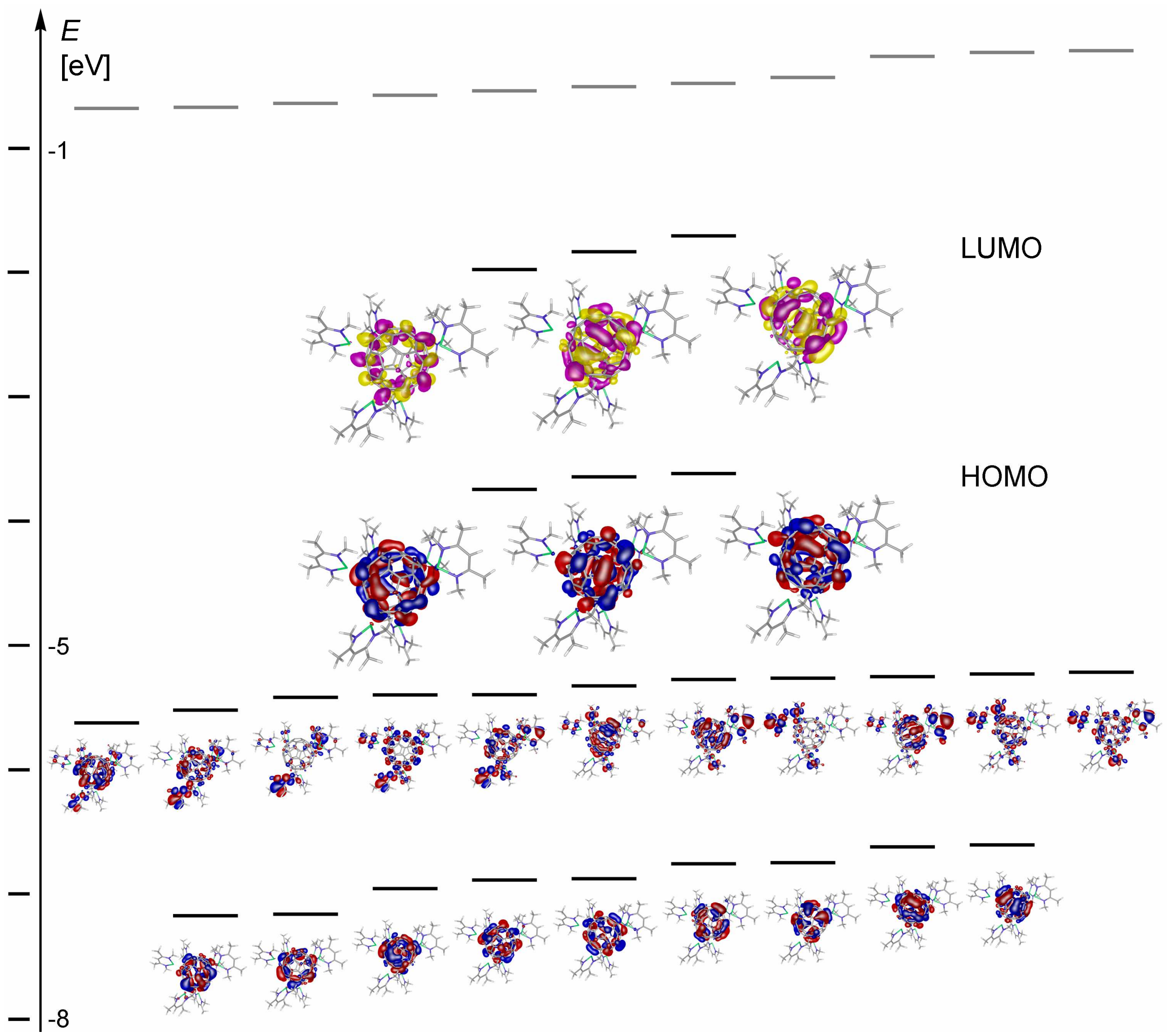

Figure S75. Partial molecular orbital diagram determined for **2Me-1** (pbe0/def2-svp with PCM (heptane), ultrafine grid) showing selected energy levels and molecular orbitals. Orbitals for filled levels are shown in blue and red, and in pink and yellow for unoccupied orbitals (isovalue 0.02; opacity 67%). The HOMO-LUMO gap is 0.0603 au (1.64 eV, 158 kJ/mol, 37.8 kcal/mol). Only the first 11 of 14 orbital energy levels (in grey, without orbital images) are shown for the LUMO+1 level band.

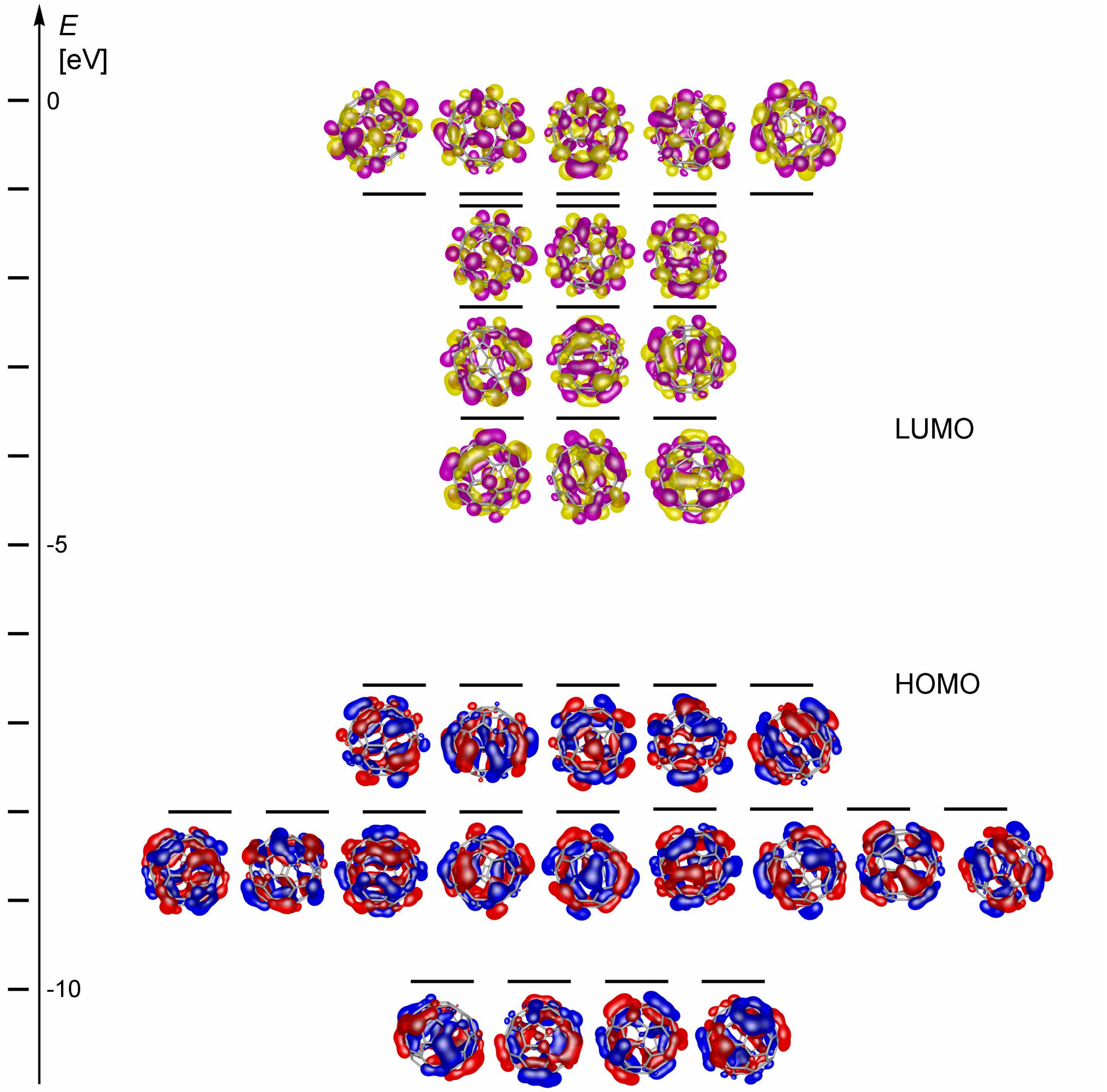

Figure S76. Partial molecular orbital diagram determined for C_{60} (pbe0/def2-svp with PCM (heptane), ultrafine grid) showing selected energy levels and molecular orbitals. Orbitals for filled levels are shown in blue and red, and in pink and yellow for unoccupied orbitals (isovalue 0.02; opacity 67%). The HOMO-LUMO gap is 0.110 au (3.00 eV, 289 kJ/mol, 69.2 kcal/mol).

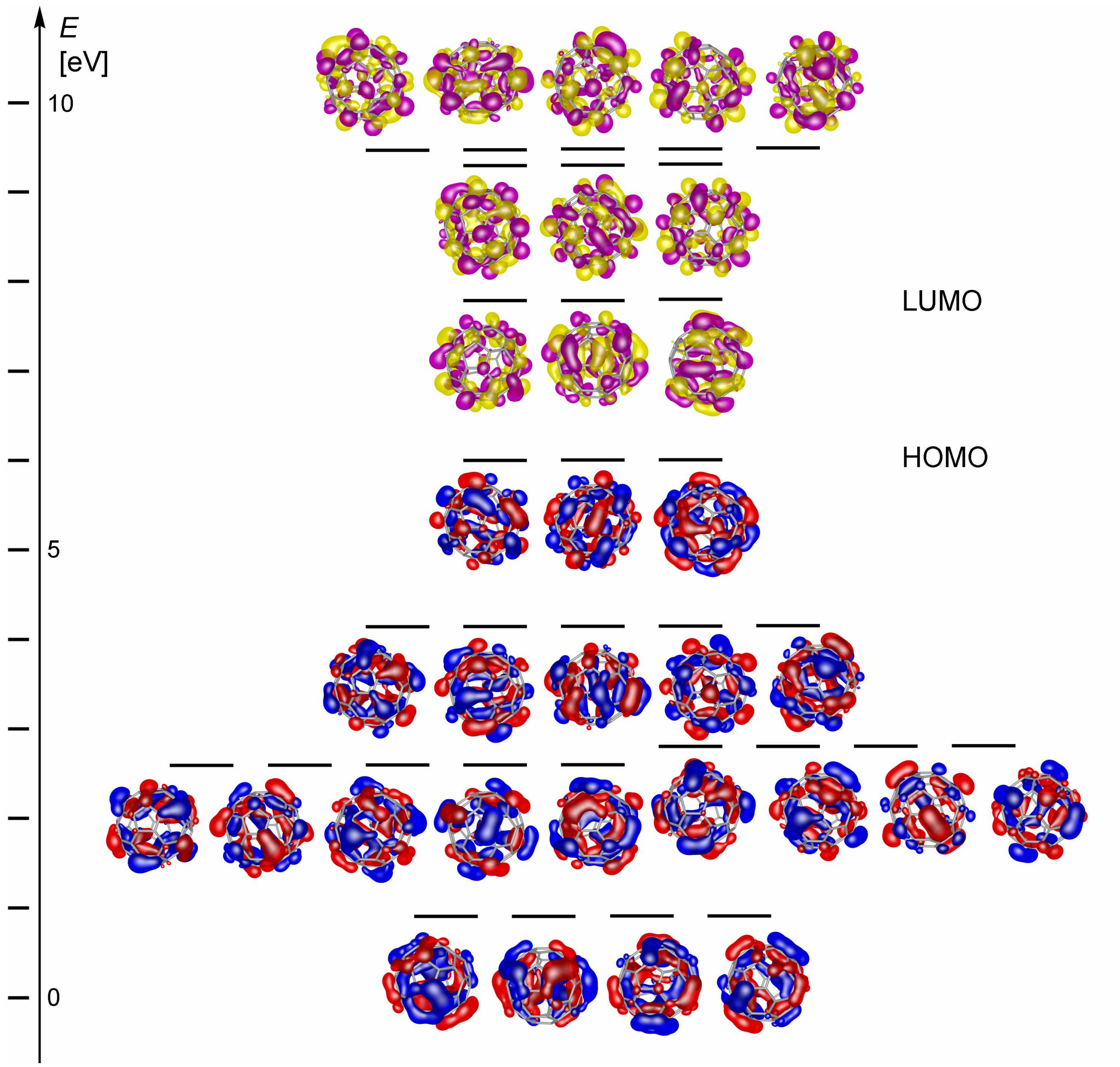

Figure S77. Partial molecular orbital diagram determined for C_{60}^{6-} (pbe0/def2-svp with PCM (heptane), ultrafine grid) showing selected energy levels and molecular orbitals. Orbitals for filled levels are shown in blue and red, and in pink and yellow for unoccupied orbitals (isovalue 0.02 ; opacity 67%). The HOMO-LUMO gap is 0.0653 au (1.78 eV, 172 kJ/mol, 41.0 kcal/mol). The gap between HOMO-1 and HOMO level is 0.0678 au (1.85 eV, 178 kJ/mol, 42.6 kcal/mol).

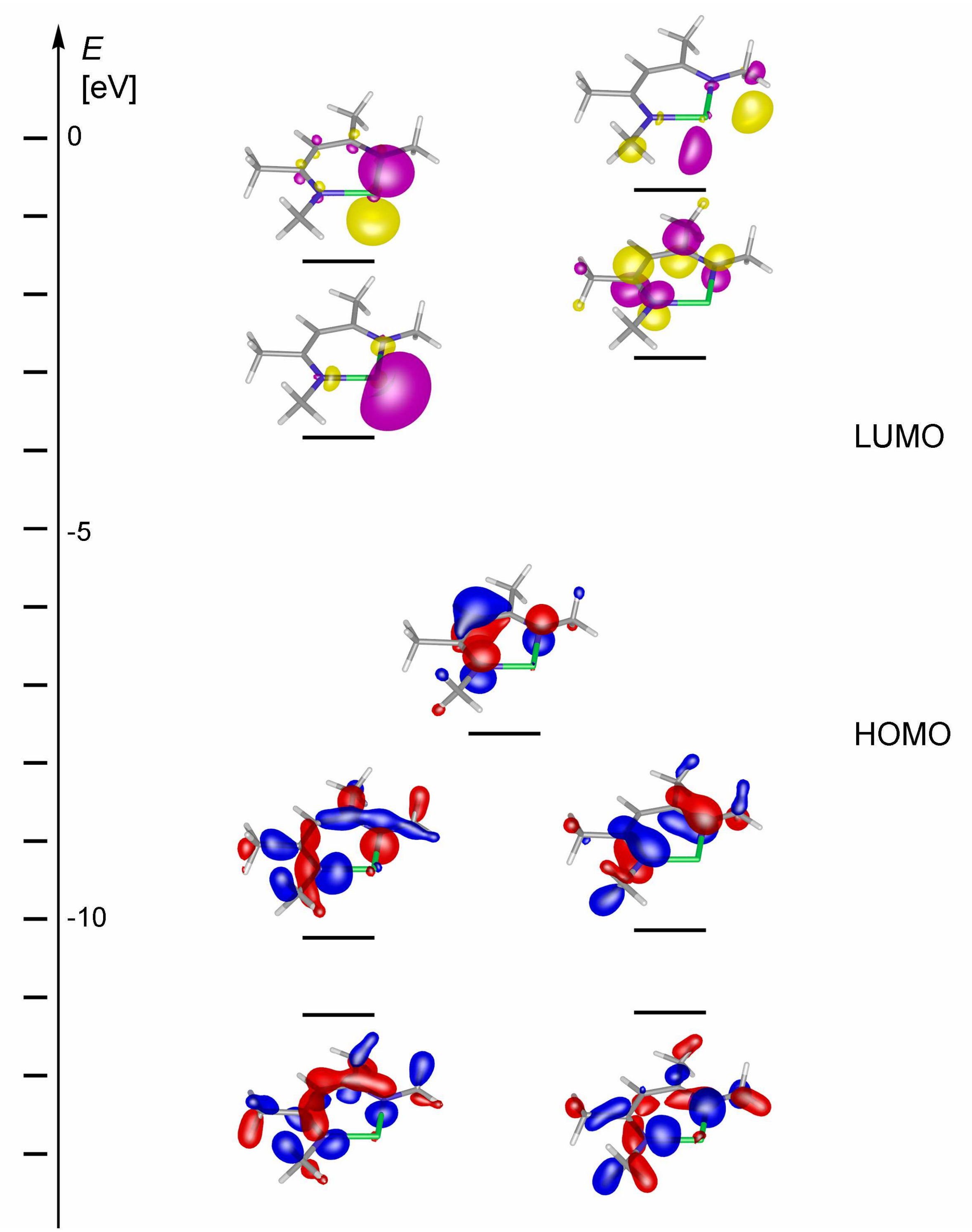

Figure S78. Partial molecular orbital diagram determined for [(^{Me}nacnac)Mg]$^+$ (pbe0/def2-svp with PCM (heptane), ultrafine grid) showing selected energy levels and molecular orbitals. Filled orbitals are shown in blue and red, and in pink and yellow for unoccupied orbitals (isovalue 0.06; opacity 67%). The HOMO-LUMO gap is 0.139 au (3.79 eV, 366 kJ/mol, 87.5 kcal/mol).

5 References

1 S. P. Green, C. Jones and A. Stasch, *Science*, 2007, **318**, 1754–1757.

2 R. Lalrempuia, C. E. Kefalidis, S. J. Bonyhady, B. Schwarze, L. Maron, A. Stasch and C. Jones, *J. Am. Chem. Soc.*, 2015, **137**, 8944–8947.

3 S. J. Bonyhady, C. Jones, S. Nembenna, A. Stasch, A. J. Edwards and G. J. McIntyre, *Chem. Eur. J.*, 2010, **16**, 938–955.

4 (a) S. J. Bonyhady, D. Collis, N. Holzmann, A. J. Edwards, R. O. Piltz, G, Frenking, A. Stasch and C. Jones, *Nat. Commun.*, 2018, **9**, 3079. (b) A. Stasch and C. Jones, *Dalton Trans.*, 2011, **40**, 5659–5672.

5 J. Hicks, M. Juckel, A. Paparo, D. Dange and C. Jones, *Organometallics*, 2018, **37**, 4810−4813.

6 H. Ajie, M. M. Alvarez, S. J. Anz, R. D. Beck, F. Diederich, K. Fostiropoulos, D. R. Huffman, W. Krätschmer, Y. Rubin, K. E. Schriver, D. Sensharma and R. L. Whetten, *J. Phys. Chem.*, 1990, **94**, 8630–8633.

7 *CrystalClear-SM Exper*t v2.1. Rigaku Americas, The Woodlands, Texas, USA, and Rigaku Corporation, Tokyo, Japan, 2015.

8 *CrysAlisPro* v1.171.38.46. Rigaku Oxford Diffraction, Rigaku Corporation, Oxford, U.K. 2015.

9 M. C. Burla, R. Caliandro, M. Camalli, B. Carrozzini, G. L. Cascarano, C. Giacovazzo, M. Mallamo, A. Mazzone, G. Polidori and R. Spagna, *J. Appl. Cryst*., 2012, **45**, 357–361.

10 L. Palatinus and G. Chapuis, *J. Appl. Cryst*., 2007, **40**, 786–790.

11 G. M. Sheldrick, *Acta Cryst.*, 2015, **A71**, 3–8.

12 G. M. Sheldrick, *Acta Cryst.*, 2015, **C71**, 3–8.

13 A. L. Spek, *Acta Cryst.*, 2015, **C71**, 9–18.

14 *CrystalStructure* v4.3.0. Rigaku Americas, The Woodlands, Texas, USA, and Rigaku Corporation, Tokyo, Japan, 2018.

15 O. V. Dolomanov, L. J. Bourhis, R. J. Gildea, J. A. K. Howard and H. Puschmann, *J. Appl. Cryst*., 2009, **42**, 339–341.

16 A. L. Spek, *Acta Crystallogr. Sect D.*, 2009, **65**, 148–155.

17 C. Adamo and V. Barone, *J. Chem. Phys.*, 1999, **110**, 6158–6159.

18 F. Weigend and R. Ahlrichs, *Phys. Chem. Chem. Phys.*, 2005, **7**, 3297–3305.

19 Gaussian 09, Revision D.01: M. J. Frisch, G. W. Trucks, H. B. Schlegel, G. E. Scuseria, M. A. Robb, J. R. Cheeseman, G. Scalmani, V. Barone, B. Mennucci, G. A. Petersson, H. Nakatsuji, M. Caricato, X. Li, H. P. Hratchian, A. F. Izmaylov, J. Bloino, G. Zheng, J. L. Sonnenberg, M. Hada, M. Ehara, K. Toyota, R. Fukuda, J. Hasegawa, M. Ishida, T. Nakajima, Y. Honda, O. Kitao, H. Nakai, T. Vreven, J. A. Montgomery Jr., J. E. Peralta, F. Ogliaro, M. Bearpark, J. Heyd, E.

Brothers, K. N. Kudin, V. N. Staroverov, R. Kobayashi, J. Normand, K. Raghavachari, A. Rendell, J. C. Burant, S. S. Iyengar, J. Tomasi, M. Cossi, N. Rega, J. M. Millam, M. Klene, J. E. Knox, J. B. Cross, V. Bakken, C. Adamo, J. Jaramillo, R. Gomperts, R. E. Stratmann, O. Yazyev, A. J. Austin, R. Cammi, C. Pomelli, J. W. Ochterski, R. L. Martin, K. Morokuma, V. G. Zakrzewski, G. A. Voth, P. Salvador, J. J. Dannenberg, S. Dapprich, A. D. Daniels, Ö. Farkas, J. B. Foresman, J. V. Ortiz, J. Cioslowski and D. J. Fox, Gaussian, Inc., Wallingford CT, 2009.

20 (a) K. L. Schuchardt, B. T. Didier, T. Elsethagen, L. Sun, V. Gurumoorthi, J. Chase, J. Li and T. L. Windus, *J. Chem. Inf. Model.*, 2007, **47**, 1045–1052. (b) D. Feller, *J. Comp. Chem.*, 1996, **17**, 1571–1586.

21 S. Grimme, S. Ehrlich and L. Goerigk, *J. Comp. Chem.*, 2011, **32**, 1456–1465.

22 NBO 6.0: E. D. Glendening, J. K. Badenhoop, A. E. Reed, J. E. Carpenter, J. A. Bohmann, C. M. Morales, C. R. Landis and F. Weinhold, Theoretical Chemistry Institute, University of Wisconsin, Madison, 2013; http://nbo6.chem.wisc.edu/.
